# Identification of cuproptosis-associated IncRNAs signature and establishment of a novel nomogram for prognosis of stomach adenocarcinoma

**DOI:** 10.3389/fgene.2022.982888

**Published:** 2022-09-09

**Authors:** Wei Yu, Hongqi Huo, Zhixin You, Rong Lu, Tianci Yao, Jing Huang

**Affiliations:** ^1^ Department of Pharmacy, Clinical Oncology School of Fujian Medical University, Fujian Cancer Hospital, Fuzhou, China; ^2^ Nuclear Medicine Department, HanDan Central Hospital, Handan, China; ^3^ Department of Laboratory Medicine, The First Affiliated Hospital of Xiamen University, Xiamen Key Laboratory of Genetic Testing, School of Medicine, Xiamen University, Xiamen, China; ^4^ Department of Pharmacy, The First Affiliated Hospital of Xiamen University, Xiamen, China

**Keywords:** risk score, prognosis, cuproptosis, immune, tumor

## Abstract

**Purpose:** Stomach adenocarcinoma (STAD) is one of the common cancers globally. Cuproptosis is a newly identified cell death pattern. The role of cuproptosis-associated lncRNAs in STAD is unknown.

**Methods:** STAD patient data from TCGA were used to identify prognostic lncRNAs by Cox regression and LASSO. A nomogram was constructed to predict patient survival. The biological profiles were evaluated through GO and KEGG.

**Results:** We identified 298 cuproptosis-related lncRNAs and 13 survival-related lncRNAs. Patients could be categorized into either high risk group or low risk group with 9-lncRNA risk model with significantly different survival time (*p* < 0.001). ROC curve and nomogram confirmed the 9-lncRNA risk mode had good prediction capability. Patients in the lower risk score had high gene mutation burden. We also found that patients in the two groups might respond differently to immune checkpoint inhibitors and some anti-tumor compounds.

**Conclusion:** The nomogram with 9-lncRNA may help guide treatment of STAD. Future clinical studies are necessary to verify the nomogram.

## 1 Introduction

Stomach adenocarcinoma (STAD) is frequently found in the digestive tract (Bray et al., 2018). It is mostly reported in eastern Asia and South America. There are several risk factors for STAD, including *Helicobacter pylori* infection, adenomatous gastric polyps, diet low in fruits and vegetables and diet high in cured or smoked foods (Wroblewski et al., 2010; Rawla and Barsouk, 2019; Akshatha et al., 2021). Although STAD is treatable surgically in its early stages, advanced STAD has a poor prognosis. Innovative therapeutics and prognostic models are both needed to improve prognosis of advanced STAD (Ajani et al., 2017; Ivey et al., 2022).

Metal micronutrients, especially iron (Fe), zinc (Zn), and copper (Cu), are essential for life. For example, Zn is involved in regulation of gene expression, and approximately 2,800 proteins may bind Zn *in vivo* (Andreini et al., 2006). Cu is catalyst or structural cofactor in many cellular activities, including mitochondrial respiration, immune function, and free radical scavenging (Festa and Thiele, 2011; Cobine et al., 2021). Despite its role for normal life, high serum copper level has been linked to increased risk of cancer (Brady et al., 2014; Tsang et al., 2020) and atherosclerotic diseases (Reunanen et al., 1992; Ford, 2000; Chen et al., 2015).

Mostly recently Tsvetkov et al. (2022) demonstrated a novel mechanism of Cu-induced cell death that is related to mitochondria dysfunction. This novel form of regulated cell death was termed “cuproptosis”. Cuproptosis may happen when mitochondrial enzymes aggregate and leads to mitochondrial stress. Cuproptosis is different from apoptosis, ferroptosis, or necroptosis. This discovery suggests mitochondrial Cu homeostasis may be exploited for cancer therapy.

Here we explored whether cuproptosis-related lncRNAs may be involved in STAD patient prognosis. The results might help understand the roles of cuproptosis in the development and progression of STAD.

## 2 Materials and methods

### 2.1 TCGA data

We downloaded RNA sequencing (RNA-seq) and expression files and mutation files from the Cancer Genome Atlas (TCGA) database (https://portal.gdc.cancer.gov/repository). The data included tumor tissues of 343 STAD patients and 30 matched normal tissues. Data were downloaded and handled according to TCGA guidelines.

### 2.2 Identification of cuproptosis-related lncRNAs

According to the study by Tsvetkov et al. (2022), 19 cuproptosis-associated genes were evaluated (Supplementary Table S1). Correlation between cuproptosis-related genes and differentially expressed lncRNAs was evaluated. Pearson’s correlation coefficients (R) of gene expression patterns were used as a measure of gene coexpression. The PCC threshold to retrieve cuproptosis-related lncRNAs was 0.4 (|R| > 0.4), with a *p* value < 0.001.

### 2.3 Cuproptosis-related lncRNAs signature for STAD prognosis

The downloaded clinical and demographic data of STAD patients were analyzed with univariate Cox regression analysis to identify lncRNAs associated with patient overall survival (OS) and those associated with cuproptosis were further identified as candidate lncRNAs for the construction of prognostic signature. Lasso regression was performed to screen lncRNAs that were truly correlated with a patient’s survival on the basis of 10-fold cross-validation. Based on the nine optimal lncRNAs identified, the risk scores of patients were calculated according to the following formula:

$$risk\;score=\overset{n}{\underset{i}{\sum }}Xi*Yi$$

Where X was regression coefficient and Y was expression level of cuproptosis-related lncRNAs.

A total of 343 STAD patients were allocated to either the training cohort or the test cohort randomly in a 1:1 ratio for constructing and validating the cuproptosis-related lncRNAs signature. Patients in each cohort were classified into either low--risk group or high-risk group according to the cut-off value, which was the median risk score (Meng et al., 2019; Hong et al., 2020). The Chi-square test and the receiver operating characteristics (ROC) curves were used to help determine if observed OS was in line with expected OS, and the 1-year, 3-years, and 5-years OS rates were compared between the low-risk group and the high-risk group by Kaplan–Meier analysis. We further constructed a nomogram with cuproptosis-related lncRNA risk score and established clinical risk factors to calculated patient survival time. Then concordance index (C-index) and calibration curves were used to evaluate the prediction power of the nomogram. Finally, stratified analysis was used to assess whether the signature retained its predictive ability in subgroups of patients (stages I–II and stages III–IV). The “survival”, “rms”, “survminer” and “timeROC” R packages were used.

### 2.4 Principal component analysis, gene ontology and gene set enrichment analysis

We used principal component analysis (PCA) to characterize cuproptosis-related lncRNAs expression patterns. PCA is a common unsupervised method for the analysis of gene expression data. 3D scatter plots were used to visualize the relationship between the three variables of samples. The analysis of differentially expressed genes (DEGs) was performed with the glm method of the “edgeR” R package. We set the threshold value of log fold change (log2FC) at |log2FC| ≥ 1, with a false discovery rate (FDR) < 0.05, to identify important DEGs. Gene Ontology (GO) was used to interpret DEGs for the relevant cellular components, biological processes, and molecular functions. Differential Kyoto Encyclopedia of Genes and Genomes (KEGG) pathways between the high-risk group and the low-risk group were screened using Gene Set Enrichment Analysis (GSEA), with a FDR <0.25.

### 2.5 Immune function

Single-sample GSEA (ssGSEA), an extension of GSEA, was used to calculate separate enrichment scores for immunological pathways by the normalized enrichment score (NES) (Subramanian et al., 2005). Each ssGSEA enrichment score represents the degree to which the genes are coordinately upregulated or downregulated within a sample.

### 2.6 Tumor mutation burden

We downloaded the somatic mutation file and calculated each patient’s tumor mutation burden (TMB) score. The influence of TMB on patient OS was evaluated by Kaplan–Meier analysis and compared between the high- and low-risk groups by *t*-test. Maftools R package was used.

### 2.7 Tumor immune dysfunction and exclusion score and drug sensitivity prediction

To predict treatment response of immune checkpoint blockades (ICBs), tumor immune dysfunction and exclusion (TIDE) algorithm was used to identify signatures of T cell dysfunction and signatures that exclude T cell infiltration into tumors (Jiang et al., 2018). To predict treatment response of the most important groups of drugs again STAD, the half-maximal inhibitory concentrations (IC_50_) were calculated using pRRophetic as described in Genomics of Drug Sensitivity in Cancer (GDSC) (Geeleher et al., 2014).

## 3 Results

### 3.1 lncRNAs data

**FIGURE 1:**
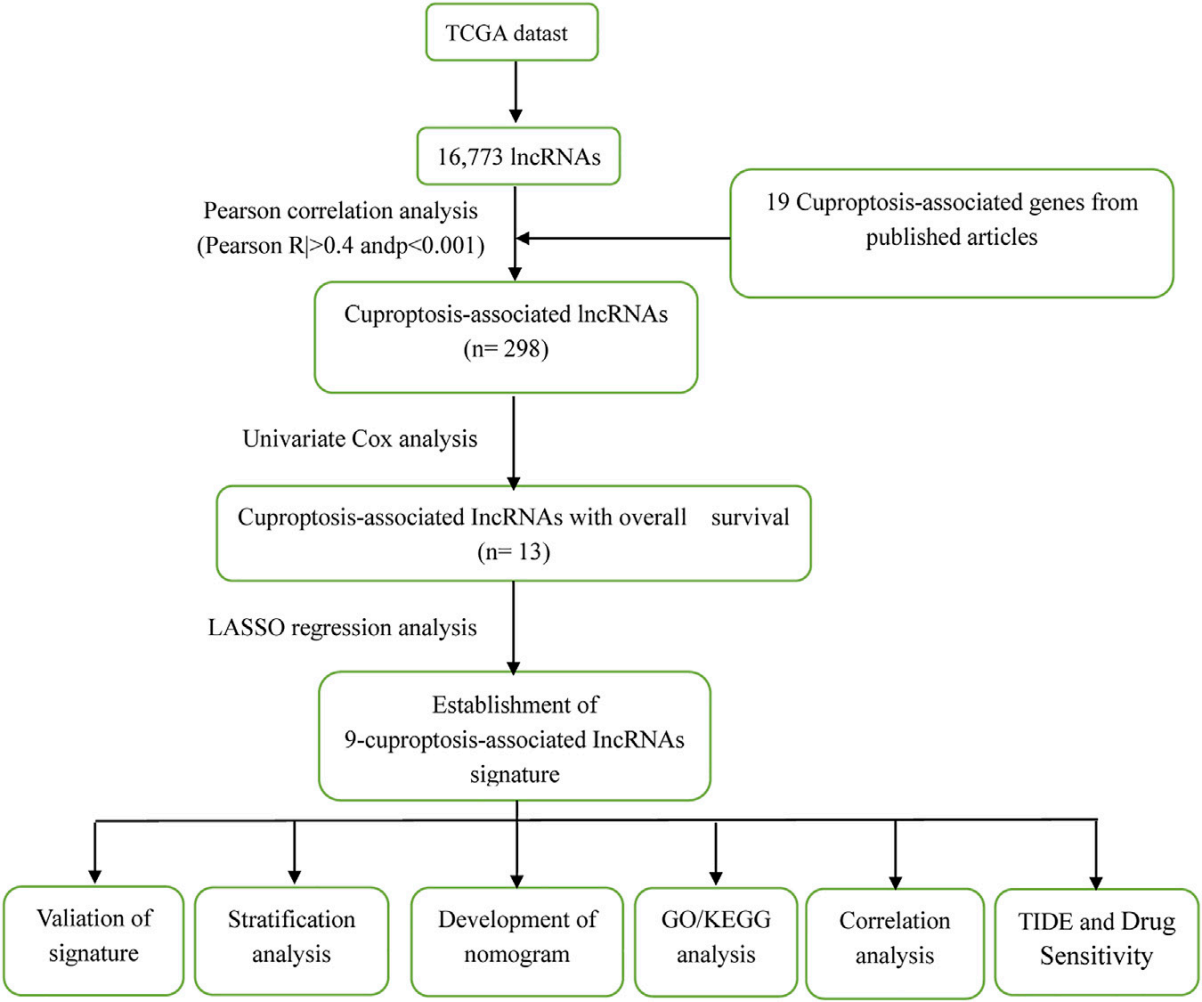
The process of the study.

**FIGURE 2:**
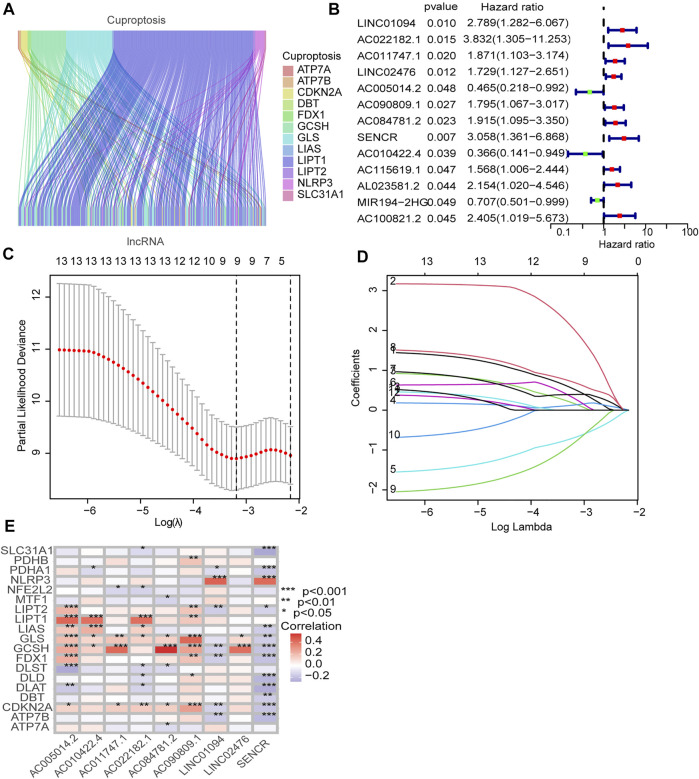
Identification of prognostic cuproptosis-related lncRNAs in STAD. **(A)** The Sankey diagram demonstrates correlation between cuproptosis-related lncRNAs and cuproptosis-related genes. **(B)** The prognostic lncRNAs identified by uni-Cox regression analysis. **(C)** LASSO model, with a 10-fold cross-validation. **(D)** The coefficient profile of nine lncRNAs screened by the LASSO model. **(E)** Correlations between lncRNAs in the risk model and cuproptosis-related genes.

Figure 1 illustrates the results of the search and the process of screening. A total of 16,773 lncRNAs that may be associated with 19 cuproptosis-associated genes were found. Among these lncRNAs, 298 lncRNAs met the pre-defined criteria ((|R| > 0.4). All 298 lncRNAs upregulated the expression of cuproptosis genes in the Sankey diagram (Figure 2A. Univariate Cox regression analysis found that 13 lncRNAs were prognostic factors of patient survival (Figure 2B).

### 3.2 Risk model

To construct a risk model with cuproptosis-related lncRNAs in STAD, we randomly allocated 343 STAD cases into the training set and the test set at 1:1 ratio. The chi-square test showed that the two groups were comparable in terms of both clinicopathologic and demographic parameters (Table 1).

**TABLE 1:** Clinicopathologic and demographic characteristics of STAD patients in the training and test cohorts.

Variable	Total	Training cohort	Test cohort	*p* value
≤65	72 (42.6%)	38 (44.71%)	34 (40.48%)	0.6889
>65	97 (57.4%)	47 (55.29%)	50 (59.52%)	
Female	69 (40.83%)	30 (35.29%)	39 (46.43%)	0.1882
Male	100 (59.17%)	55 (64.71%)	45 (53.57%)	
G1	3 (1.78%)	2 (2.35%)	1 (1.19%)	0.5907
G2	71 (42.01%)	33 (38.82%)	38 (45.24%)	
G3	92 (54.44%)	49 (57.65%)	43 (51.19%)	
Unknown	3 (1.78%)	1 (1.18%)	2 (2.38%)	
Stage I	24 (14.2%)	10 (11.76%)	14 (16.67%)	0.8193
Stage II	46 (27.22%)	23 (27.06%)	23 (27.38%)	
Stage III	68 (40.24%)	35 (41.18%)	33 (39.29%)	
Stage IV	20 (11.83%)	11 (12.94%)	9 (10.71%)	
Unknown	11 (6.51%)	6 (7.06%)	5 (5.95%)	
T1	13 (7.69%)	8 (9.41%)	5 (5.95%)	0.1533
T2	29 (17.16%)	9 (10.59%)	20 (23.81%)	
T3	74 (43.79%)	39 (45.88%)	35 (41.67%)	
T4	47 (27.81%)	25 (29.41%)	22 (26.19%)	
Unknown	6 (3.55%)	4 (4.71%)	2 (2.38%)	
N0	50 (29.59%)	20 (23.53%)	30 (35.71%)	0.0402
N1	45 (26.63%)	27 (31.76%)	18 (21.43%)	
N2	31 (18.34%)	12 (14.12%)	19 (22.62%)	
N3	34 (20.12%)	22 (25.88%)	12 (14.29%)	
Unknown	9 (5.33%)	4 (4.71%)	5 (5.95%)	
M0	148 (87.57%)	75 (88.24%)	73 (86.9%)	1
M1	14 (8.28%)	7 (8.24%)	7 (8.33%)	
Unknown	7 (4.14%)	3 (3.53%)	4 (4.76%)	

To avoid overfitting, nine lncRNAs were further identified by LASSO regression method (Figures 2C,D. A formula was established with the expression levels of nine lncRNAs:

Risk score = LINC01094 × (0.5250) + AC022182.1 × (2.02146) + AC011747.1 × (0.1655) + LINC02476 × (0.1295) + AC005014.2 × (−0.6903) + AC090809.1 × (0.2959) + AC084781.2 × (0.3942) + SENCR × (0.6958) + AC010422.4 × (−0.8166) (Meng et al., 2019).

**FIGURE 3:**
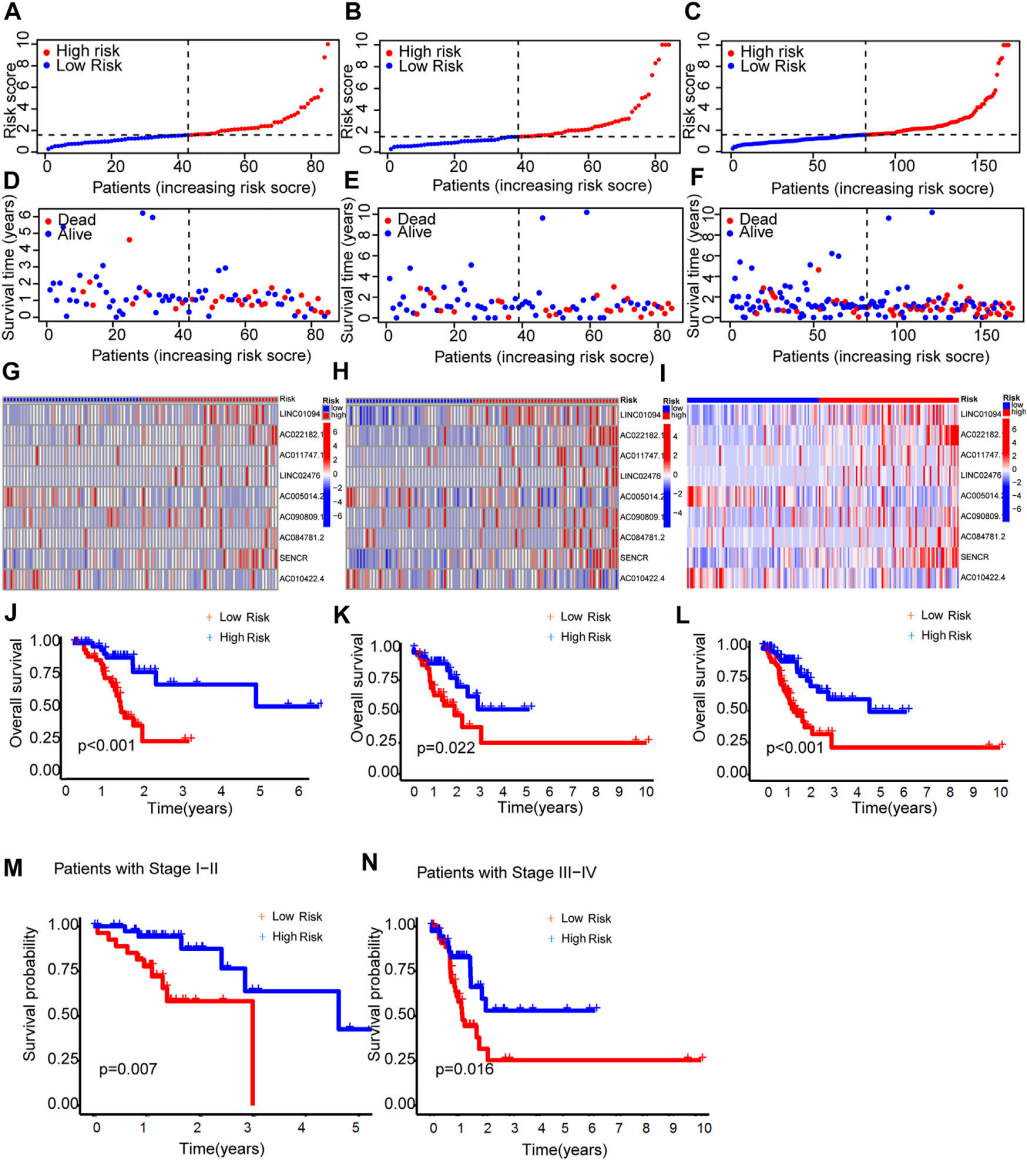
Prognosis capability of the model in the three patient sets. **(A–C)** Distribution of patient with different scores. **(D–F)** Distribution of patient survival time. **(G–I)** The heatmap of nine lncRNAs expression. **(J–L)** Comparison of OS curves of patients between the two groups of each set. **(M,N)** OS curves of stratified by clinicopathologic characteristics in the entire set.

As expected, the high-risk group had worse survival in each sample set (Figure 3).

### 3.3 Assessment of the risk model

**FIGURE 4:**
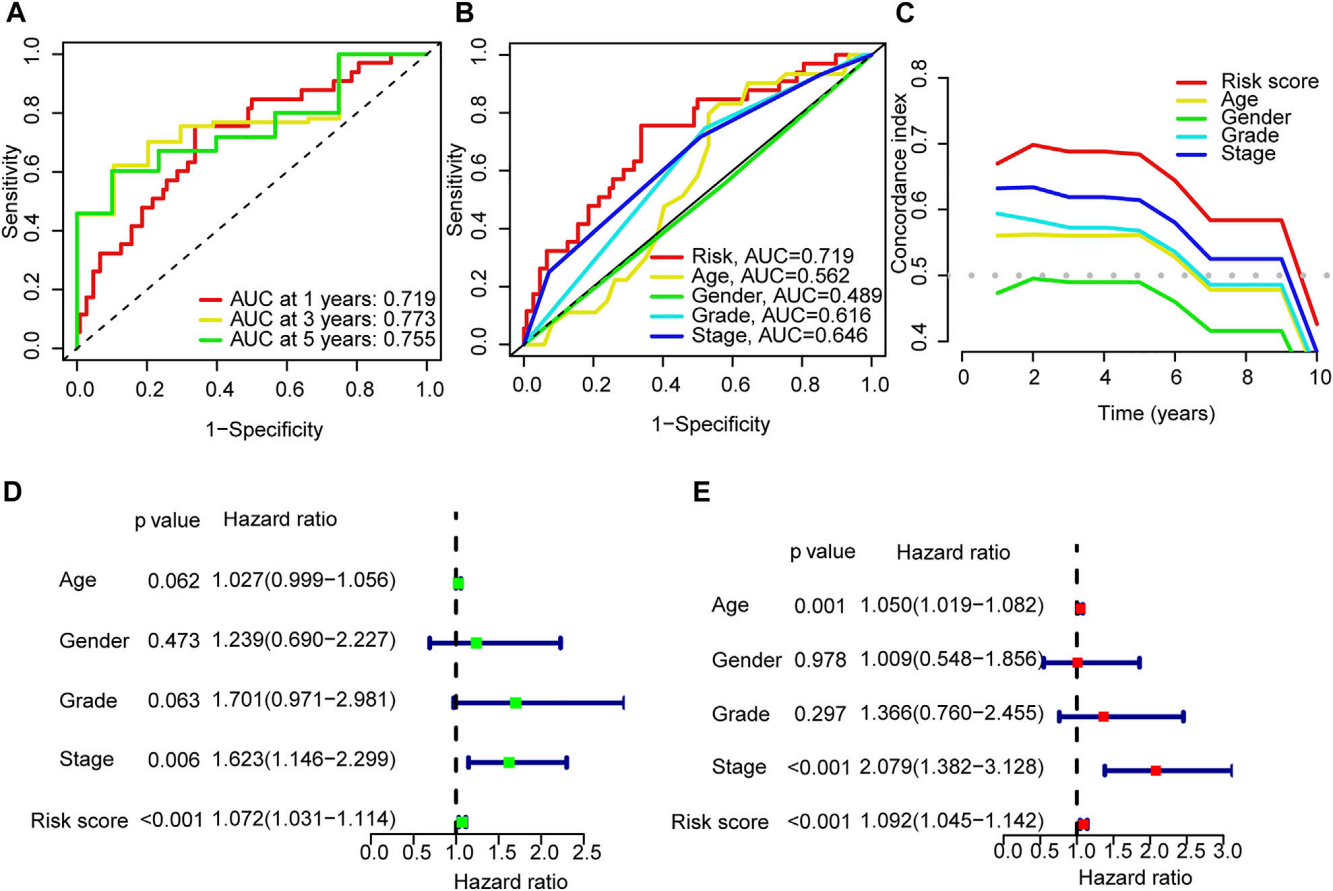
Validation of the model. **(A)** Time-dependent ROC curve analyses for survival of all patients based on the risk score model. **(B)** Comparision of the ROC curves of risk score, patient age, patient gender, tumor grade and tumor stage. **(C)** The C-index curves of risk score, patient age, patient gender, tumor grade and tumor stage. **(D,E)** Uni-Cox and multi-Cox analyses of overall survival for risk score, patient age, patient gender, tumor grade and tumor stage.

The areas under the l-, 3- and 5-years ROC curves (AUC) were 0.719, 0.773, and 0.755 respectively (Figure 4A). The AUC of risk score was 0.719 and the C-index in the risk model was 0.726, indicting a perfect predictive ability (Figures 4B,C). In the uni-Cox regression, the hazard ratios (HR) of the risk score was 1.0726 (*p* < 0.001), and in the multi-Cox regression, HR of the risk score was 1.092 (*p* < 0.001) (Figures 4D,E).

### 3.4 Nomogram

**FIGURE 5:**
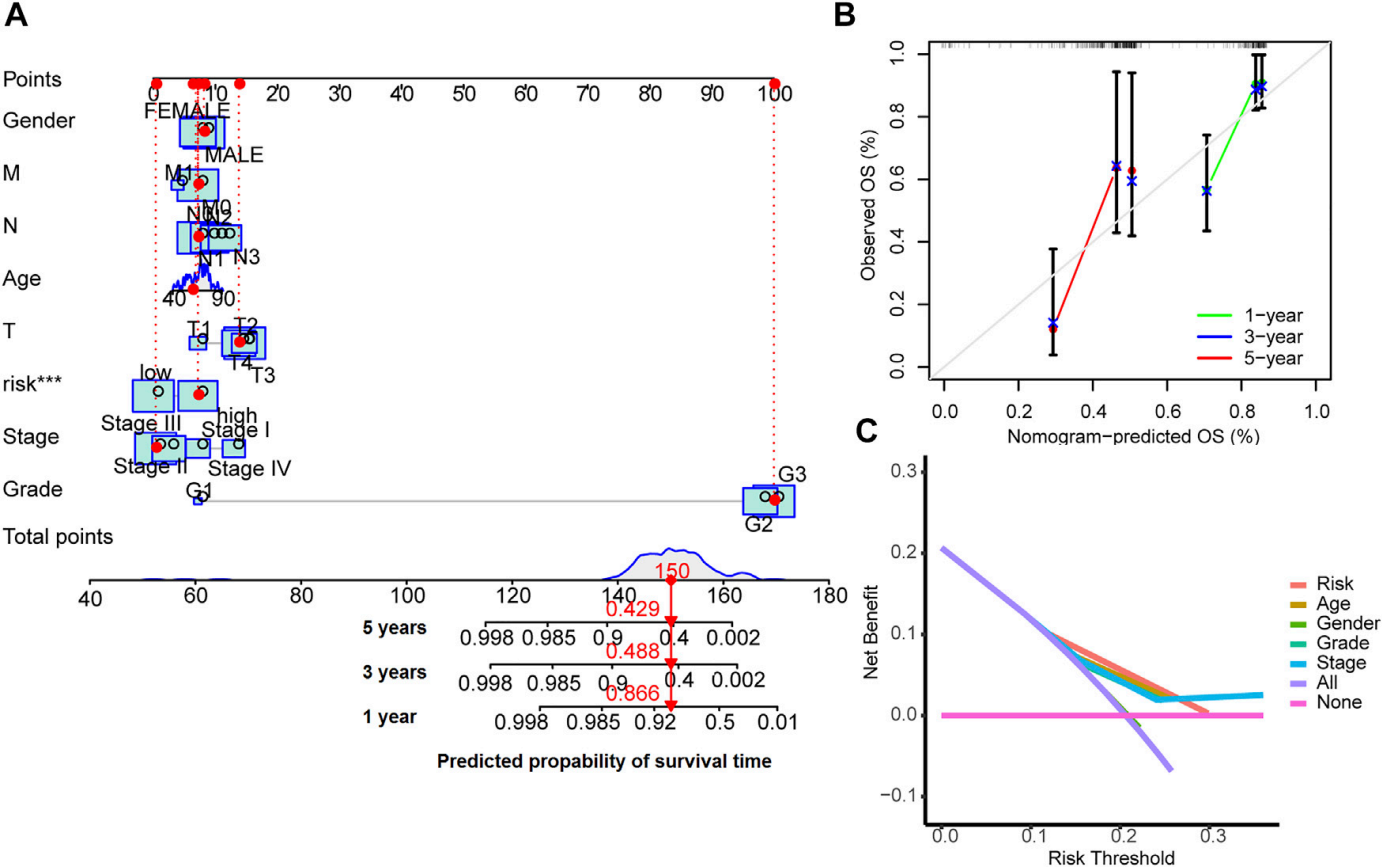
Nomogram for survival prediction **(A)**, the calibration curves **(B)** and the decision curves **(C)**.

A nomogram model was drawn to predict OS of patients (Figure 5A). The calibration plots showed the predicted l-, 3- and 5-years OS was consistent with the actual OS (Figure 5B). Thus the nomogram was well calibrated, with good prediction of patient survival. The high value of C index (0.726) indicated that the nomogram has excellent discriminative ability.

The results of decision curve analyses to compare the performance of the nomogram are shown in Figure 5C. The nomogram has greater net benefit than other clinical parameters in all patients.

### 3.4 PCA and biological pathways analyses

**FIGURE 6:**
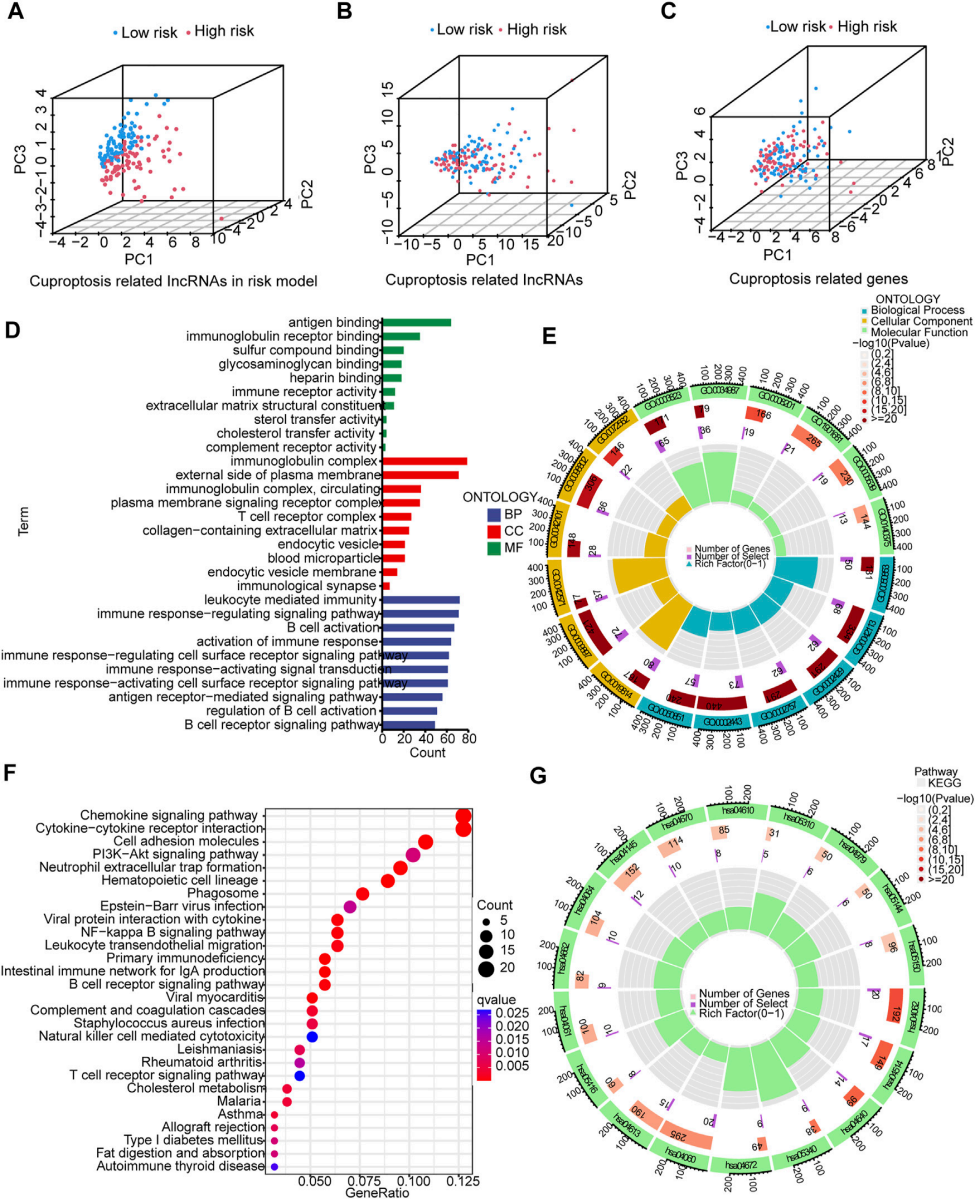
PCA, GO, and KEGG analyses. **(A–C)** 3D scatter plots of sample distribution. **(D,E)** GO analysis of biological processes, cellular components and molecular functions. **(F,G)** KEGG analysis of PI3K−Akt signaling pathway, cell adhesion, cytokine−cytokine receptor interaction and chemokine signaling pathway.

The 3D scatter diagram showed the low-risk group and the high-risk group had distinct aggregation features of PCA (Figures 6A–C). GO analysis indicated related biological processes included B cell activation signaling pathway, antigen receptor−mediated signaling pathway, and immune response−regulating signaling pathway; related cellular components included immunological synapse, endocytic vesicle membrane, endocytic vesicle, T cell receptor complex, and immunoglobulin complex, and related molecular functions included immune receptor activity, heparin binding, glycosaminoglycan binding, sulfur compound binding, immunoglobulin receptor binding, and antigen binding (Figures 6D,E). GSEA identified genes involved in PI3K−Akt signaling pathway, cell adhesion, cytokine−cytokine receptor interaction and chemokine signaling pathway were differentially expressed between the low--risk group and high-risk group (Figures 6F,G).

### 3.5 Correlation analysis between risk scores and gene mutations

**FIGURE 7:**
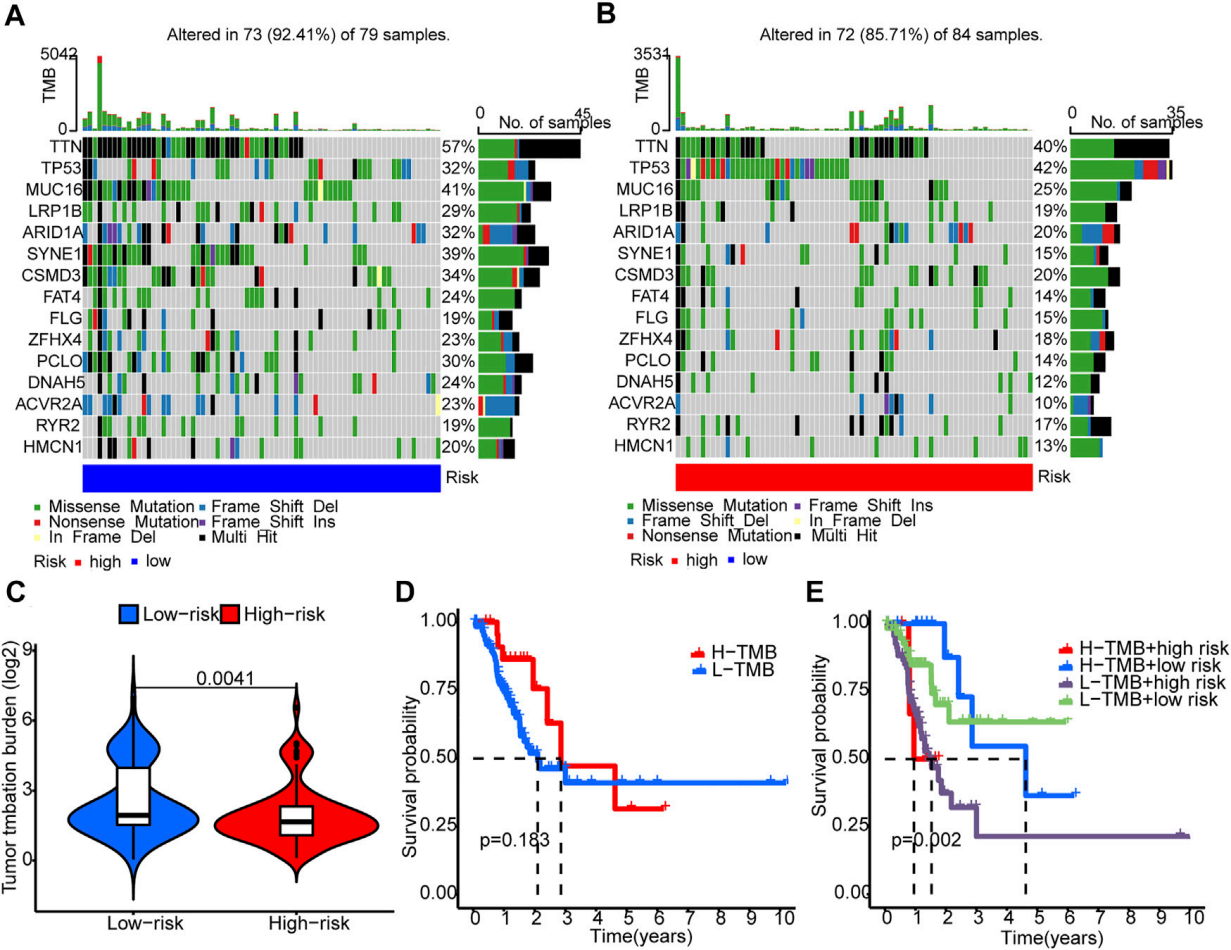
Tumor mutation burden (TMB). **(A,B)** The waterfall plots illustrates the frequencies of mutations of genes with different colors representing different types of mutations. **(C)** There were significantly higher TMB in the low-risk group compared to the high-risk group. **(D)** K-M survival curves show similar patient survival between the high- and the low-TMB groups. **(E)** K-M survival curves show different patient survival among the four groups.

Somatic mutations between the two groups were compared. The ten most mutated genes were *TP53, TTN, PCLO, ZFHX4, CSMD3, SYNE1, ARID1A, LRP18, MUC16,* and *ACVR2A*. The high-risk group had more frequent *TP53* mutation (Figures 7A,B) but overall lower TMB (Figure 7C). Patients with higher scores and lower TMB had the worst prognosis among the four groups (Figures 7D,E).

### 3.6 TIDE, immune functions and prediction of clinical treatment response

**FIGURE 8:**
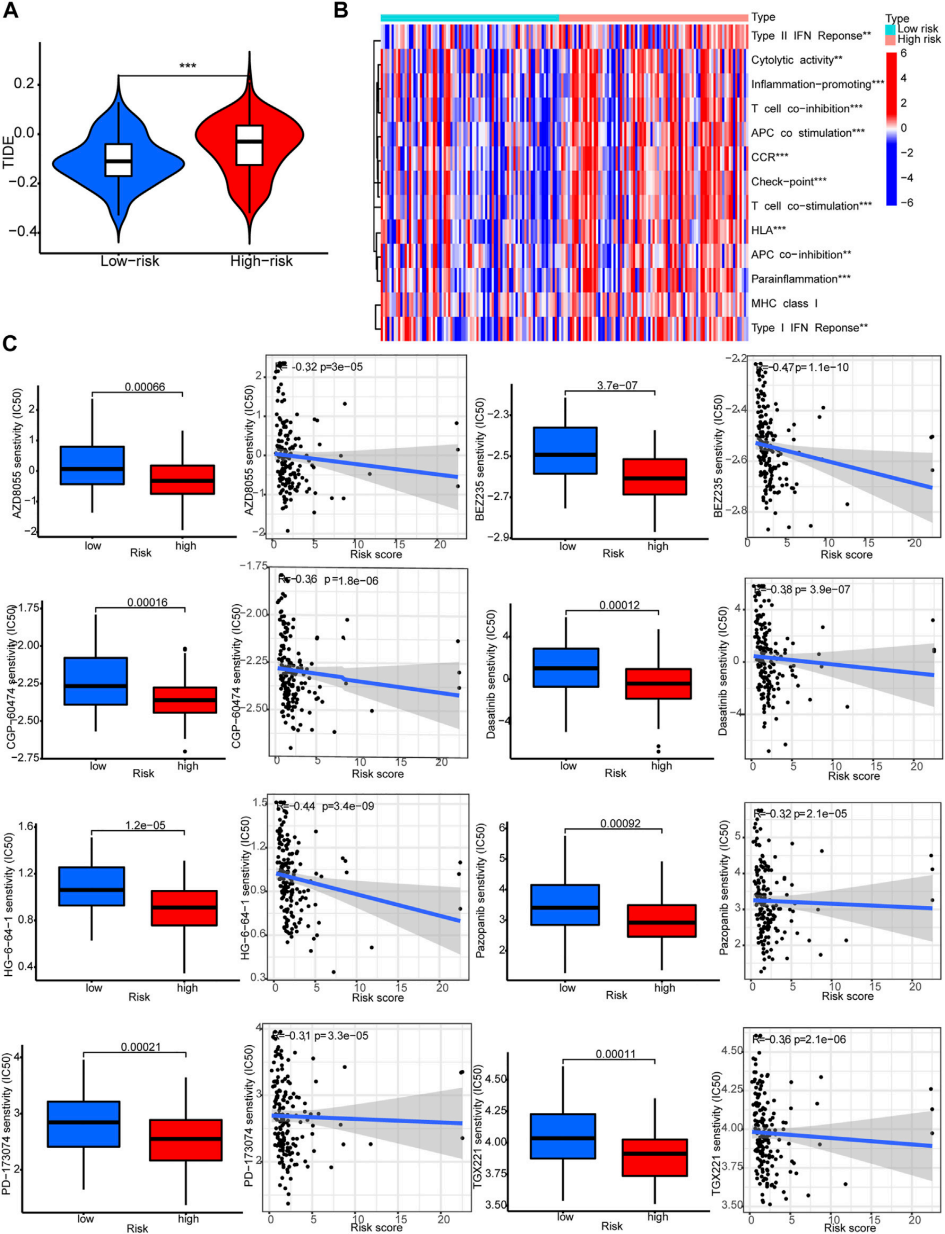
Immune functions and prediction of clinical treatment response. **(A)**TIDE scores. **(B)** Immune function heat maps. **(C)** IC_50_ of eight drugs.

The TIDE scores were significantly higher in the high-risk group compared to the low-risk group. This indicated that TIDE could be used to evaluate sensitivity to ICB therapy for STAD patients (Figure 8A). Indeed, several immune-related pathways had different activities between the two groups. Patients in the high-risk group had higher activities in terms of T cell co−inhibition and check−point (Figure 8B). Drug sensitivity comparison showed most drugs have similar IC_50_ between the two groups, and there were eight drugs that had lower IC_50_ in the high-risk group: PD−173,074, AZD8055, BEZ235, CGP-60474, Dasatinib, Pazopanib, TGX221, and HG-6-64-1 (Figure 8C).

## 4 Discussion

STAD is a common malignancy worldwide. Although the mortality of STAD has declined due to earlier detection and treatment advancement including targeted therapy, the OS of STAD patients remains low due to delayed diagnoses that makes tumor unresectable. The copper level has been reported to be increased in cancer patients, which could promote tumor angiogenesis, progression and metastasis. Recently Tsvetkov et al. reported cuproptosis, a novel form of regulated cell death (Tsvetkov et al., 2022). Investigation of cuproptosis-related genes in cancer could help understand mechanisms of tumor development. The identification of cuproptosis may also promote innovations in the development new anti-cancer agents.

Biomarkers, including genetic and epigenetic ones, are playing a crucial role in cancer treatment and prognosis (Mishra and Verma, 2010). For example, the TCGA project classify STAD into four major subtypes with different genomic profiles to guide targeted therapy (2014). Non-coding RNA transcripts, such as lncRNAs, can also be used as biomarkers because lncRNAs might regulate cancer development (Djebali et al., 2012; Lee, 2012; Huarte, 2015; Marchese et al., 2017; Mattick, 2018). With the abundant novel lncRNAs identified recently, the annotation of these lncRNAs is urgently needed. We found that nine cuproptosis-related lncRNAs were related to survival of STAD patients. On one hand, LINC01094, AC022182.1, AC011747.1, LINC02476, AC090809.1, AC084781.2, and SENCR were risk factors for STAD patients. On the other hand, AC010422.4 and AC005014.2 were protective factors for STAD patients. The underlying mechanisms for the relationship between STAD prognosis and expression levels of LINC01094, AC022182.1, AC011747.1, LINC02476, AC090809.1, AC084781.2, SENCR, AC010422.4 and AC005014.2 are unknown presently.

Several studies have reported that LINC01094 was associated with diverse tumors. Jiang et al. (2020) found that LINC01094 expression was upregulated in clear cell renal cell carcinoma (ccRCC) in the TCGA database and ccRCC cell lines. LINC01094 knockdown inhibited ccRCC cell growth and metastasis via binding miR-224-5p. Increased expression of LINC01094 was also found in glioma, and was associated with glioma grade. LINC01094 bound to miR-330-3p in glioma (Zhu et al., 2020). In ovarian cancer, LINC01094 expression was elevated and was related to FIGO stage and lymph node metastasis. LINC01094 expression was also a risk factor for ovarian cancer patient survival. In ovarian cancer cells, LINC01094 bound to miR-577 and increased cell proliferation, migration, and the expressions of β-catenin, c-Myc and cyclin D1 (Xu et al., 2020). In colorectal cancer, LINC01094 was also highly expressed and correlated with lymph node metastasis and TNM stage. LINC01094 promoted proliferation, invasion, and migration of colorectal cancer cells by sponging miR-1266-5p (Zhang et al., 2022). Thus LINC01094 is an oncogene in an array of tumors.

SENCR (Smooth Muscle And Endothelial Cell Enriched Migration/Differentiation-Associated LncRNA) is a super enhancer lncRNA originally reported to be overexpressed in smooth muscle cells and endothelial cells. SENCR promoted proliferation, differentiation, and migration of endothelial cells (Bell et al., 2014; Boulberdaa et al., 2016; Sun et al., 2018). Studies have found that SENCR is closely related to the progress of several human cancers. Non-small cell lung cancer (NSCLC) had higher expression of SENCR. Knockdown of SENCR inhibited the growth and metastasis of NSCLC through miR-1-3p. SENCR increased CDK4 and CDK6 expression by binding to miR-1-3p (Cheng et al., 2021). Knockdown of SENCR in cisplatin-resistant A549 cell reduced cell proliferation, accompanied by decreased levels of proteins PCNA, MDMX, and P-gp and increased apoptosis. Overexpressing SENCR could increase FLI1 expression (Shen et al., 2022).

When we compared somatic mutations between the two groups, we found mutations were more frequent in the high-risk group. *TP53* mutations are very common in cancers, ranging from 38% to 50% in a variety of solid tumors and in about 5% of primary leukemia. Germline mutations of *TP53* are the underlying cause of Li-Fraumeni syndrome with early-onset cancers. *TP53* (Correa, 2016) mutations may caused by chemical damage induced by particular mutagens, including environmental agents. We propose that higher level of Cu in cancer patients may induce *TP53* mutations, which may related to cuproptosis.

GSEA identified genes of PI3K−Akt signaling pathway might be differentially expressed between the low-risk group and the high-risk group. The PI3K/AKT signaling pathway regulates cell survival and proliferation. Aberrant activation of the pathway is often associated with tumor progression and resistance to cancer therapies (LoRusso, 2016). Thus the relationship between PI3K−Akt signaling pathway and cuproptosis deserves further studies.

We predicted treatment response of the drugs again STAD using pRRophetic (Geeleher et al., 2014) and found that cuproptosis may be related to drug sensitivity. Indeed, Tsvetkov et al. (2022) reported the hydrophilic antioxidant glutathione (GSH) blocked the toxicity of elesclomol (ES)-Cu by chelating intracellular Cu. They also found that NCIH2030 lung cancer cells that rely on galactose-mediated mitochondrial respiration were much more sensitive to ES-Cu-induced growth inhibition than cells that rely on glucose-induced glycolysis. The depletion of GSH by buthionine sulfoximine also increased susceptibility to cuproptosis in A549 lung cancer cells. Thus it is reasonable to expect that drugs involved in galactose regulation pathways may have different effects on cancer cells with different expression of cuproptosis-related genes.

To conclude, we constructed a nomogram exploiting cuproptosis-associated lncRNA expression to predict survival of patients with STAD. Cu is a crucial metal with redox properties. Depending on it’s concentration in cells, Cu may be either beneficial or toxic to the cell. Further studies of the roles of Cu in cancer development will lead to more innovative therapies (Ge et al., 2022). The usefulness of this nomogram in predicting patient survival and in treatment decision-making need to be explored in the future studies.

## Data Availability

The original contributions presented in the study are included in the article/Supplementary Material, further inquiries can be directed to the corresponding authors.
