# Supplementary material for: Identification of cuproptosis-associated IncRNAs signature and establishment of a novel nomogram for prognosis of stomach adenocarcinoma
Source: Front Genet. 2022 Sep 9;13:982888. doi: 10.3389/fgene.2022.982888 (PMC9504471; doi:10.3389/fgene.2022.982888)
Supplement: Supplementary file 2 [file Table1.DOC]

Cuproptosis-associated Genes

NFE2L2

NLRP3

ATP7B

ATP7A

SLC31A1

FDX1

LIAS

LIPT1

LIPT2

DLD

DLAT

PDHA1

PDHB

MTF1

GLS

CDKN2A

DBT

GCSH

DLST
