# Supplementary figures and images for: Identification of cuproptosis-associated IncRNAs signature and establishment of a novel nomogram for prognosis of stomach adenocarcinoma
Source: Front Genet. 2022 Sep 9;13:982888. doi: 10.3389/fgene.2022.982888 (PMC9504471; doi:10.3389/fgene.2022.982888)

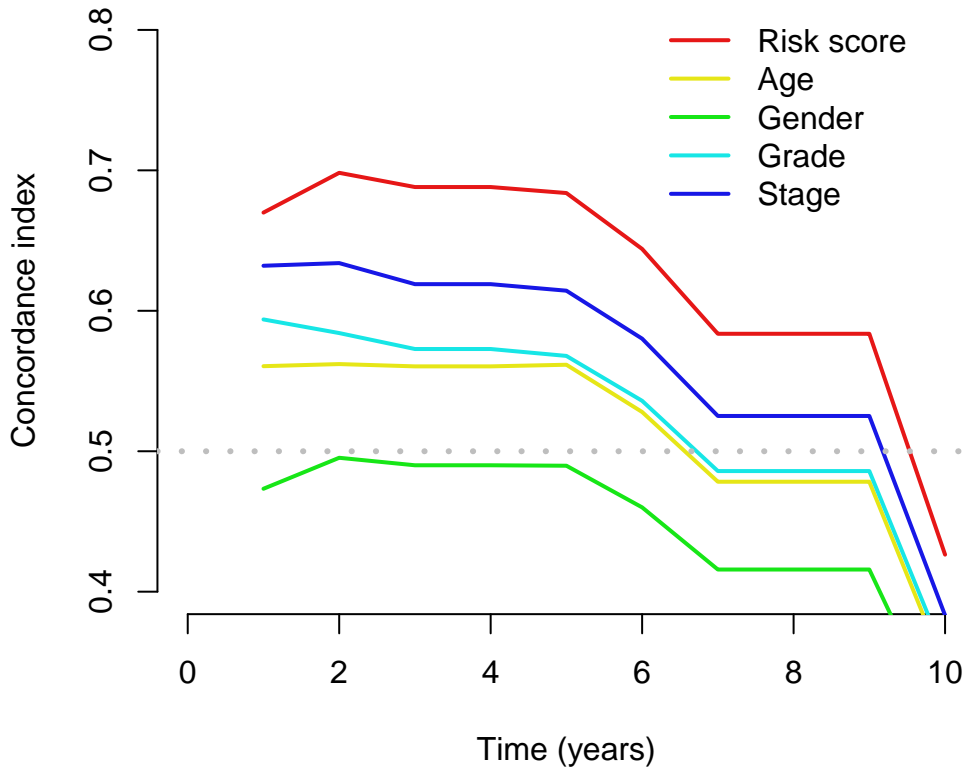

Supplement: Supplementary file 3 [file DataSheet2.zip › Raw Data2/C-index/C-index.pdf]

# Patients with Stage I-II

Risk + high + low

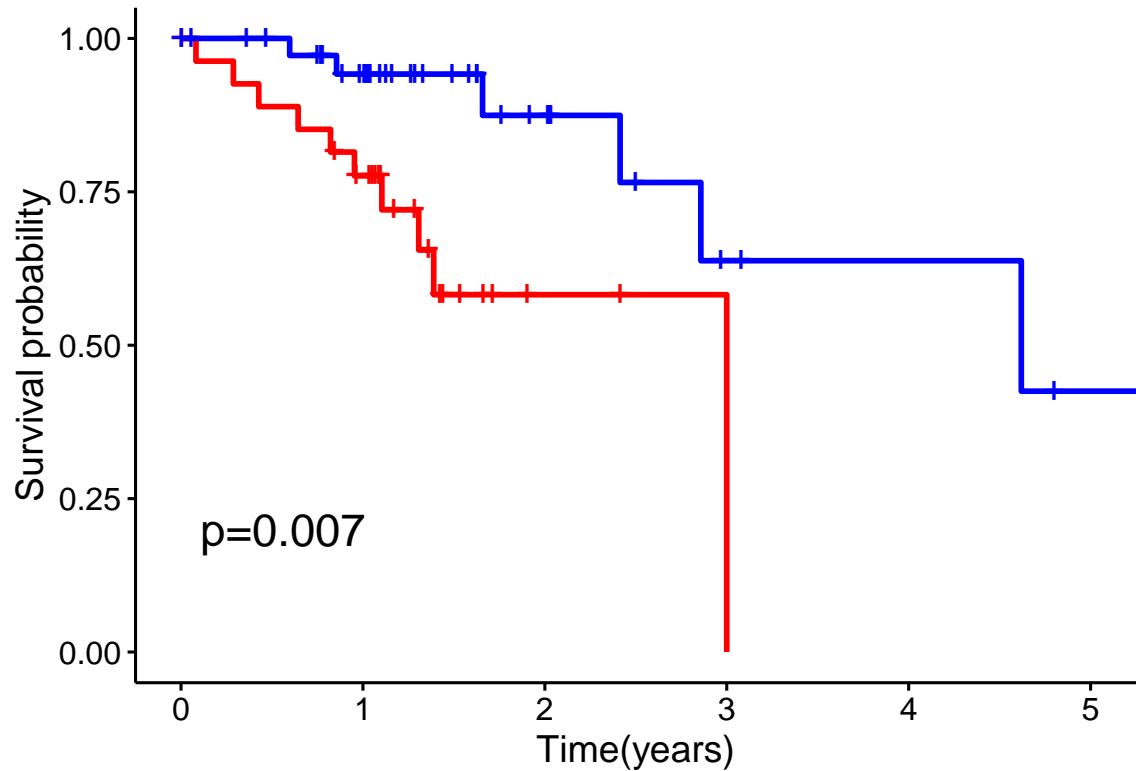

Supplement: Supplementary file 3 [file DataSheet2.zip › Raw Data2/cliGroupSur/survival.Stage_Stage I-II.pdf]

# Patients with Stage III–IV

Risk + high + low

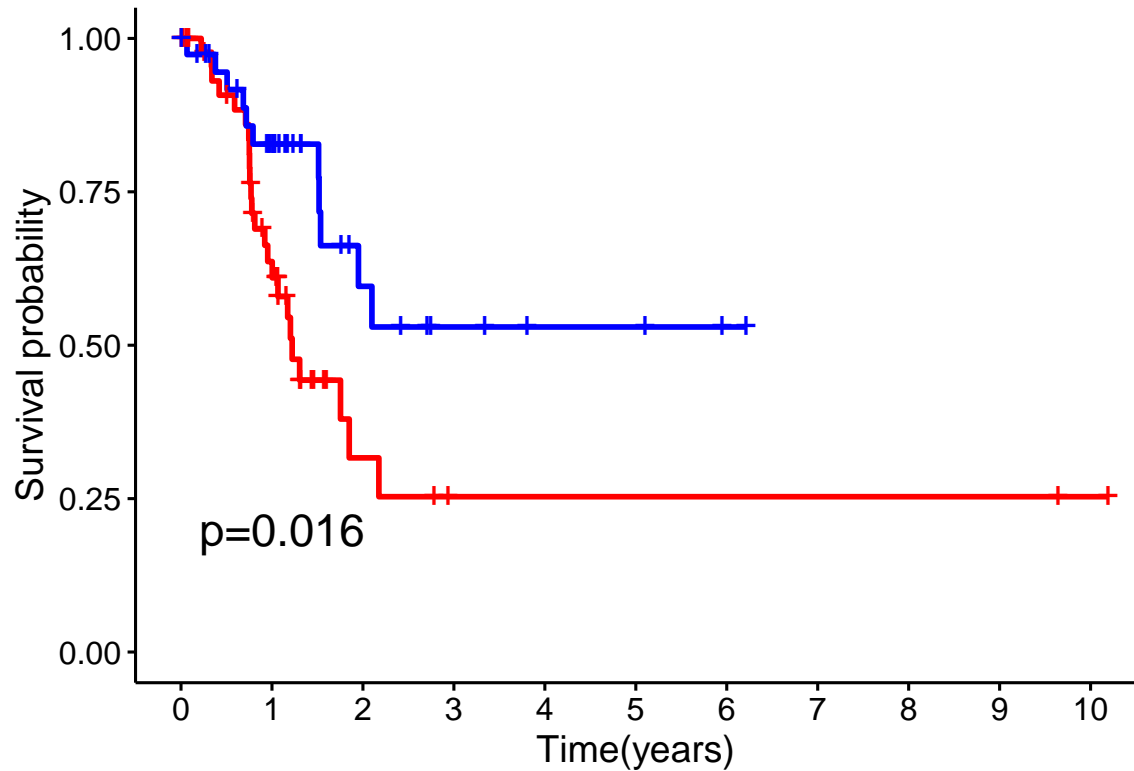

Supplement: Supplementary file 3 [file DataSheet2.zip › Raw Data2/cliGroupSur/survival.Stage_Stage III-IV.pdf]

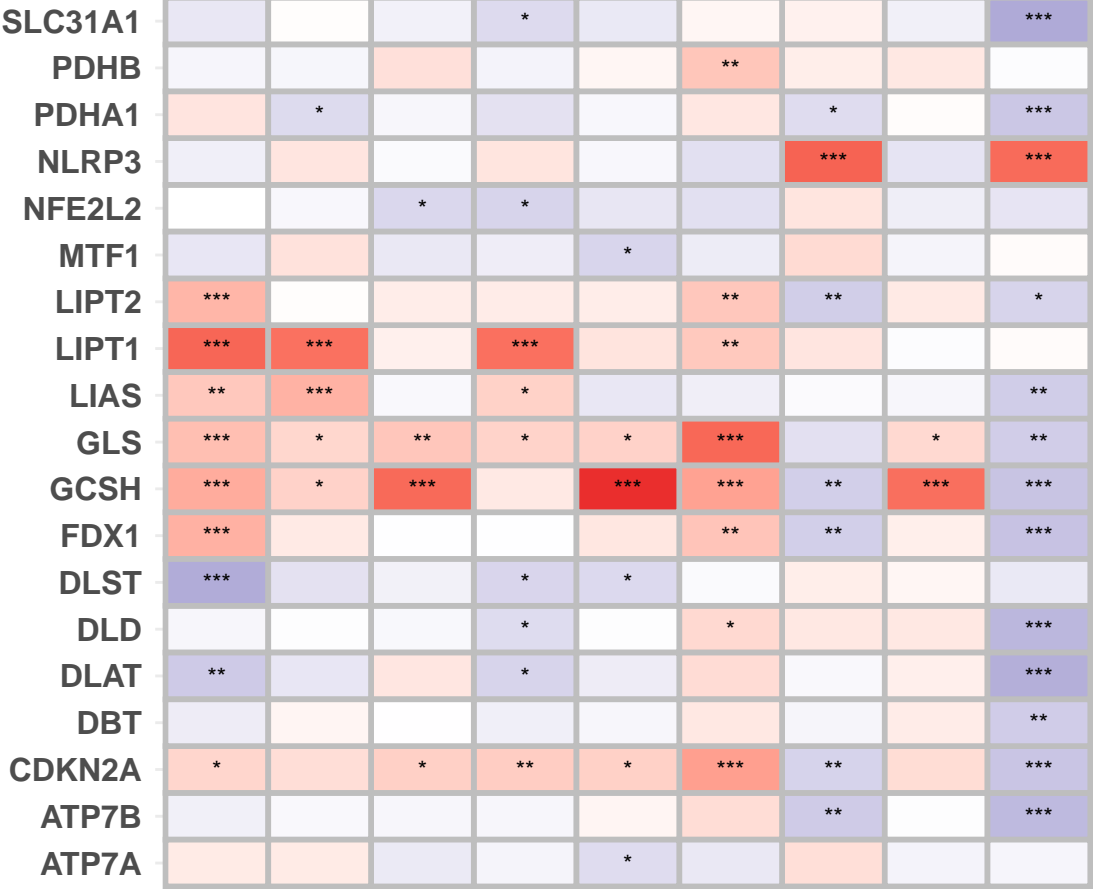

\*\*\* p<0.001  
\*\* p<0.01  
\* p<0.05

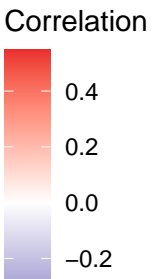

Supplement: Supplementary file 3 [file DataSheet2.zip › Raw Data2/corplot/cor.pdf]

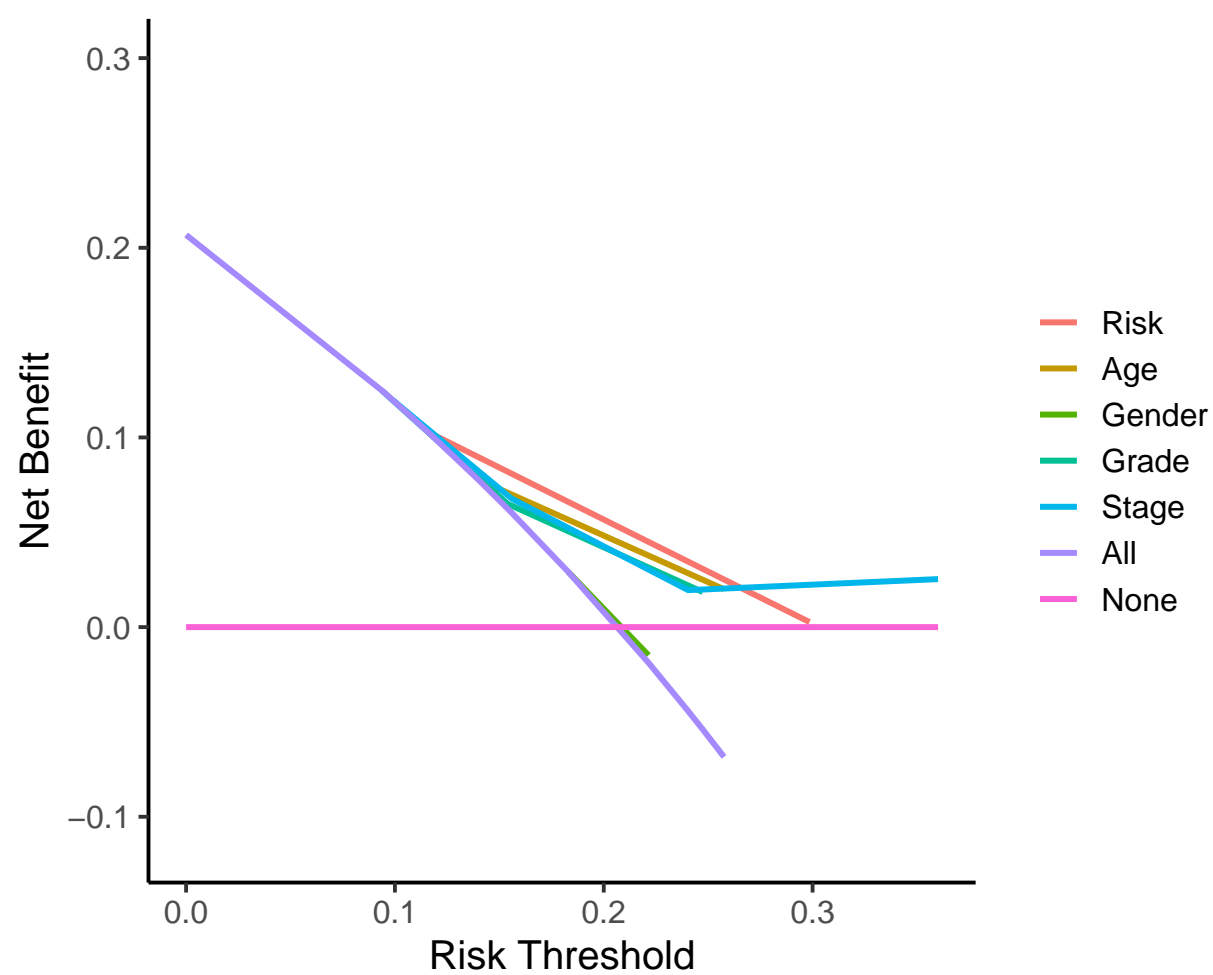

Supplement: Supplementary file 3 [file DataSheet2.zip › Raw Data2/DCA/DCA.pdf]

Cuproptosis

lncRNA

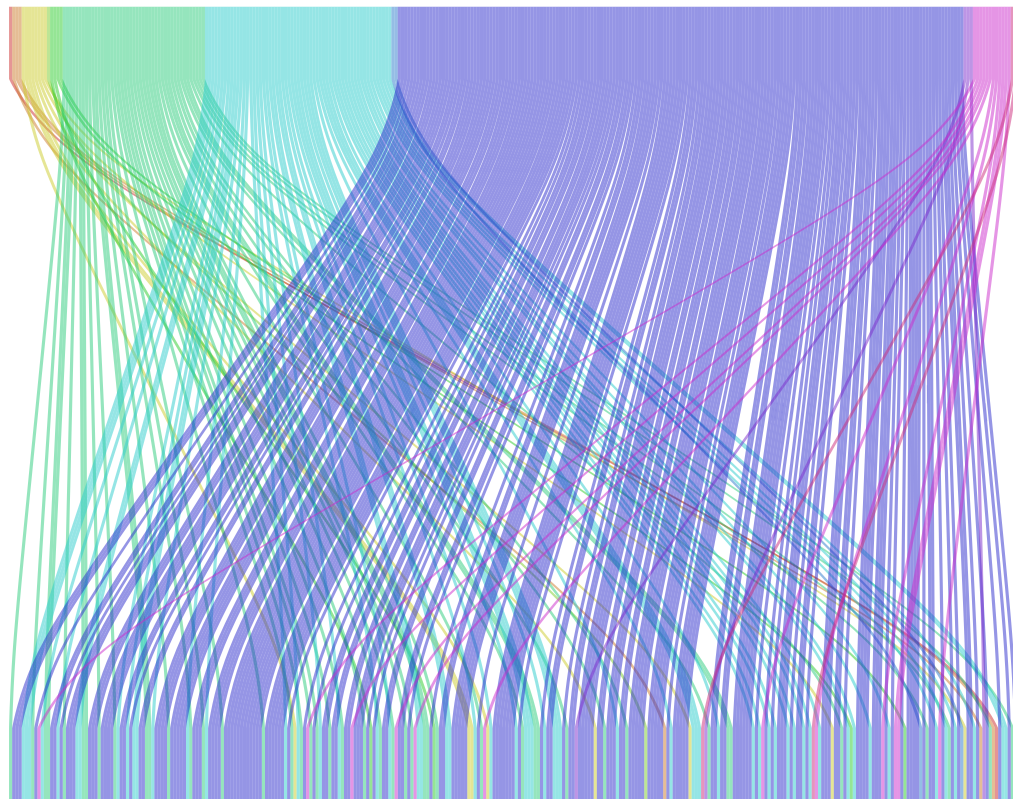

Cuproptosis

- ATP7A
- ATP7B
- CDKN2A
- DBT
- FDX1
- GCSH
- GLS
- LIAS
- LIPT1
- LIPT2
- NLRP3
- SLC31A1

Supplement: Supplementary file 3 [file DataSheet2.zip › Raw Data2/ggalluvial/ggalluvial.pdf]

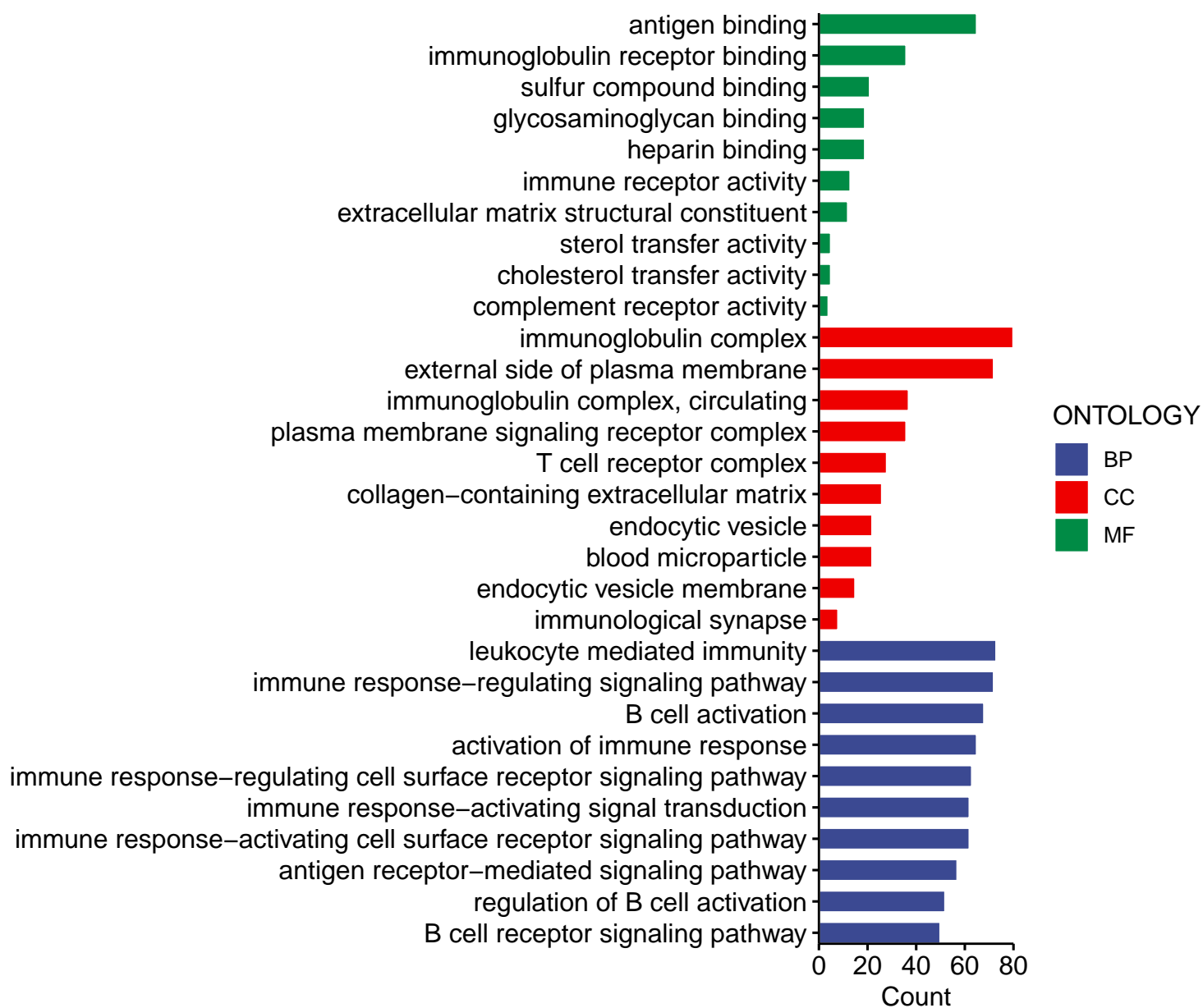

Supplement: Supplementary file 3 [file DataSheet2.zip › Raw Data2/GO/barplot.color.pdf]

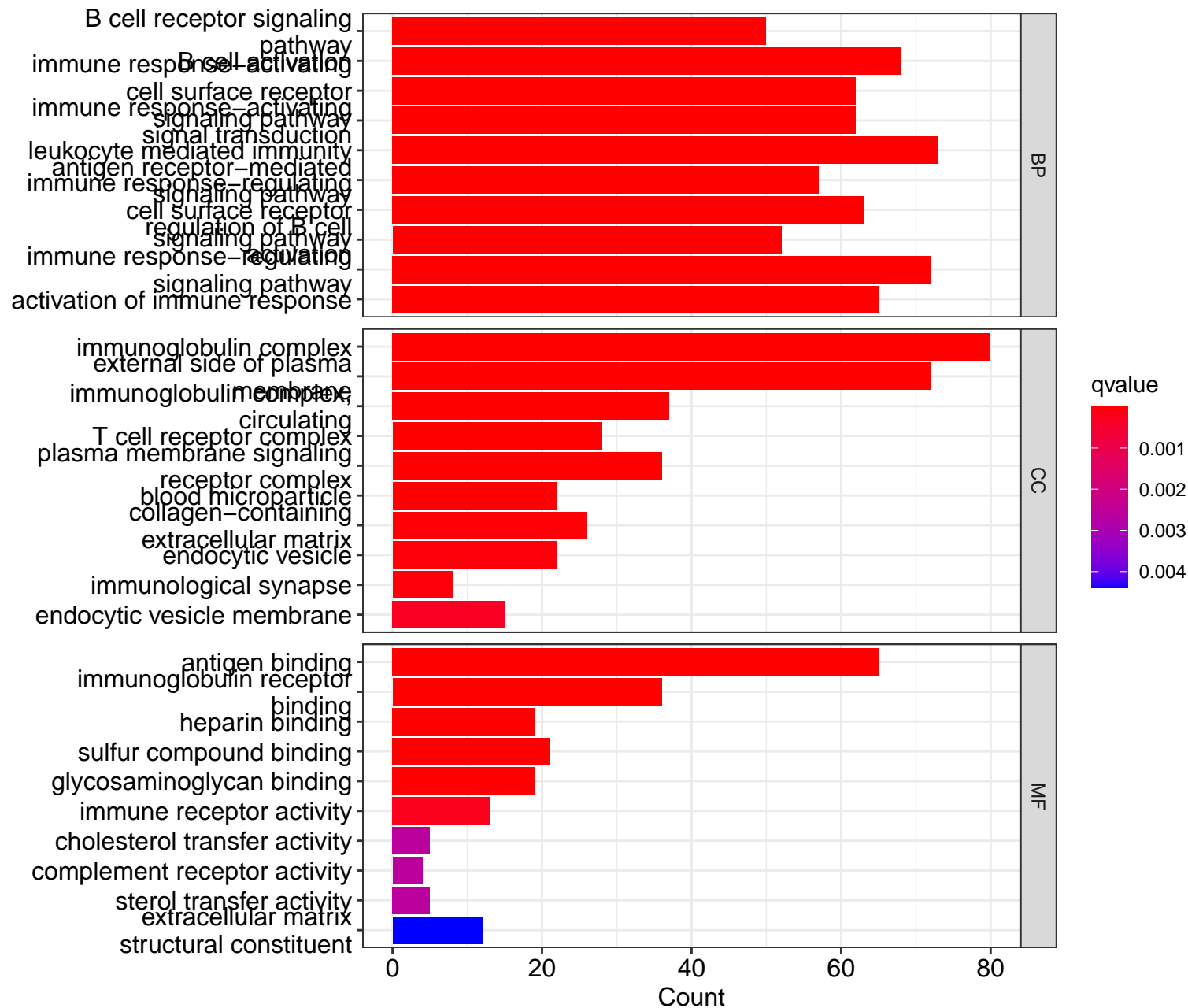

Supplement: Supplementary file 3 [file DataSheet2.zip › Raw Data2/GO/barplot.pdf]

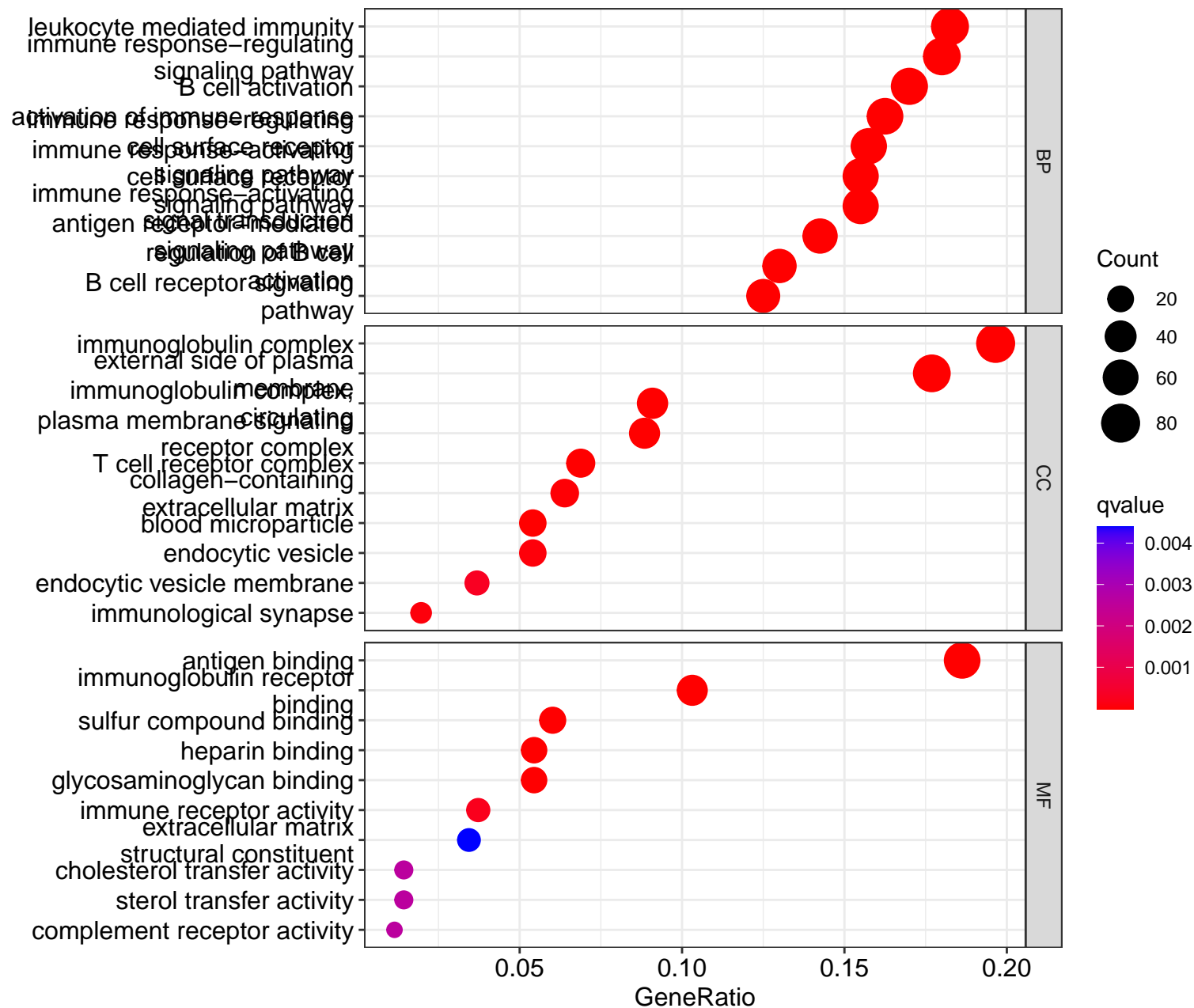

Supplement: Supplementary file 3 [file DataSheet2.zip › Raw Data2/GO/bubble.pdf]

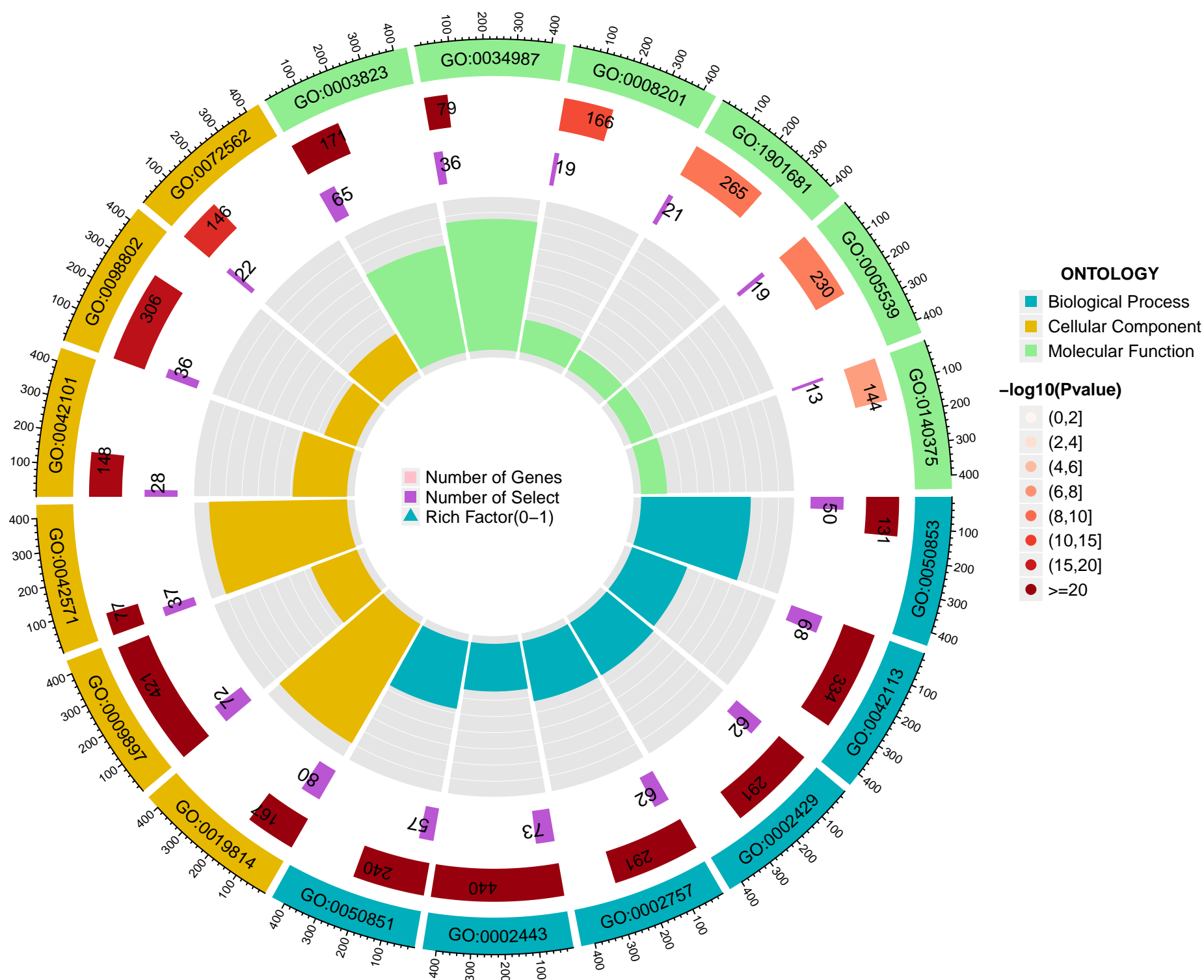

Supplement: Supplementary file 3 [file DataSheet2.zip › Raw Data2/GO/GO.circlize.pdf]

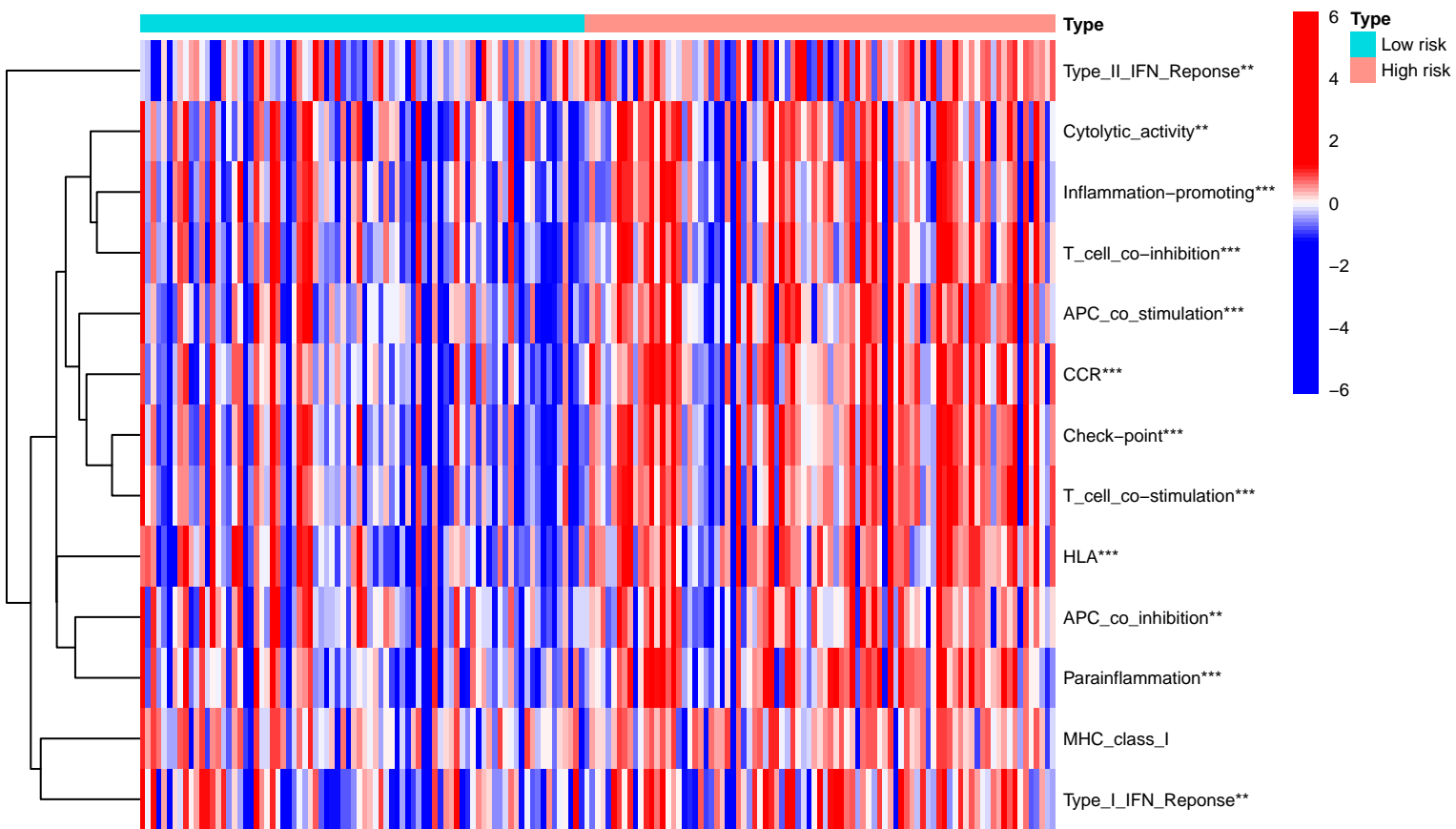

Supplement: Supplementary file 3 [file DataSheet2.zip › Raw Data2/immFunction/immFunctionheatmap.pdf]

|           | pvalue | Hazard ratio       |
|-----------|--------|--------------------|
| Age       | 0.001  | 1.050(1.019–1.082) |
| Gender    | 0.978  | 1.009(0.548–1.856) |
| Grade     | 0.297  | 1.366(0.760–2.455) |
| Stage     | <0.001 | 2.079(1.382–3.128) |
| riskScore | <0.001 | 1.092(1.045–1.142) |

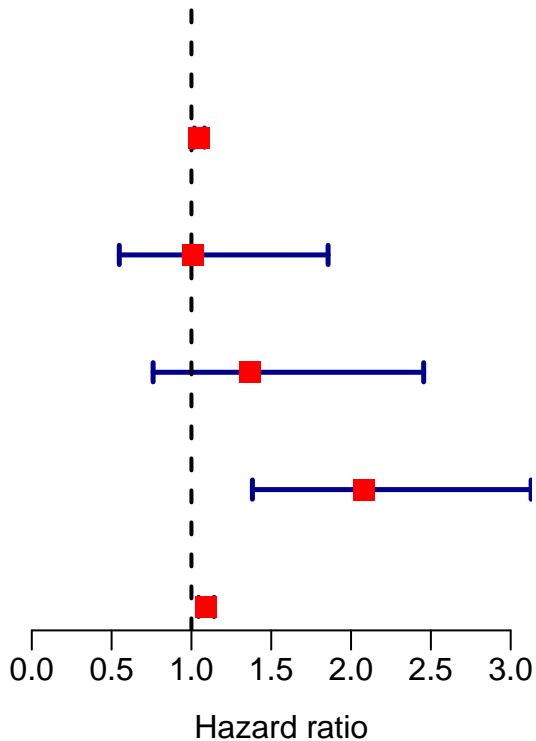

Supplement: Supplementary file 3 [file DataSheet2.zip › Raw Data2/indep/all.multiCox.pdf]

|           | pvalue | Hazard ratio       |
|-----------|--------|--------------------|
| Age       | 0.062  | 1.027(0.999–1.056) |
| Gender    | 0.473  | 1.239(0.690–2.227) |
| Grade     | 0.063  | 1.701(0.971–2.981) |
| Stage     | 0.006  | 1.623(1.146–2.299) |
| riskScore | <0.001 | 1.072(1.031–1.114) |

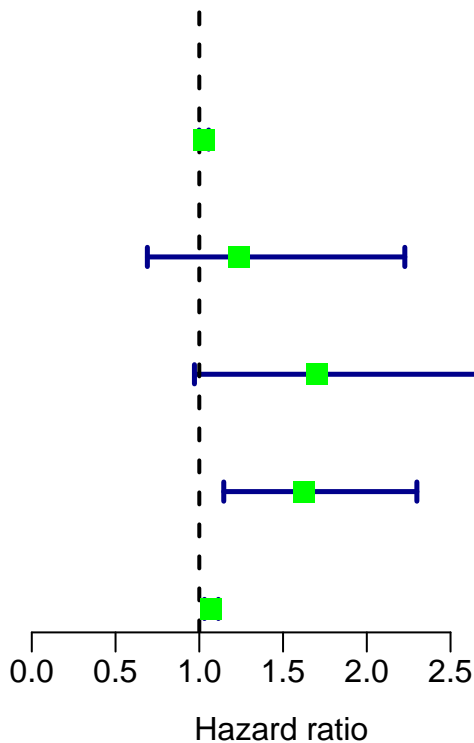

Supplement: Supplementary file 3 [file DataSheet2.zip › Raw Data2/indep/all.uniCox.pdf]

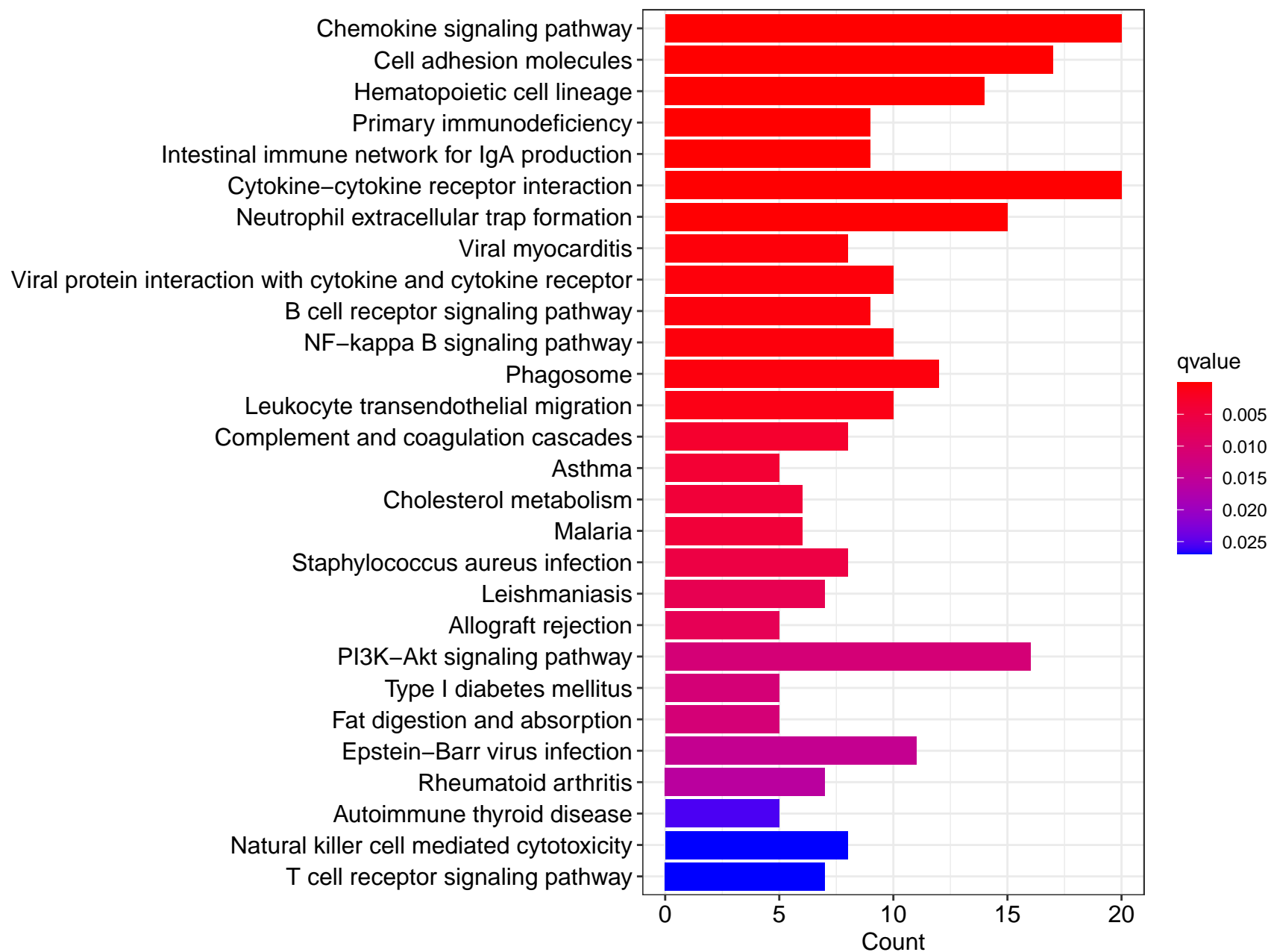

Supplement: Supplementary file 3 [file DataSheet2.zip › Raw Data2/KEGG/barplot.pdf]

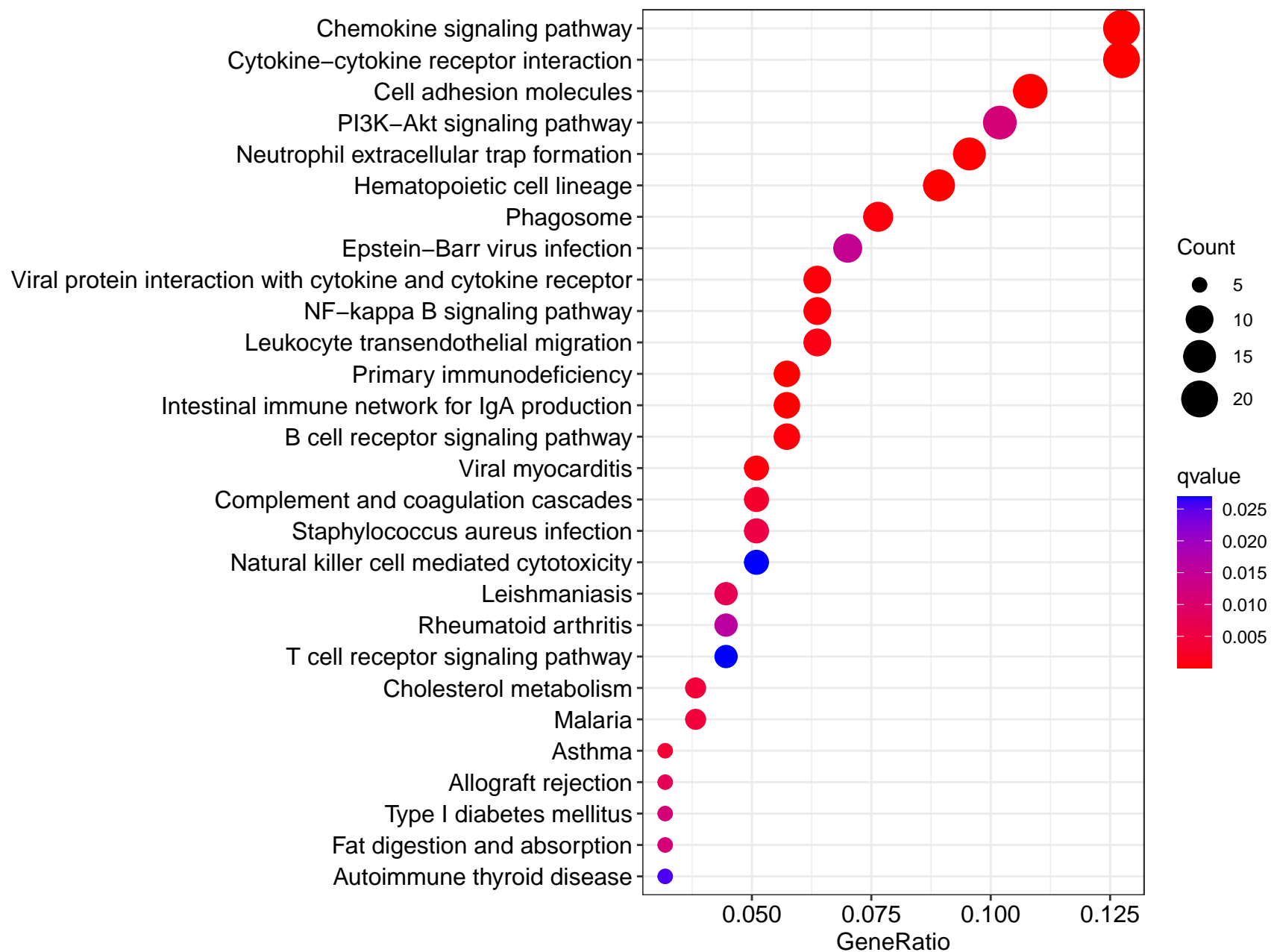

Supplement: Supplementary file 3 [file DataSheet2.zip › Raw Data2/KEGG/bubble.pdf]

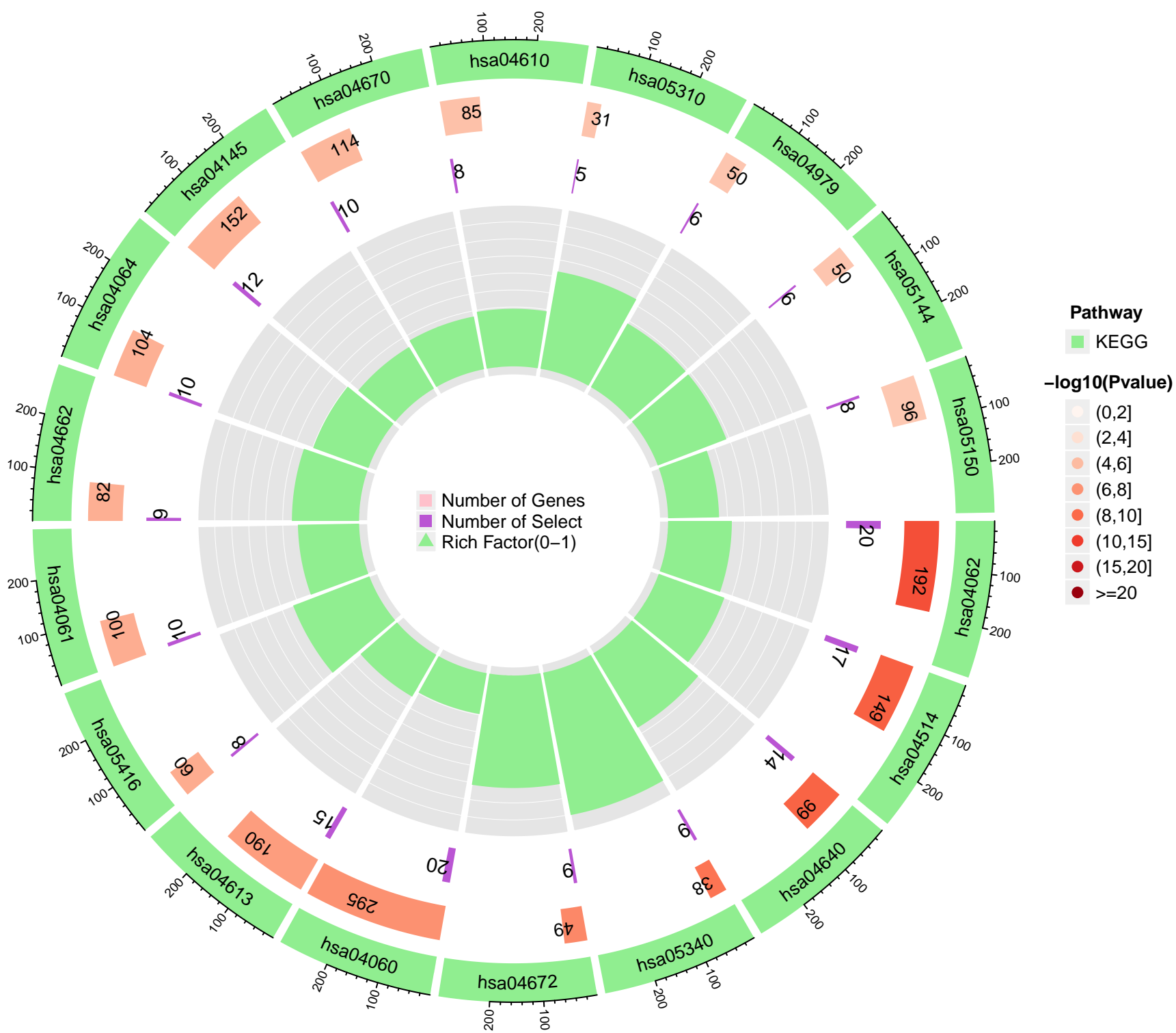

Supplement: Supplementary file 3 [file DataSheet2.zip › Raw Data2/KEGG/KEGG.circlize.pdf]

Altered in 72 (85.71%) of 84 samples.

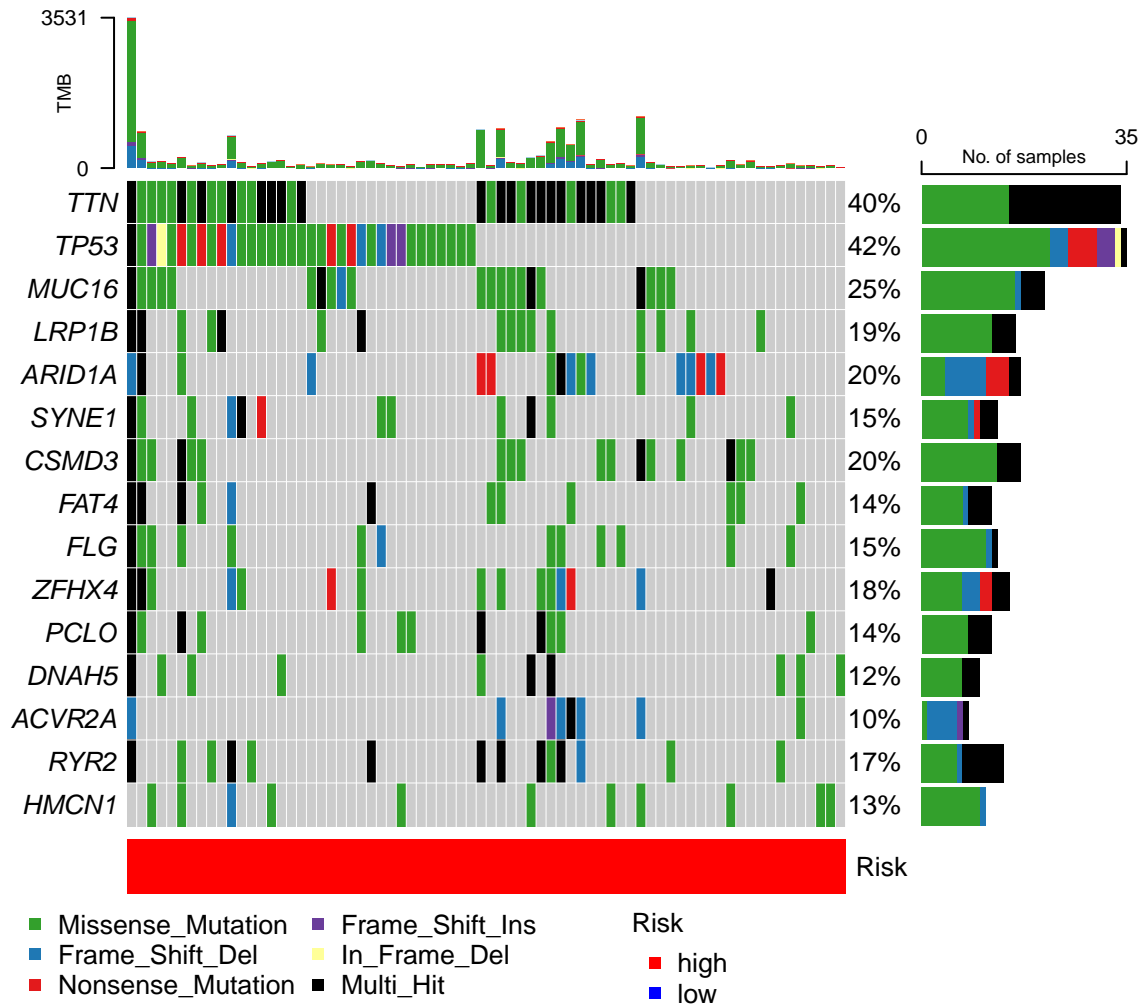

Supplement: Supplementary file 3 [file DataSheet2.zip › Raw Data2/maftools/highmaftools.pdf]

Altered in 73 (92.41%) of 79 samples.

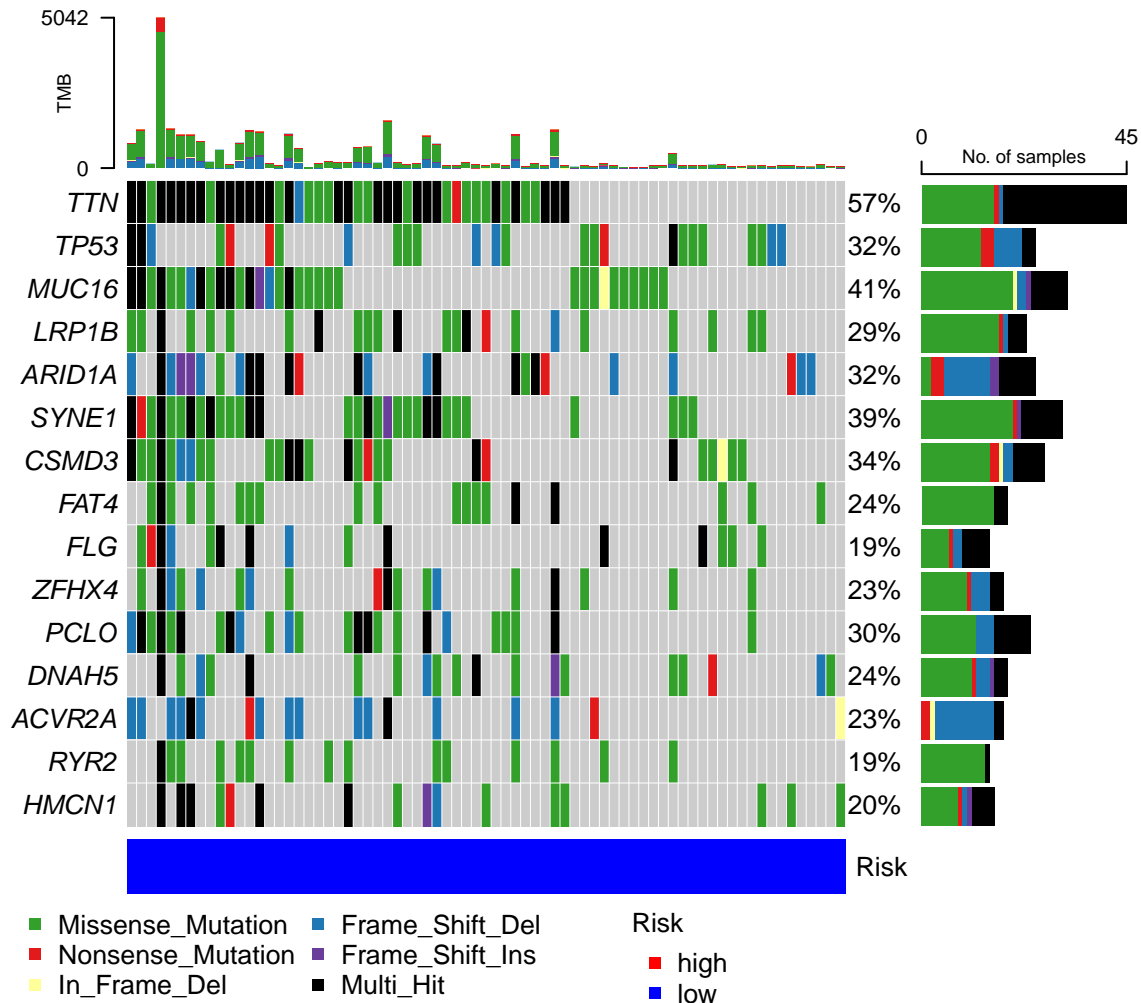

Supplement: Supplementary file 3 [file DataSheet2.zip › Raw Data2/maftools/lowmaftools.pdf]

Partial Likelihood Deviance

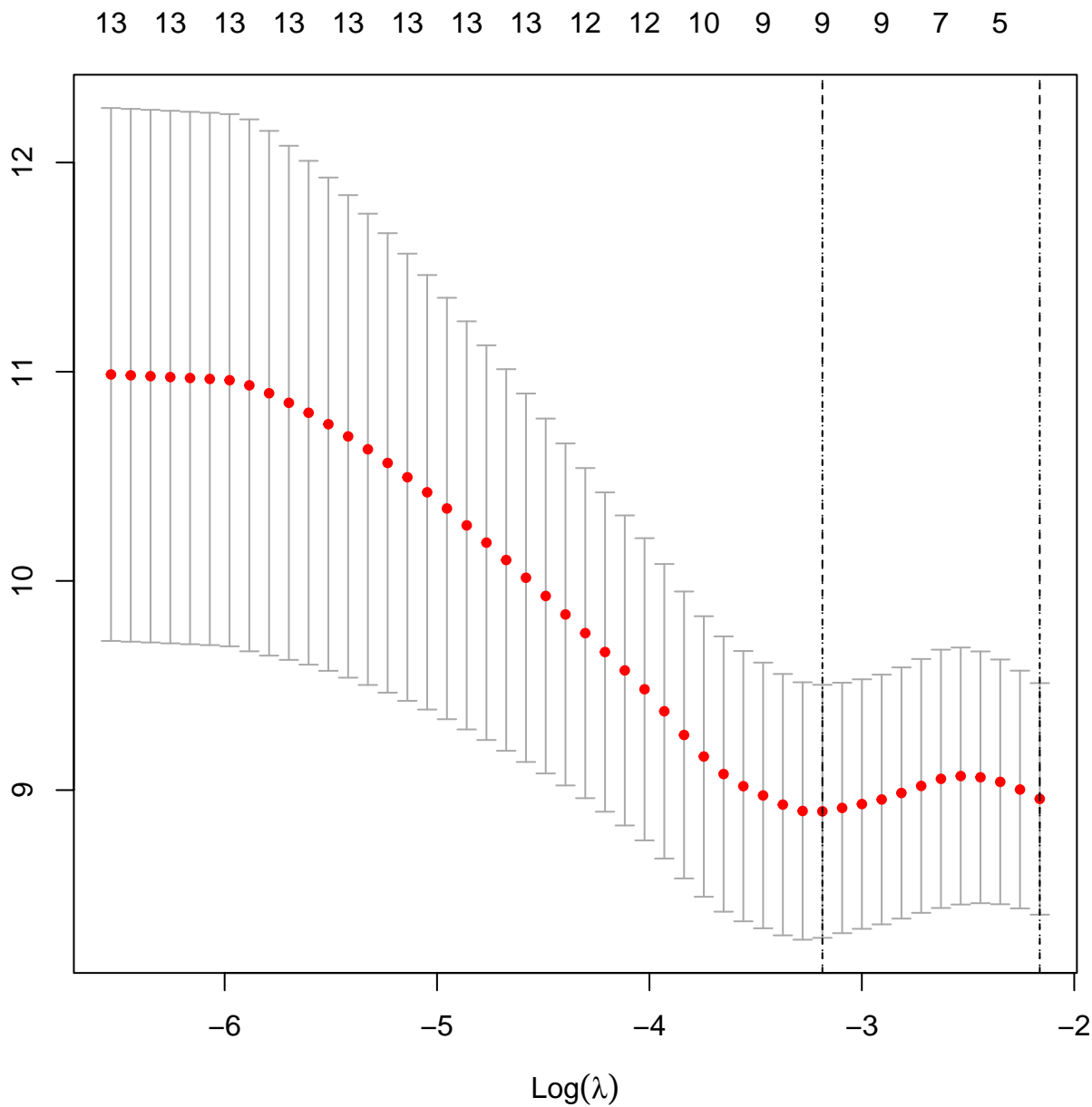

Supplement: Supplementary file 3 [file DataSheet2.zip › Raw Data2/model/cvfit.pdf]

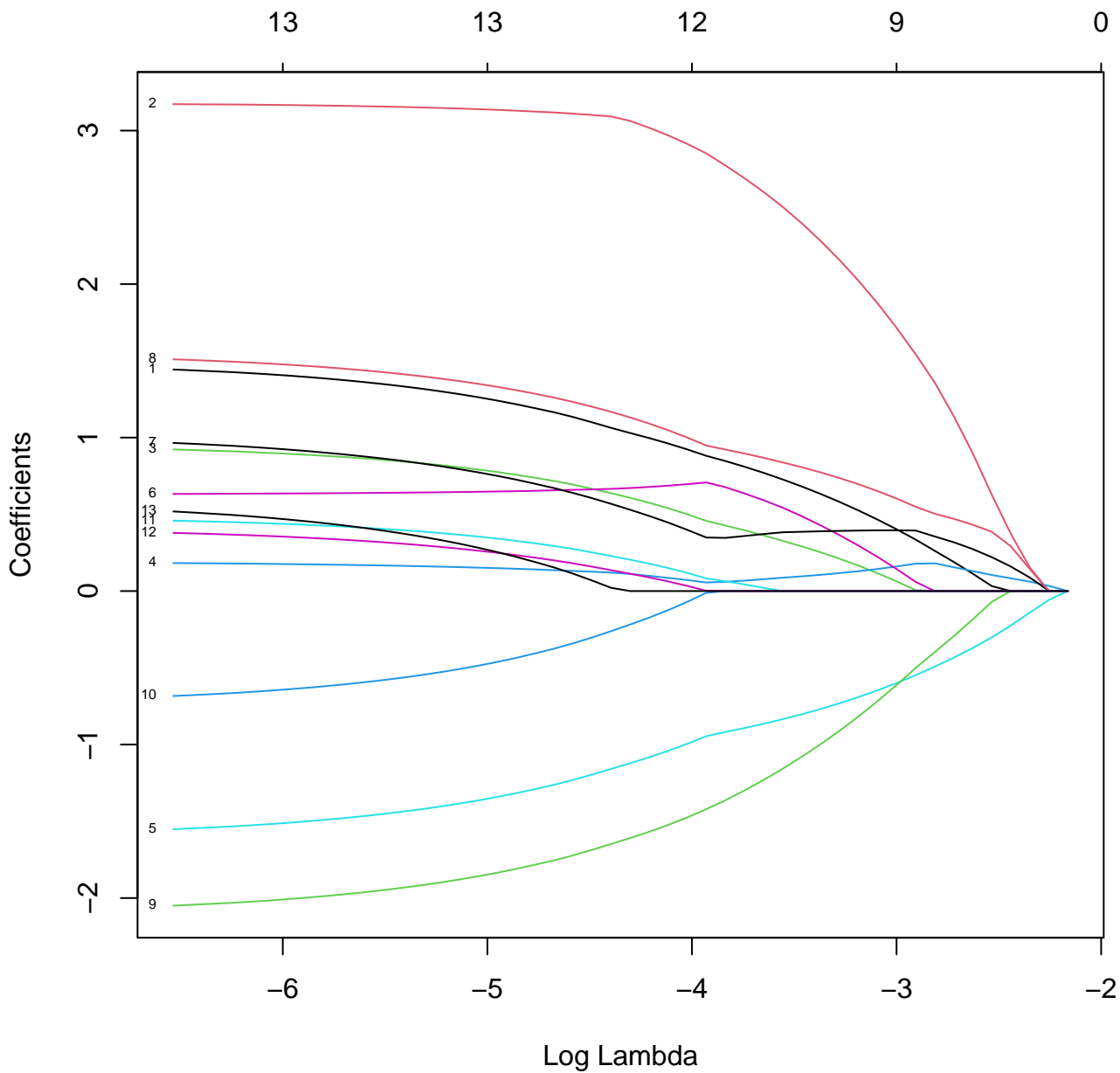

Supplement: Supplementary file 3 [file DataSheet2.zip › Raw Data2/model/lambda.pdf]

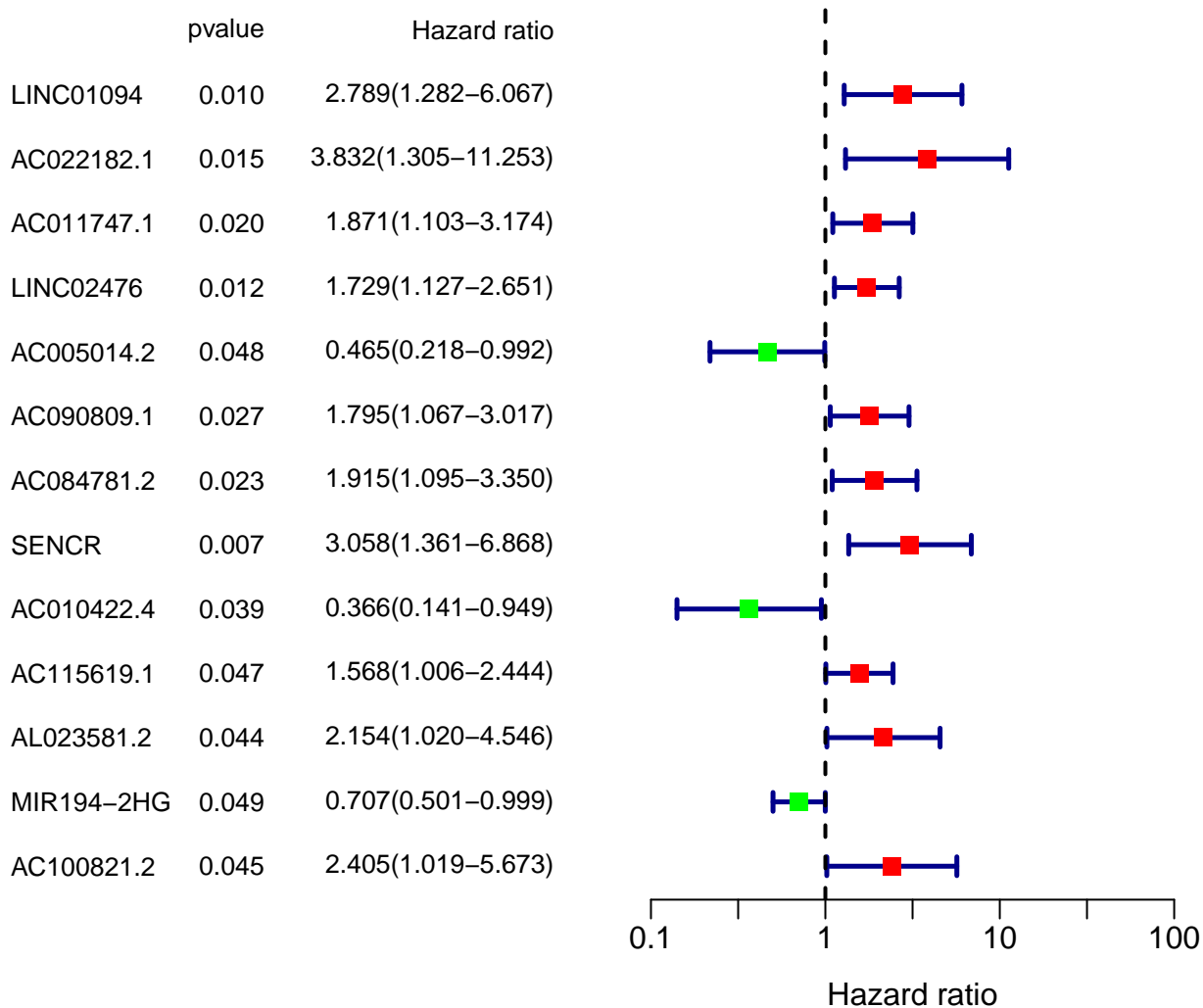

Supplement: Supplementary file 3 [file DataSheet2.zip › Raw Data2/model/uni.foreast.pdf]

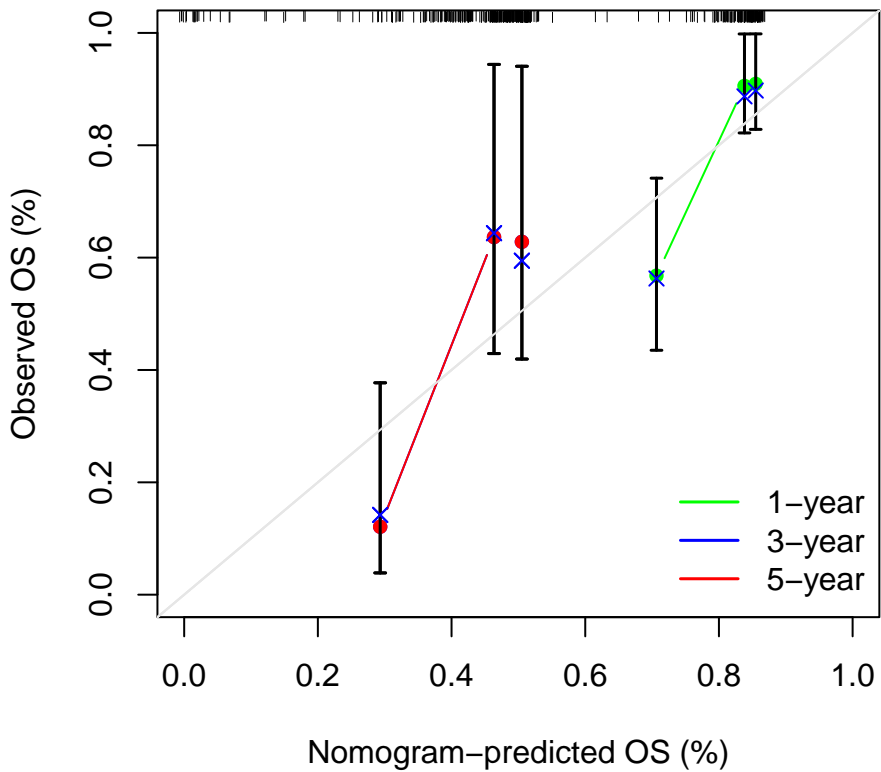

Supplement: Supplementary file 3 [file DataSheet2.zip › Raw Data2/Nomo/calibration.pdf]

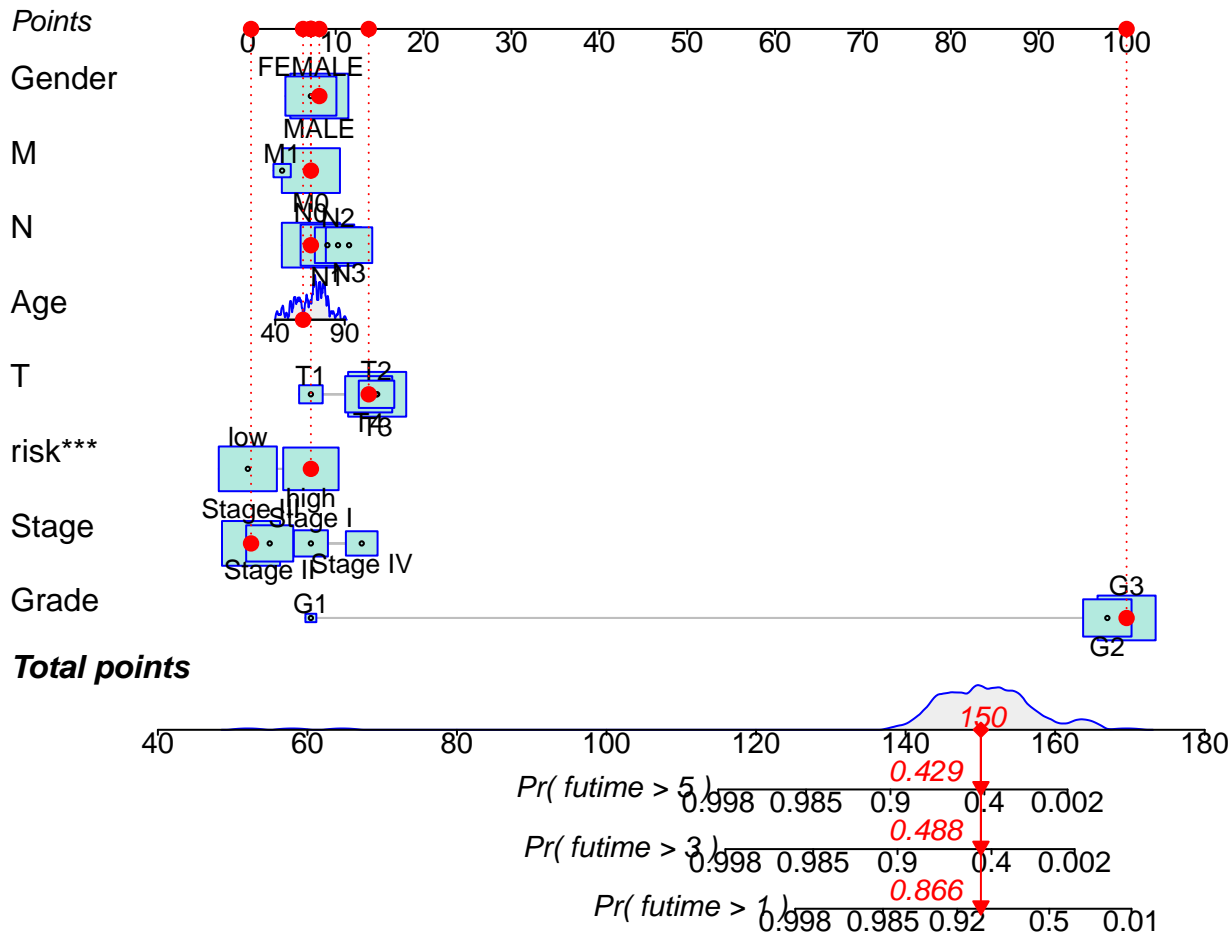

Supplement: Supplementary file 3 [file DataSheet2.zip › Raw Data2/Nomo/Nomo.pdf]

● Low risk ● High risk

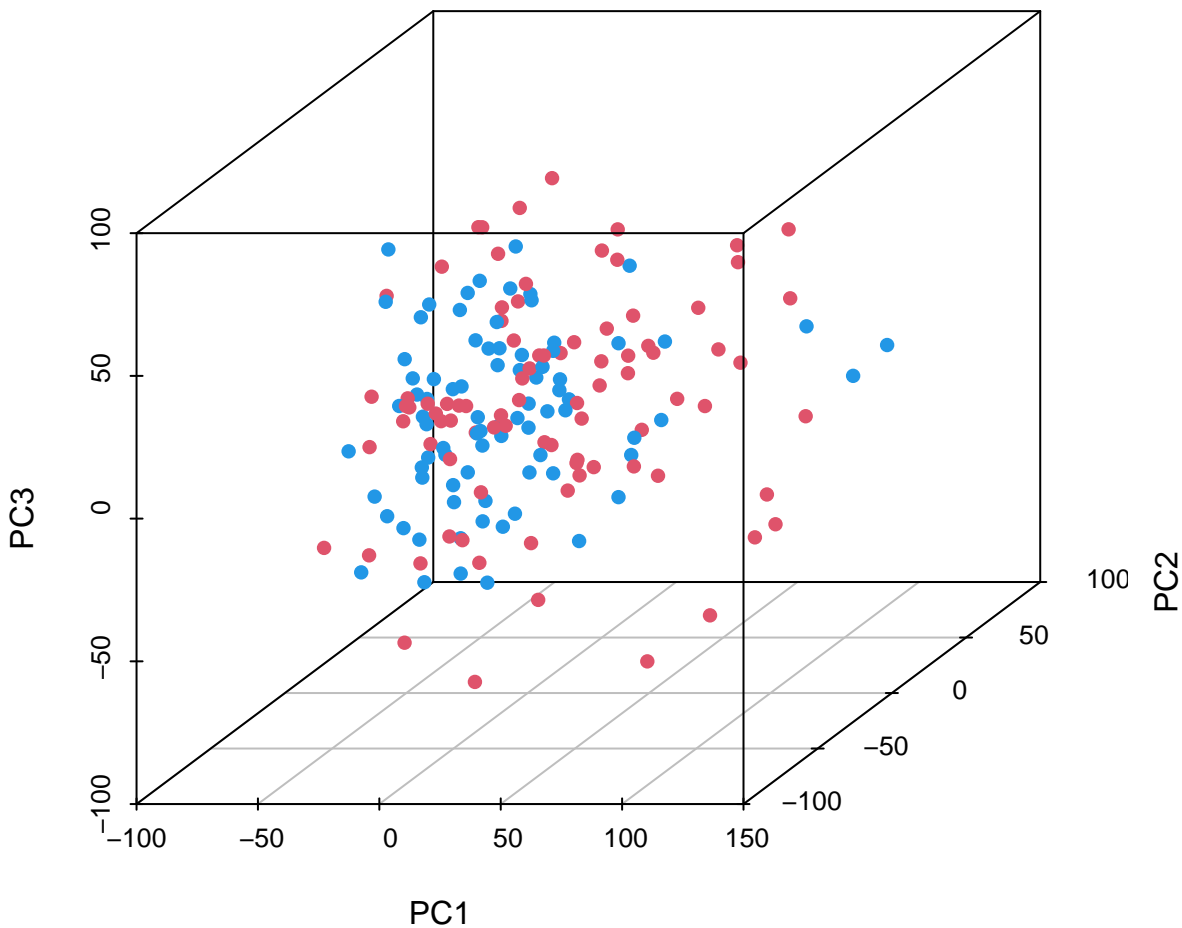

Supplement: Supplementary file 3 [file DataSheet2.zip › Raw Data2/PCA/PCA.allGene.pdf]

● Low risk ● High risk

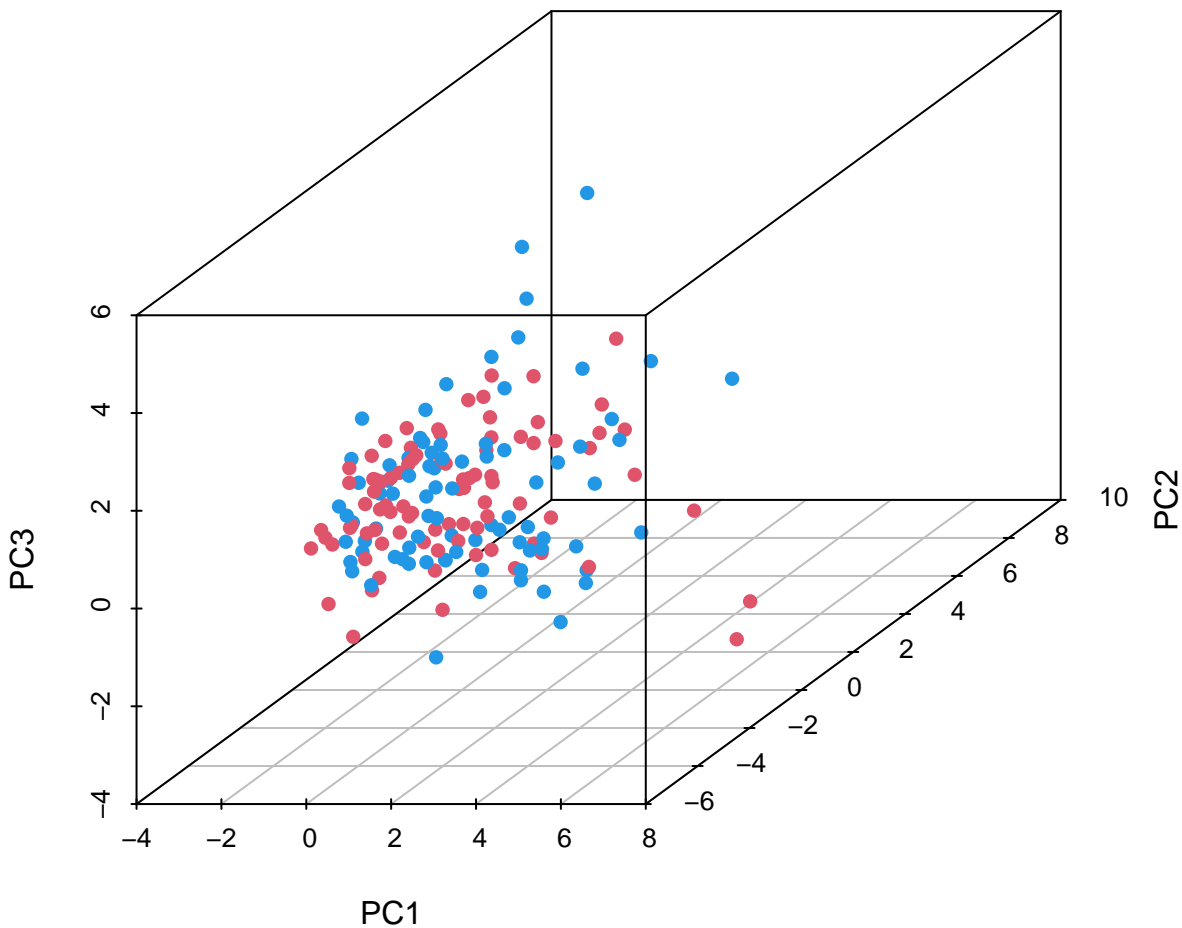

Supplement: Supplementary file 3 [file DataSheet2.zip › Raw Data2/PCA/PCA.cuproptosisGene.pdf]

● Low risk ● High risk

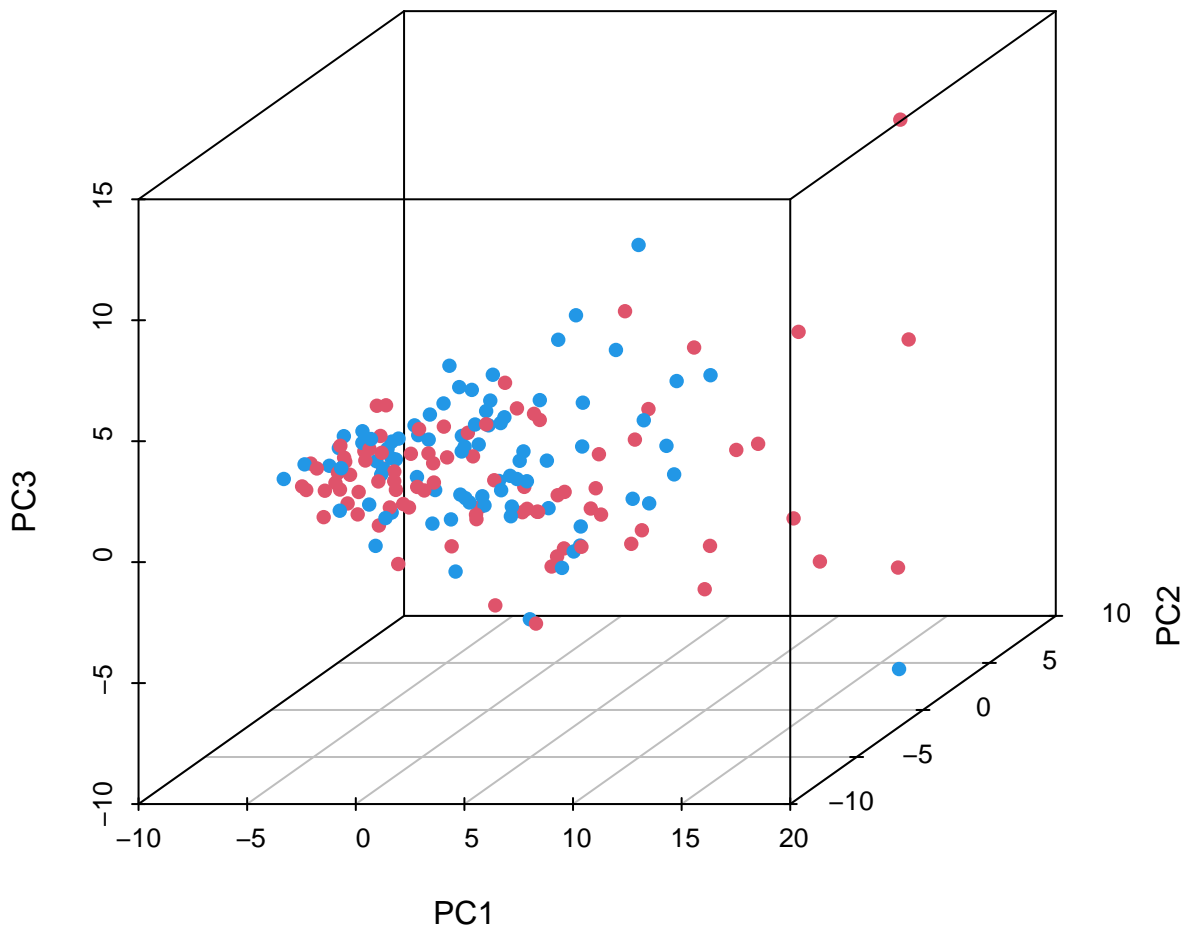

Supplement: Supplementary file 3 [file DataSheet2.zip › Raw Data2/PCA/PCA.cuproptosisLncRNA.pdf]

● Low risk ● High risk

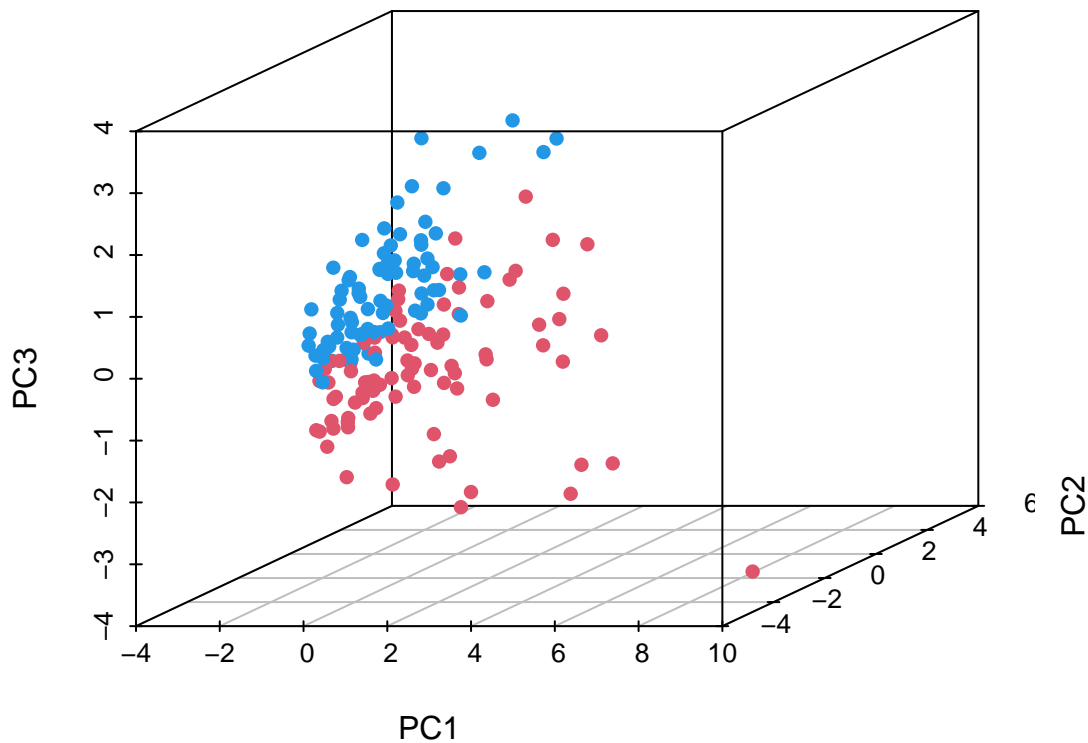

Supplement: Supplementary file 3 [file DataSheet2.zip › Raw Data2/PCA/PCA.riskLnc.pdf]

AZD8055 sensitivity (IC50)

$r = -0.32, p = 3e-05$

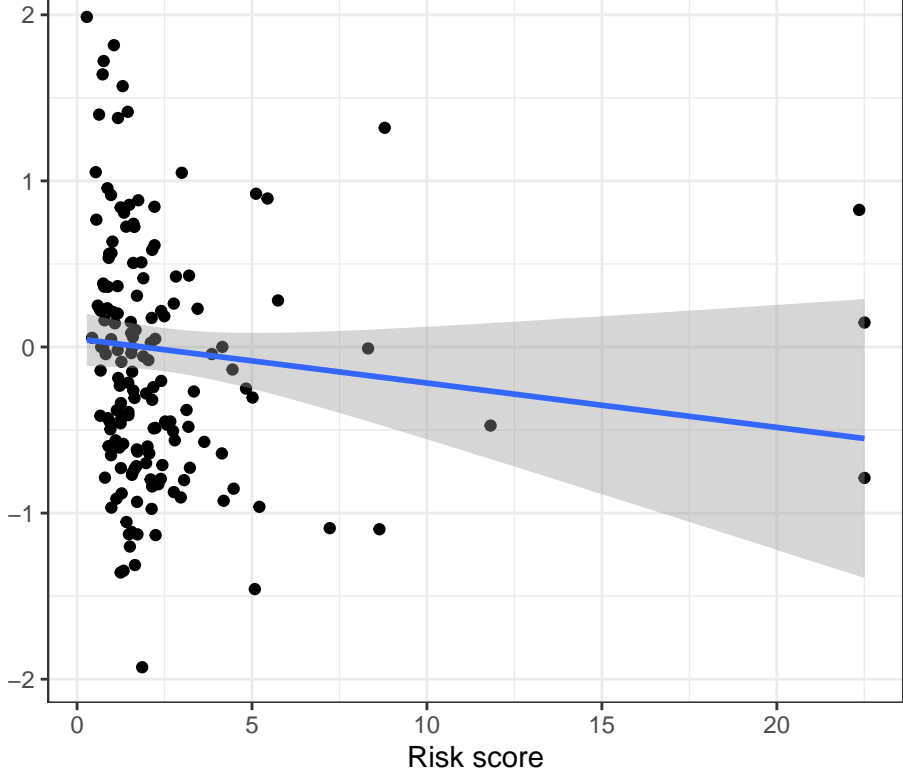

Supplement: Supplementary file 3 [file DataSheet2.zip › Raw Data2/pRRophetic/Cor.AZD8055.pdf]

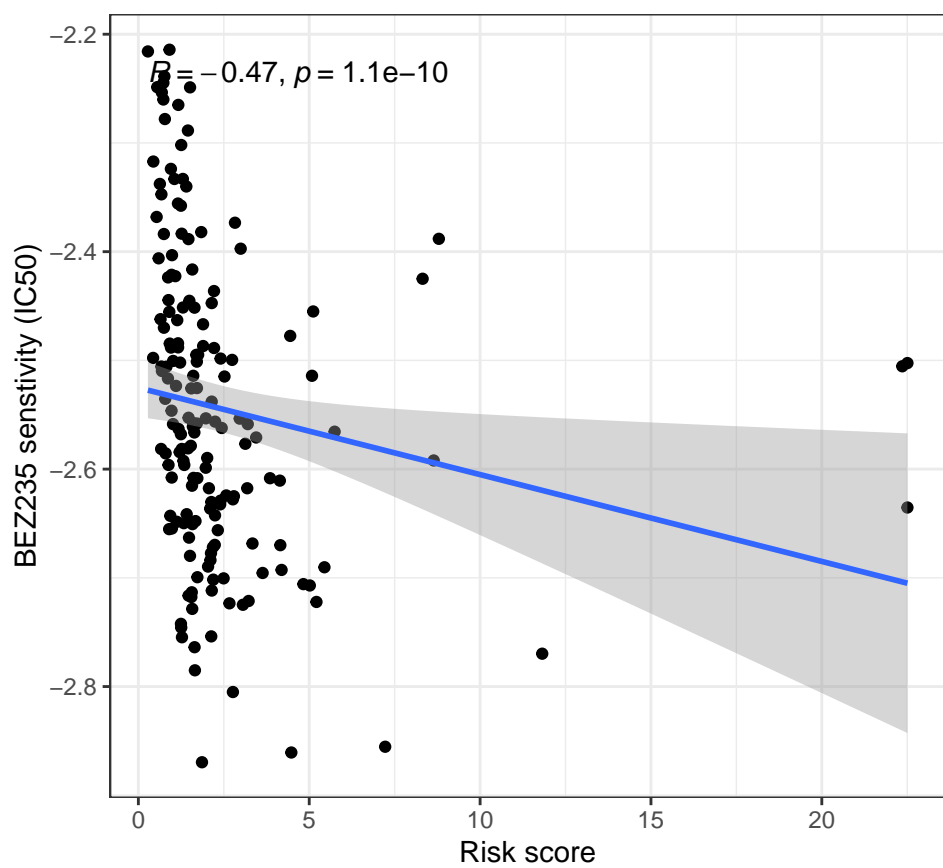

Supplement: Supplementary file 3 [file DataSheet2.zip › Raw Data2/pRRophetic/Cor.BEZ235.pdf]

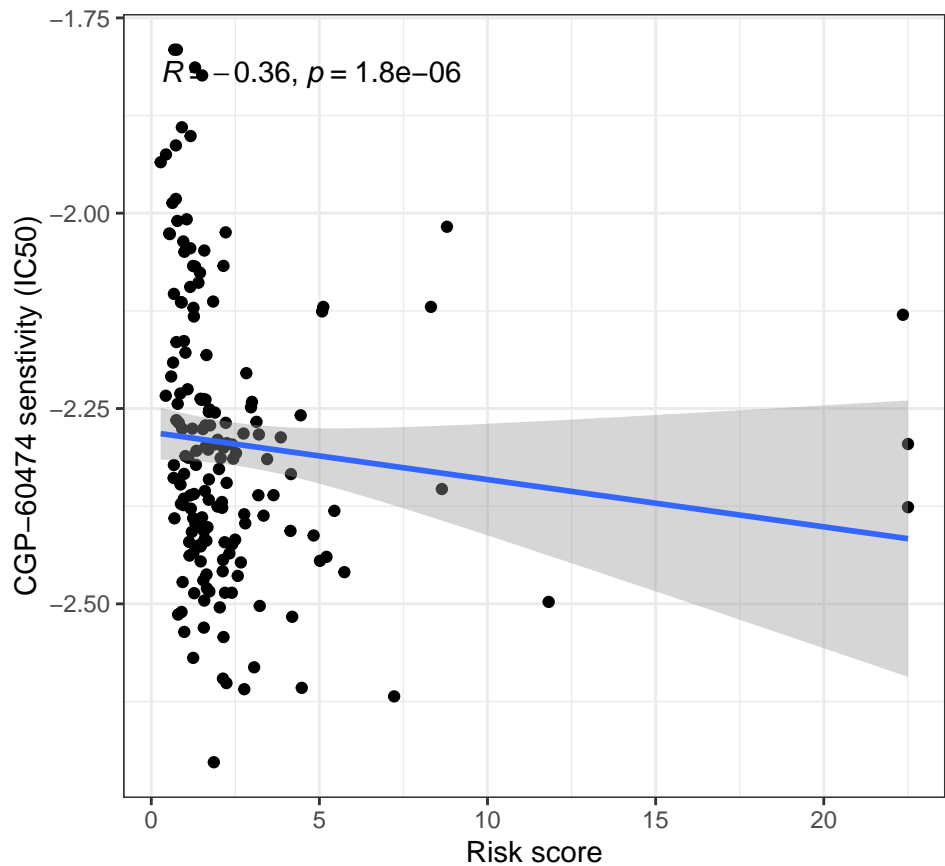

Supplement: Supplementary file 3 [file DataSheet2.zip › Raw Data2/pRRophetic/Cor.CGP-60474.pdf]

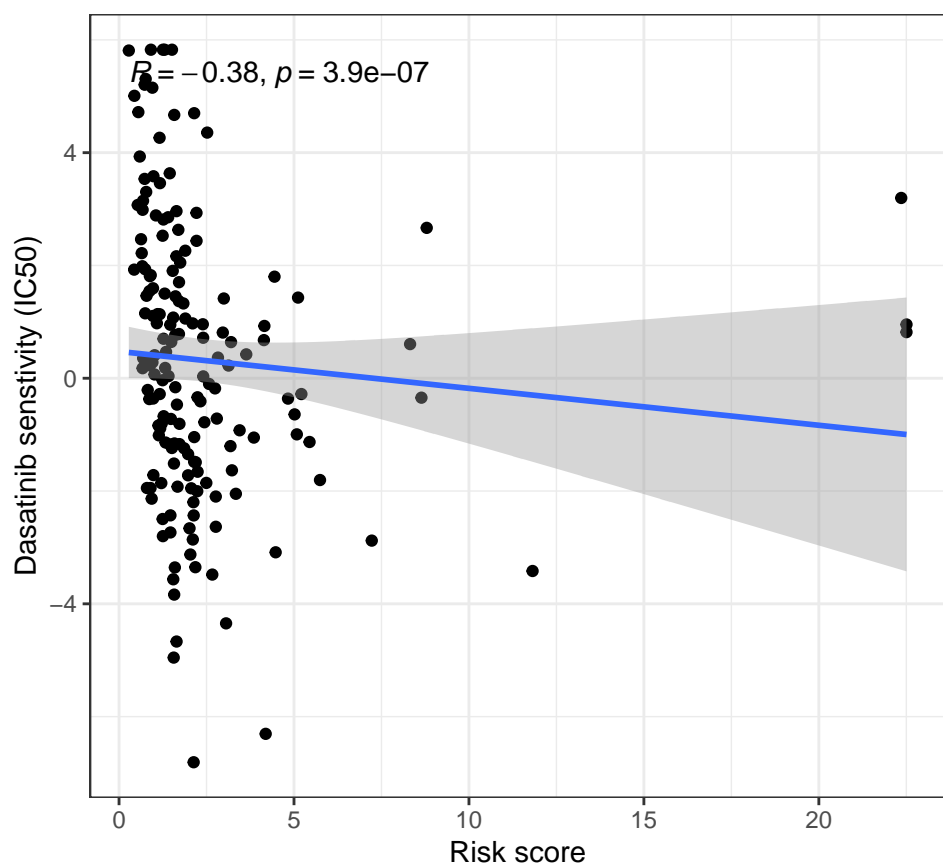

Supplement: Supplementary file 3 [file DataSheet2.zip › Raw Data2/pRRophetic/Cor.Dasatinib.pdf]

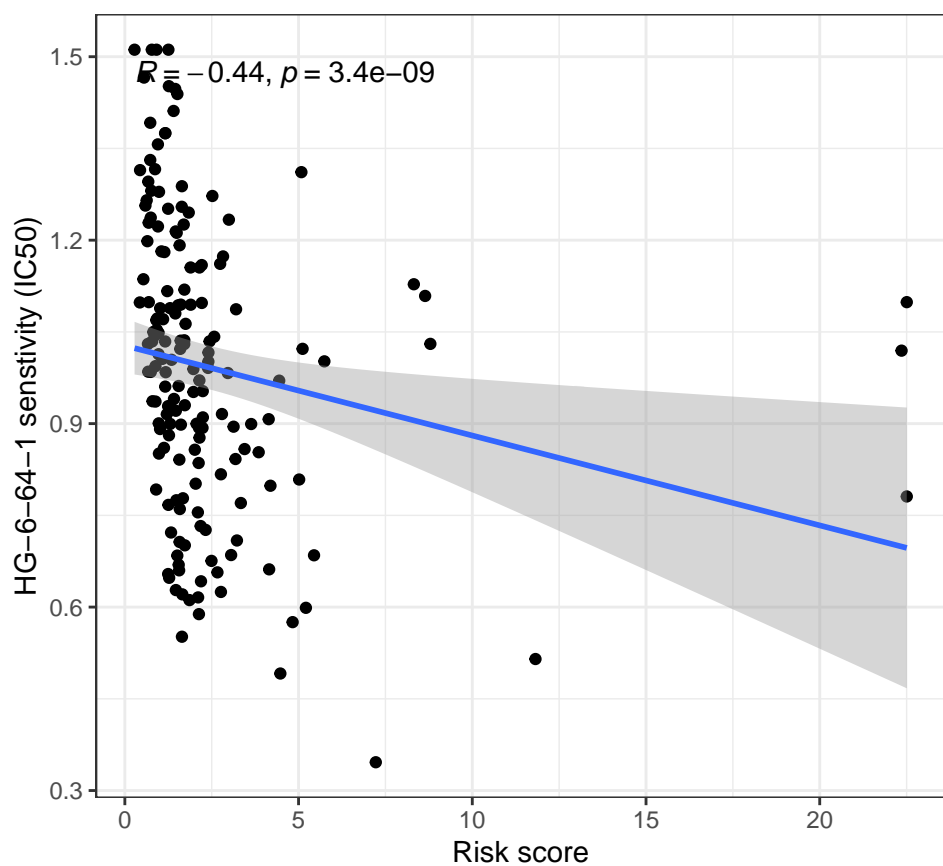

Supplement: Supplementary file 3 [file DataSheet2.zip › Raw Data2/pRRophetic/Cor.HG-6-64-1.pdf]

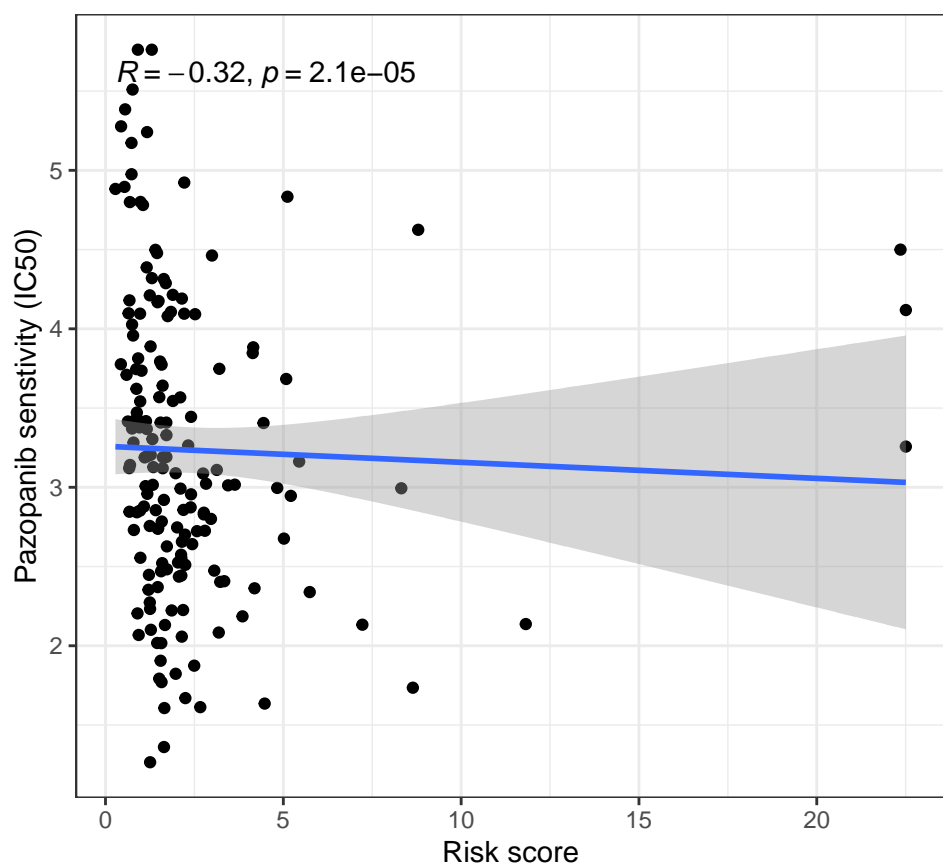

Supplement: Supplementary file 3 [file DataSheet2.zip › Raw Data2/pRRophetic/Cor.Pazopanib.pdf]

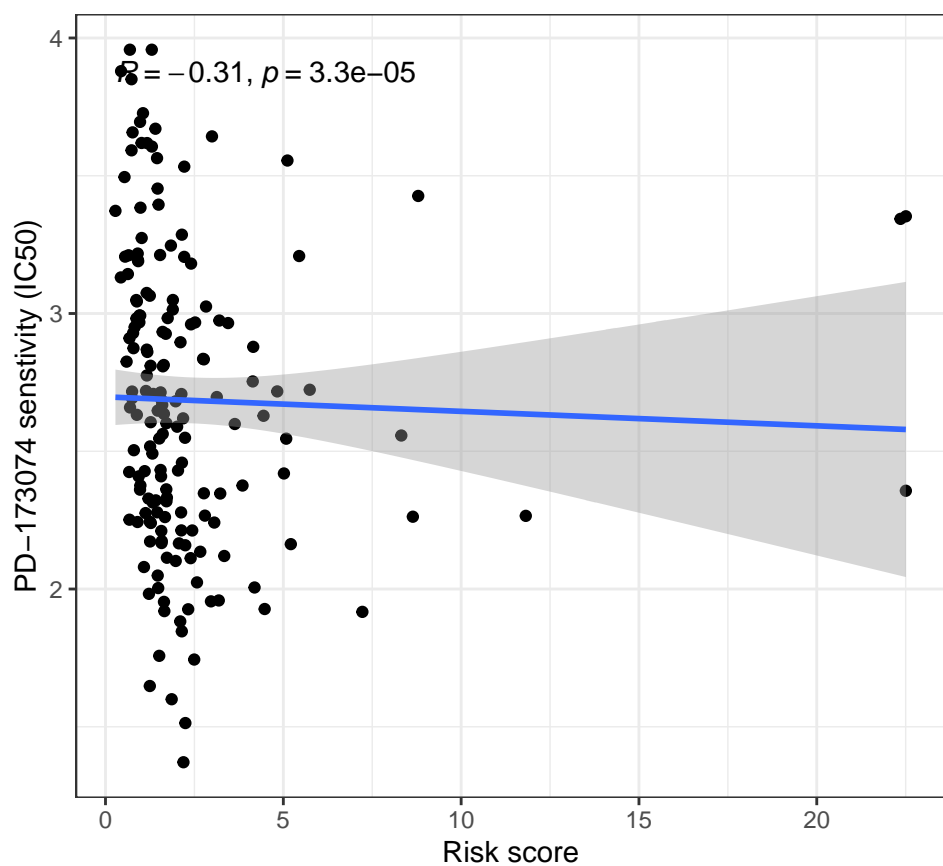

Supplement: Supplementary file 3 [file DataSheet2.zip › Raw Data2/pRRophetic/Cor.PD-173074.pdf]

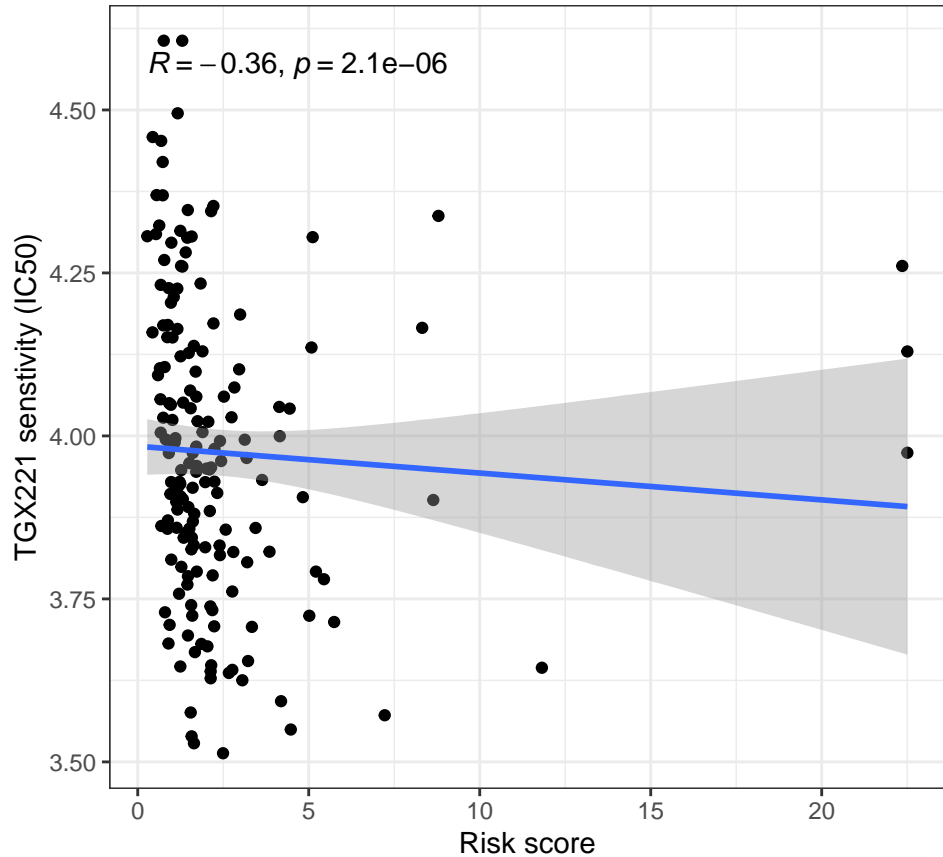

Supplement: Supplementary file 3 [file DataSheet2.zip › Raw Data2/pRRophetic/Cor.TGX221.pdf]

Risk 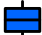 low 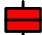 high

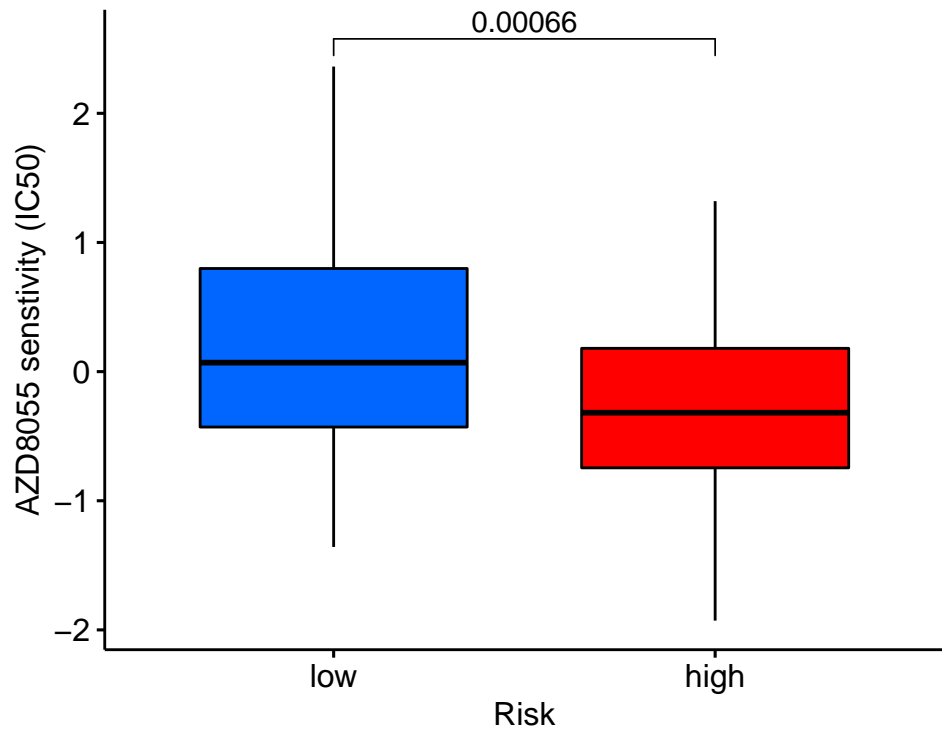

Supplement: Supplementary file 3 [file DataSheet2.zip › Raw Data2/pRRophetic/durgSenstivity.AZD8055.pdf]

Risk 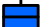 low 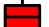 high

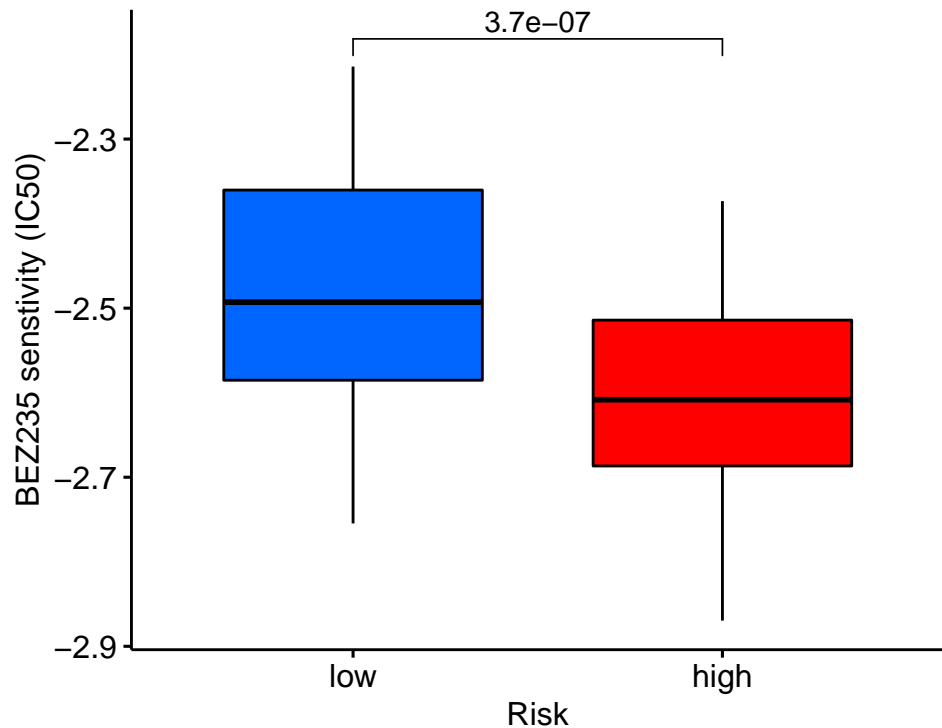

Supplement: Supplementary file 3 [file DataSheet2.zip › Raw Data2/pRRophetic/durgSenstivity.BEZ235.pdf]

Risk 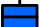 low 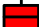 high

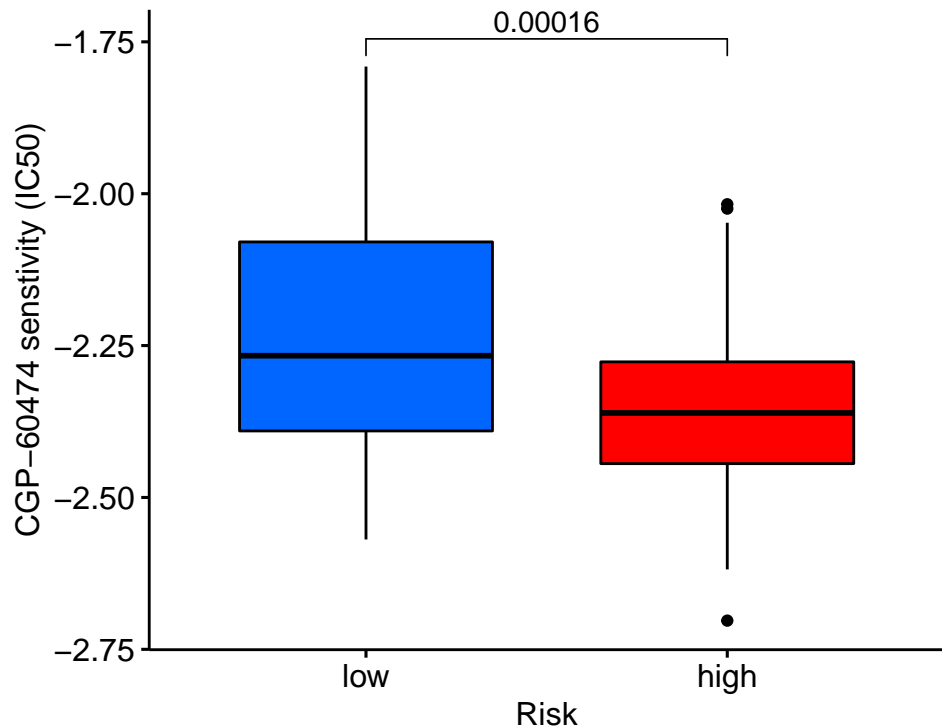

Supplement: Supplementary file 3 [file DataSheet2.zip › Raw Data2/pRRophetic/durgSenstivity.CGP-60474.pdf]

Risk 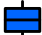 low 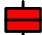 high

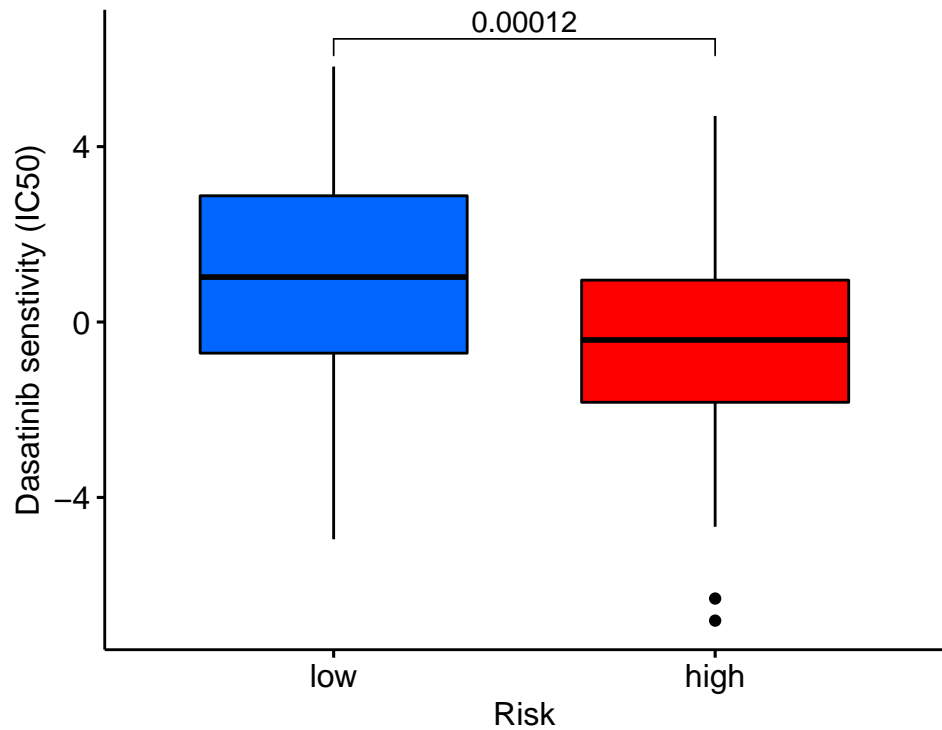

Supplement: Supplementary file 3 [file DataSheet2.zip › Raw Data2/pRRophetic/durgSenstivity.Dasatinib.pdf]

Risk 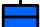 low 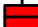 high

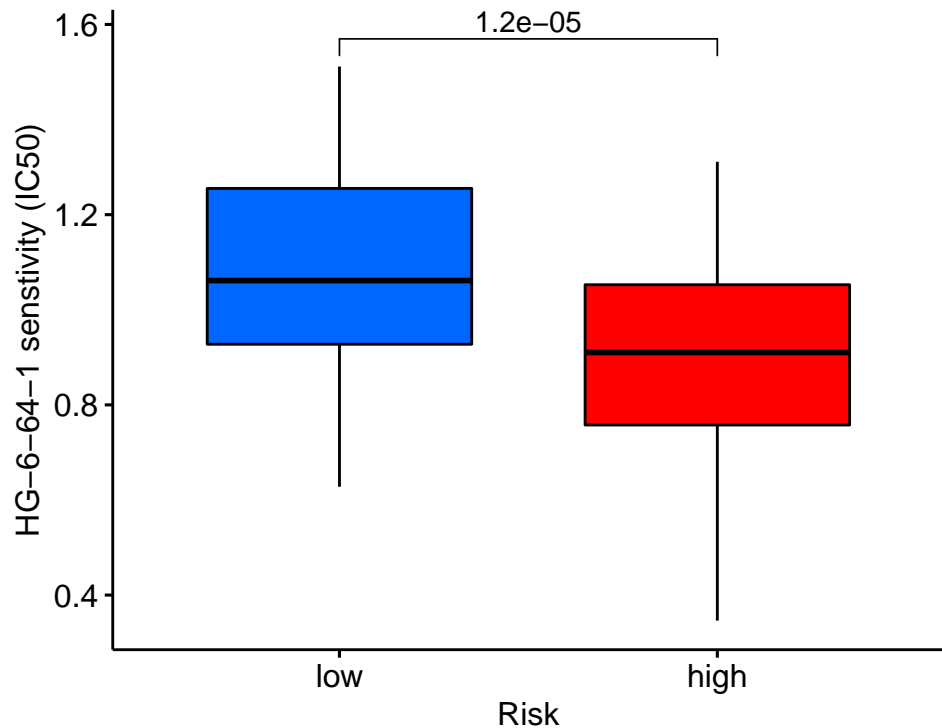

Supplement: Supplementary file 3 [file DataSheet2.zip › Raw Data2/pRRophetic/durgSenstivity.HG-6-64-1.pdf]

Risk 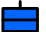 low 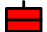 high

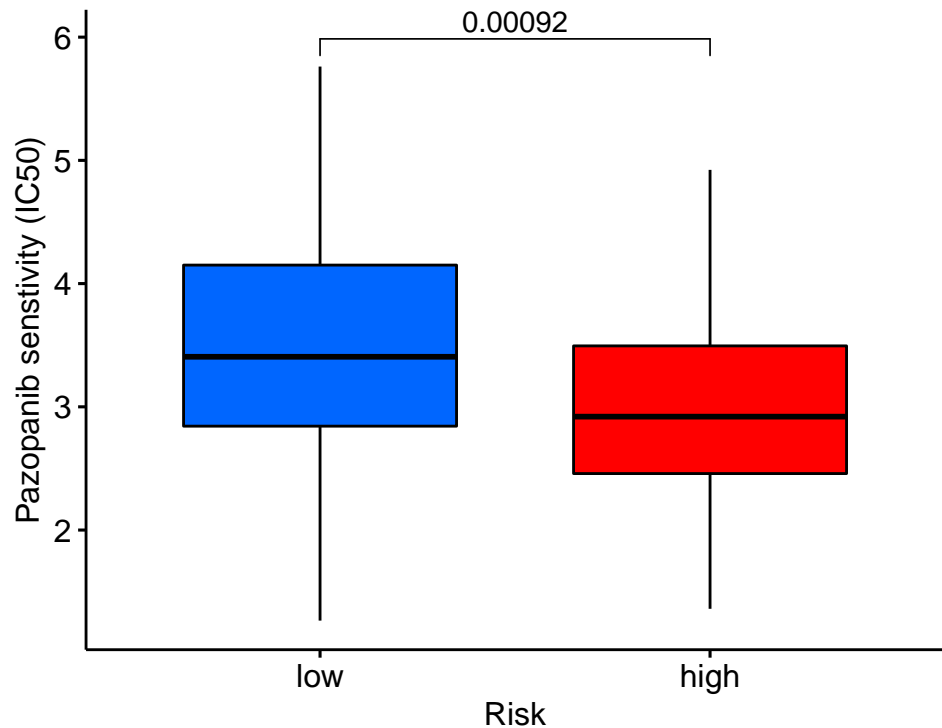

Supplement: Supplementary file 3 [file DataSheet2.zip › Raw Data2/pRRophetic/durgSenstivity.Pazopanib.pdf]

Risk 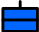 low 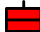 high

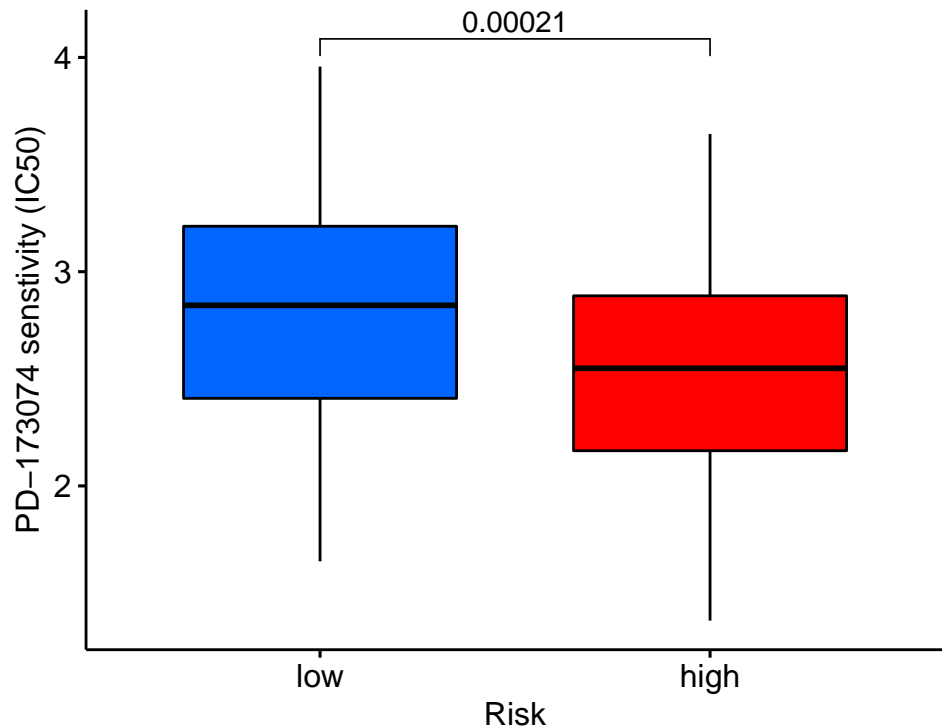

Supplement: Supplementary file 3 [file DataSheet2.zip › Raw Data2/pRRophetic/durgSenstivity.PD-173074.pdf]

Risk 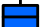 low 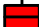 high

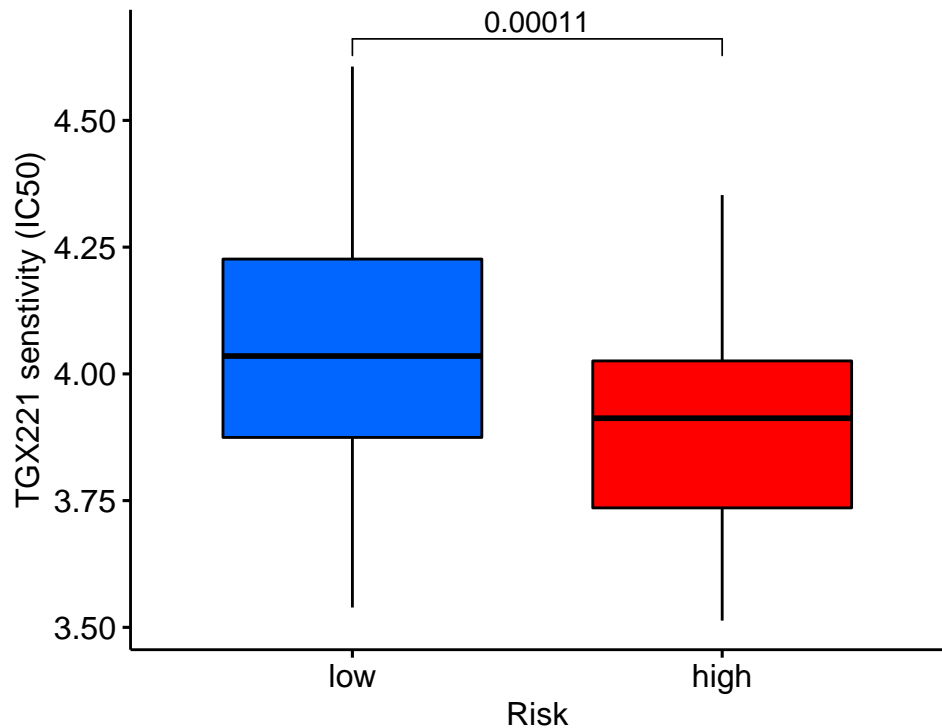

Supplement: Supplementary file 3 [file DataSheet2.zip › Raw Data2/pRRophetic/durgSenstivity.TGX221.pdf]

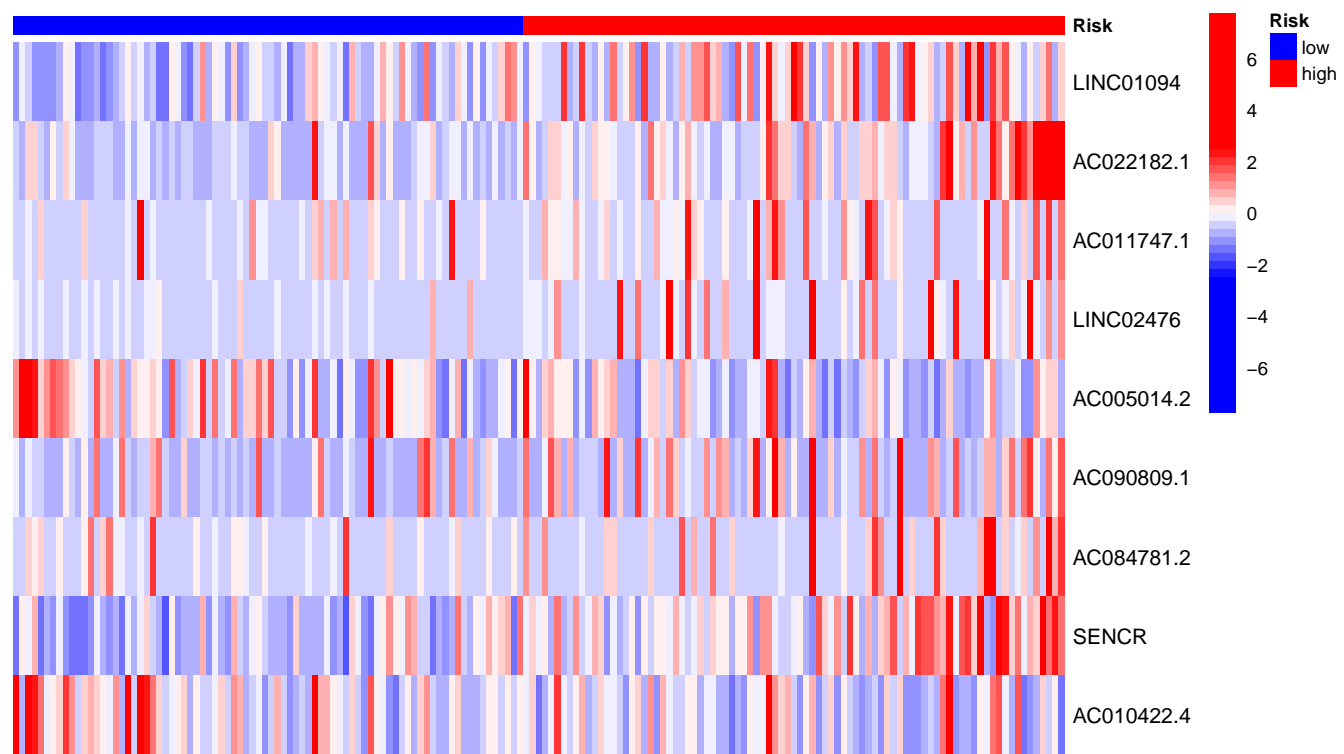

Supplement: Supplementary file 3 [file DataSheet2.zip › Raw Data2/riskPlot/all.heatmap.pdf]

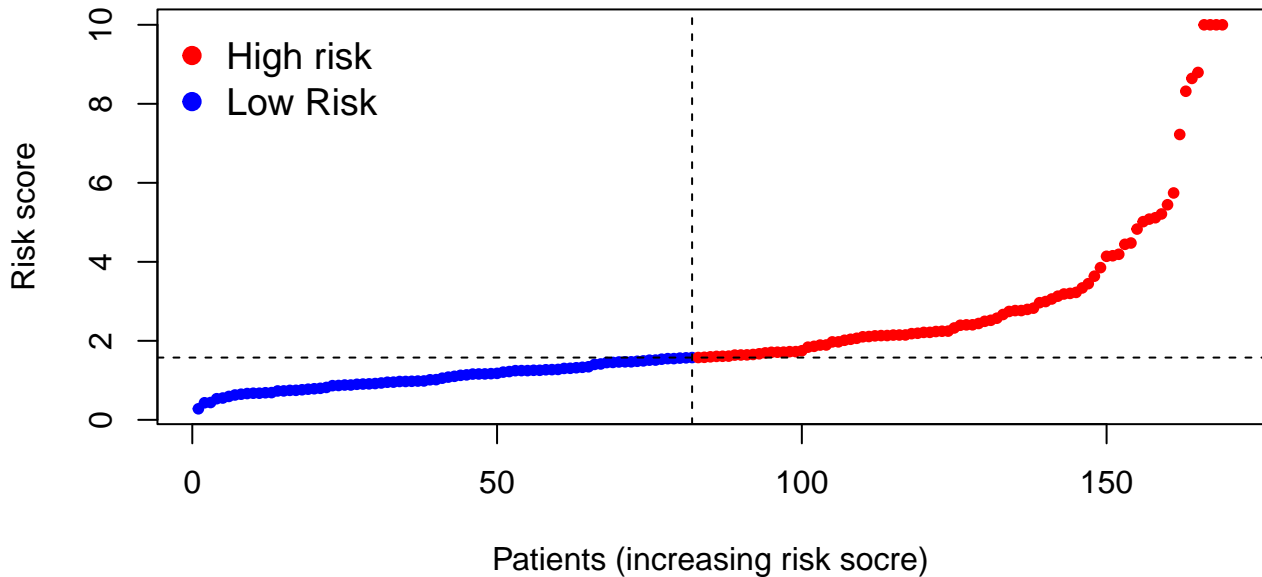

Supplement: Supplementary file 3 [file DataSheet2.zip › Raw Data2/riskPlot/all.riskScore.pdf]

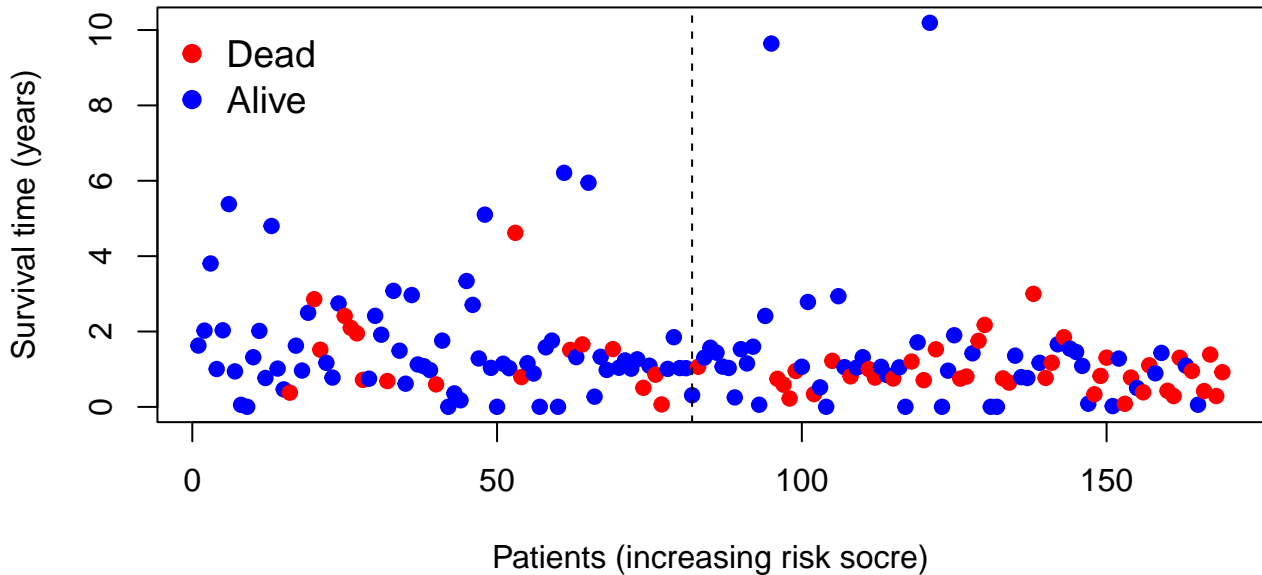

Supplement: Supplementary file 3 [file DataSheet2.zip › Raw Data2/riskPlot/all.survStat.pdf]

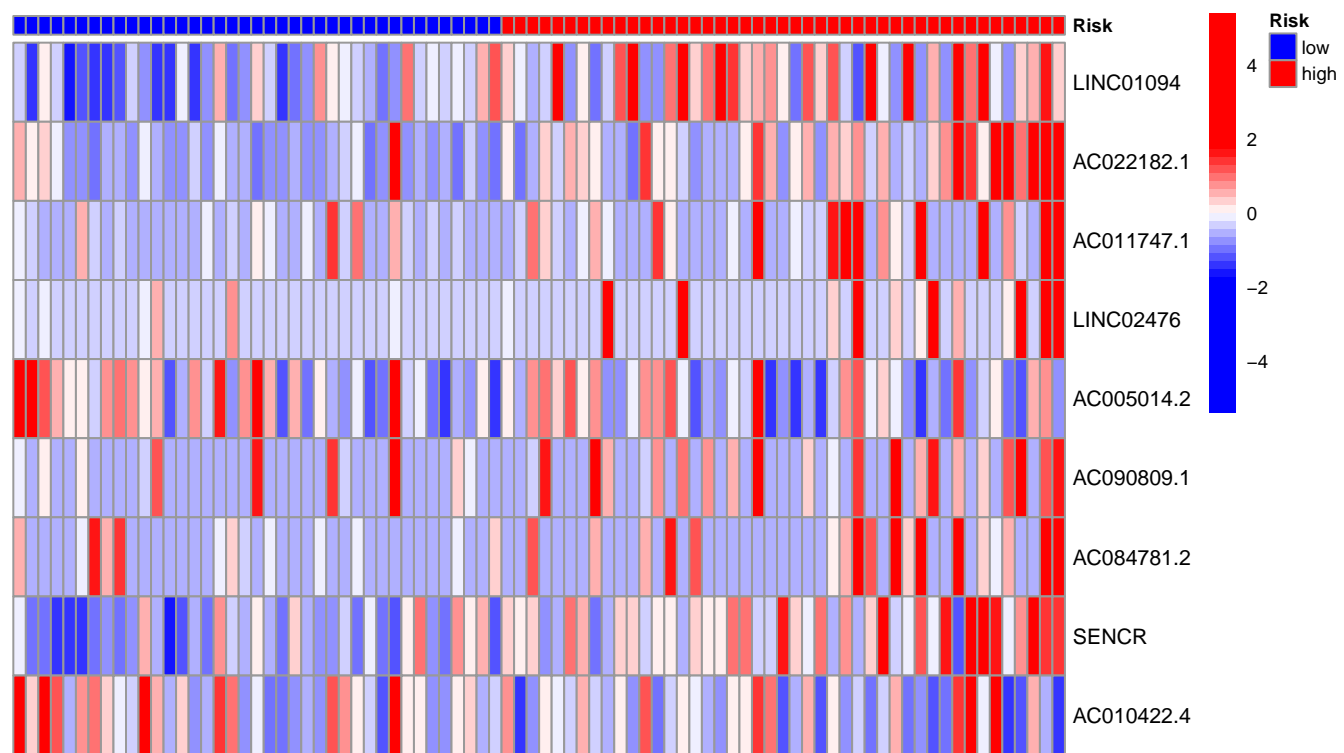

Supplement: Supplementary file 3 [file DataSheet2.zip › Raw Data2/riskPlot/test.heatmap.pdf]

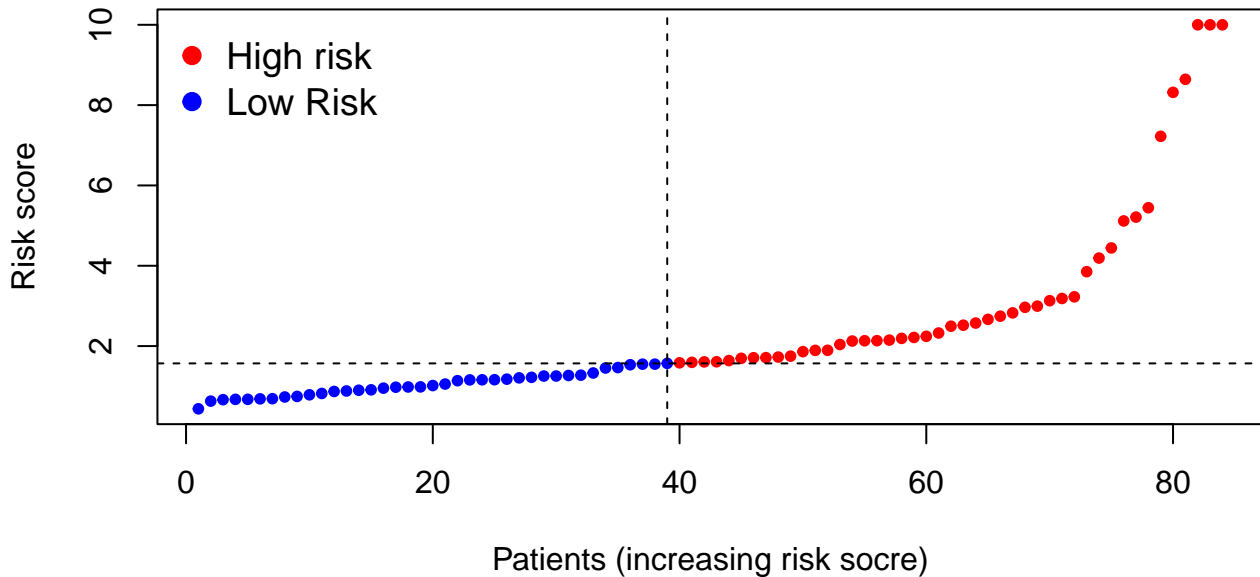

Supplement: Supplementary file 3 [file DataSheet2.zip › Raw Data2/riskPlot/test.riskScore.pdf]

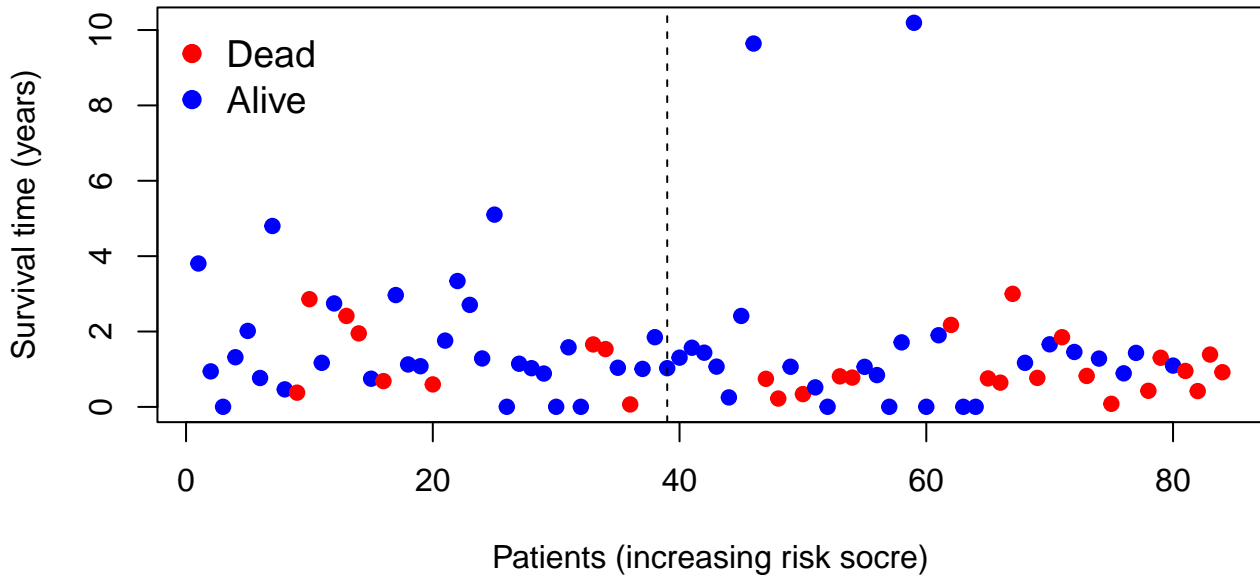

Supplement: Supplementary file 3 [file DataSheet2.zip › Raw Data2/riskPlot/test.survStat.pdf]

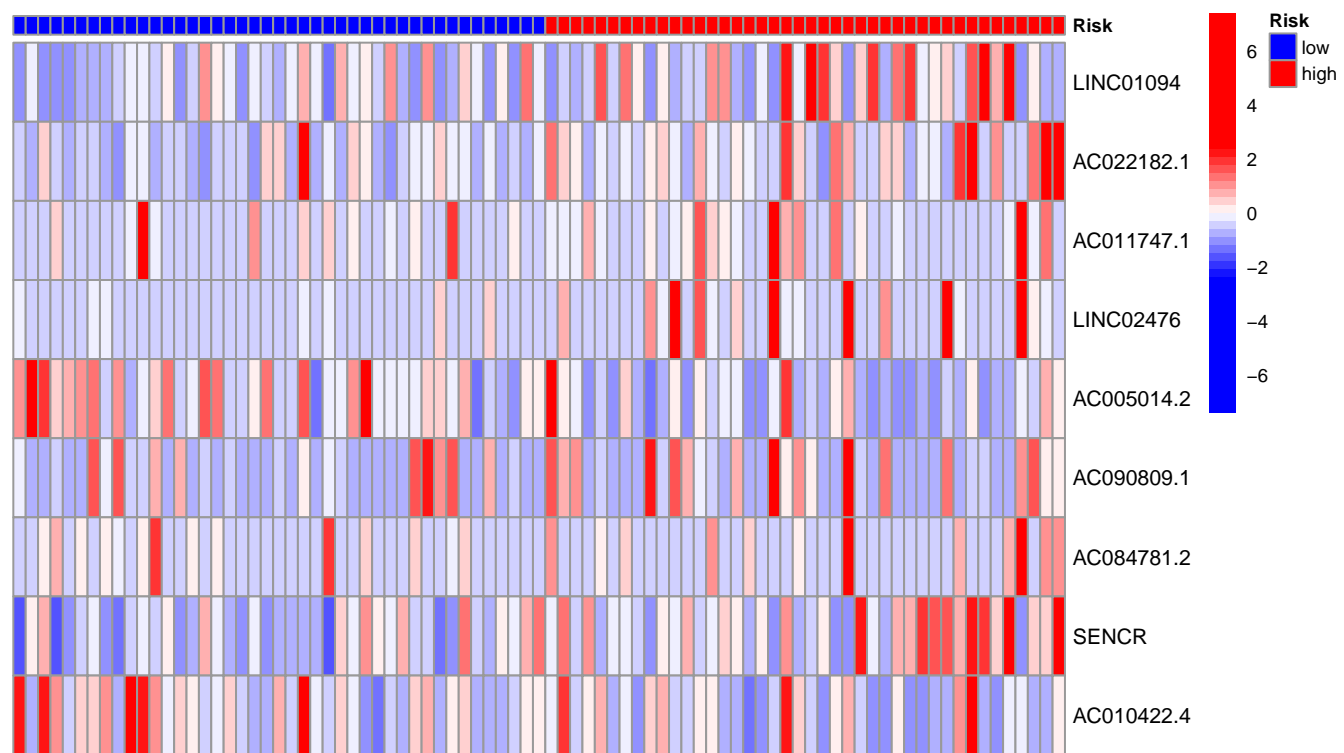

Supplement: Supplementary file 3 [file DataSheet2.zip › Raw Data2/riskPlot/train.heatmap.pdf]

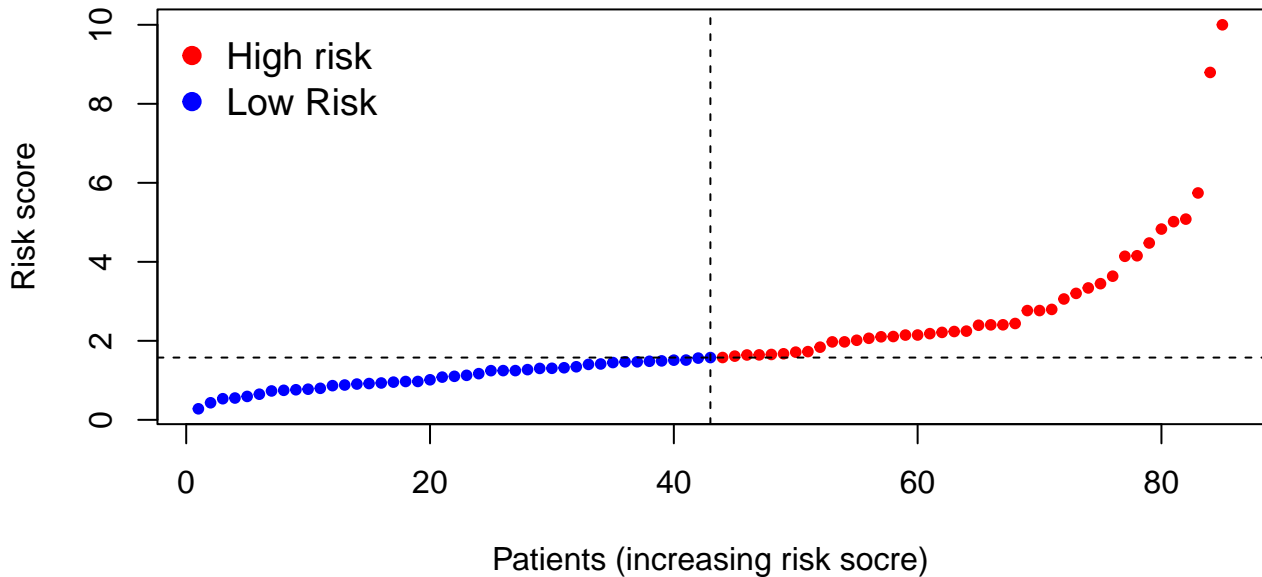

Supplement: Supplementary file 3 [file DataSheet2.zip › Raw Data2/riskPlot/train.riskScore.pdf]

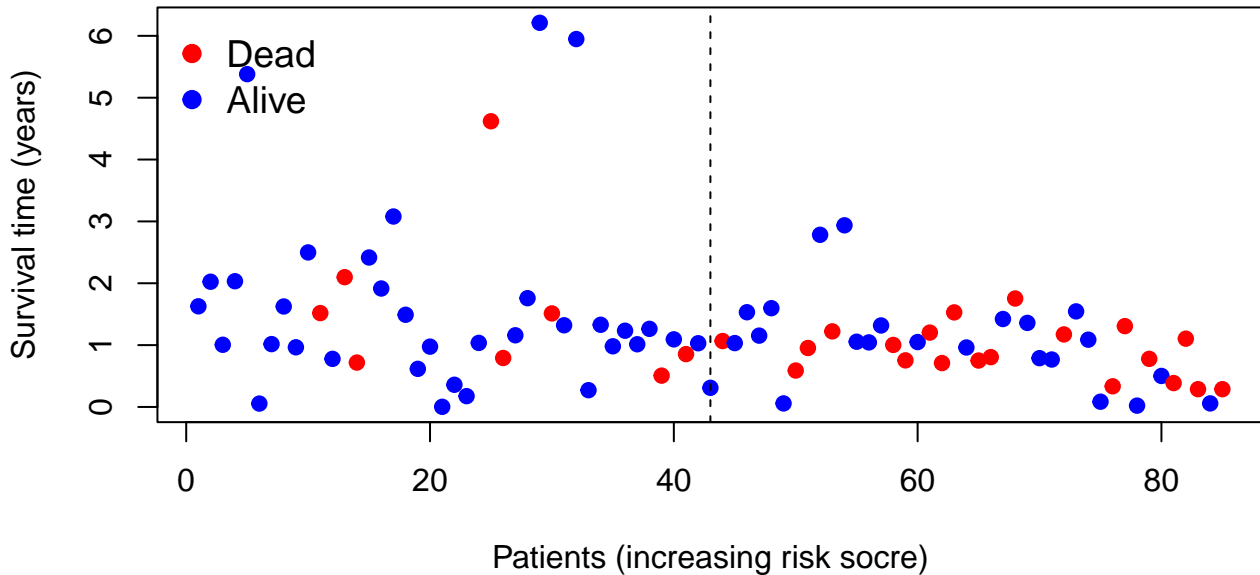

Supplement: Supplementary file 3 [file DataSheet2.zip › Raw Data2/riskPlot/train.survStat.pdf]

Low-risk High-risk

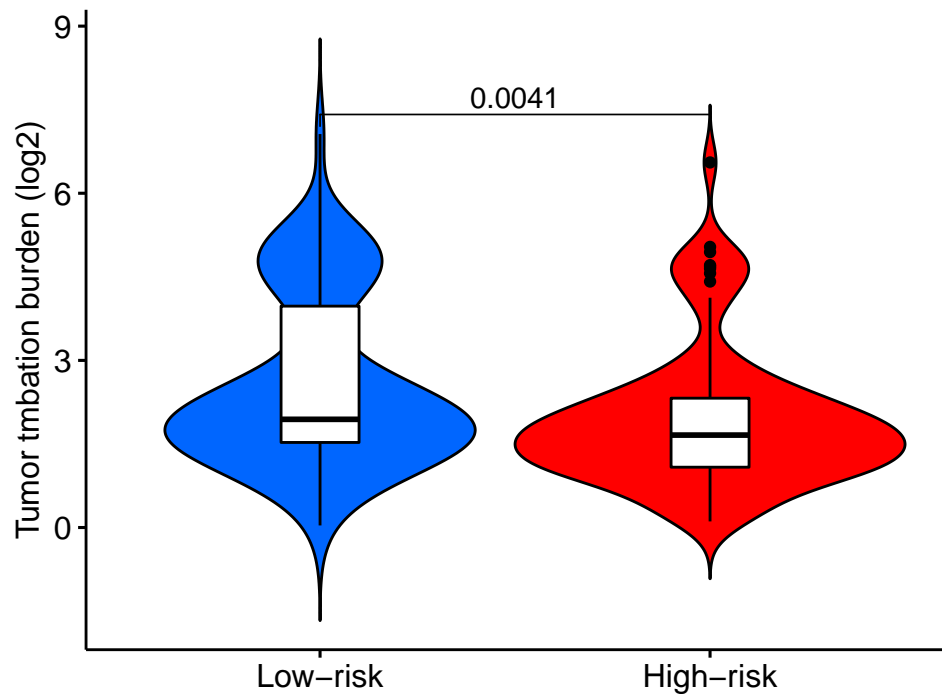

Supplement: Supplementary file 3 [file DataSheet2.zip › Raw Data2/riskTMB/riskTMB.pdf]

Sensitivity

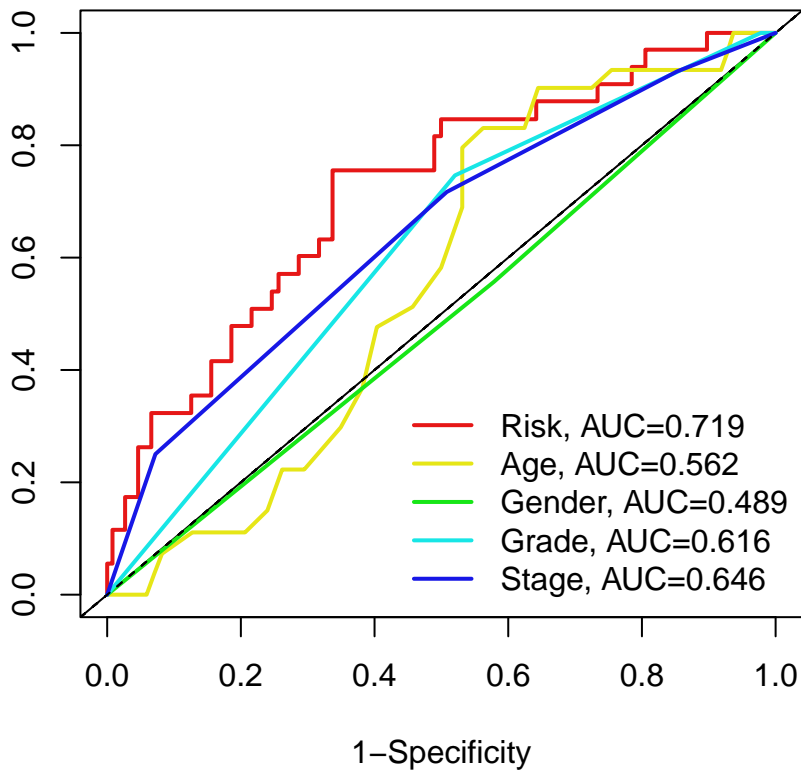

Supplement: Supplementary file 3 [file DataSheet2.zip › Raw Data2/ROC/cliROC.pdf]

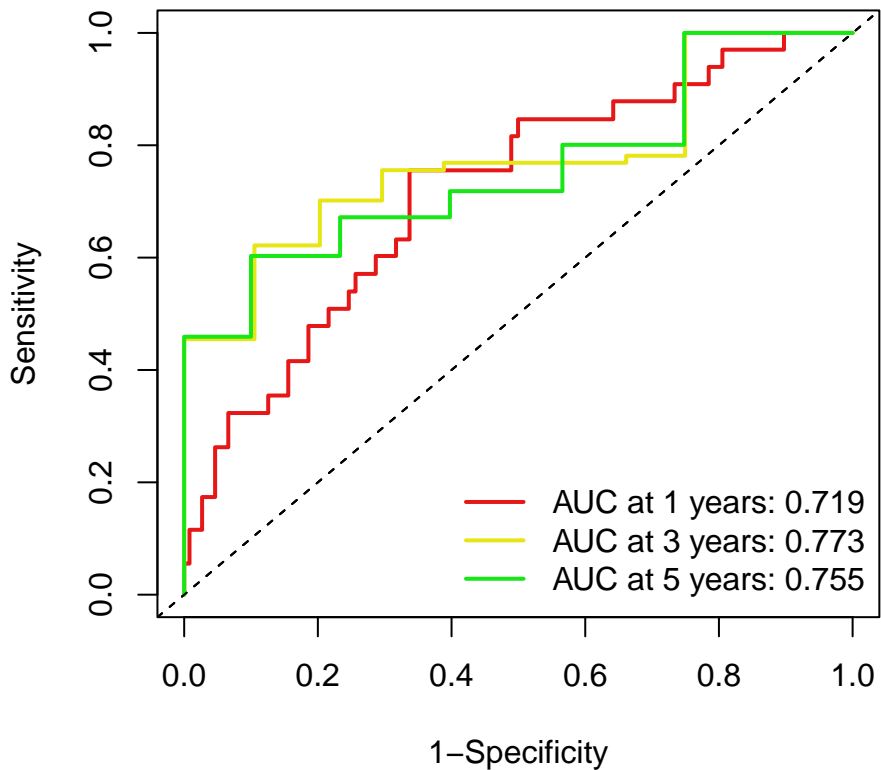

Supplement: Supplementary file 3 [file DataSheet2.zip › Raw Data2/ROC/ROC.pdf]

Risk + High risk + Low risk

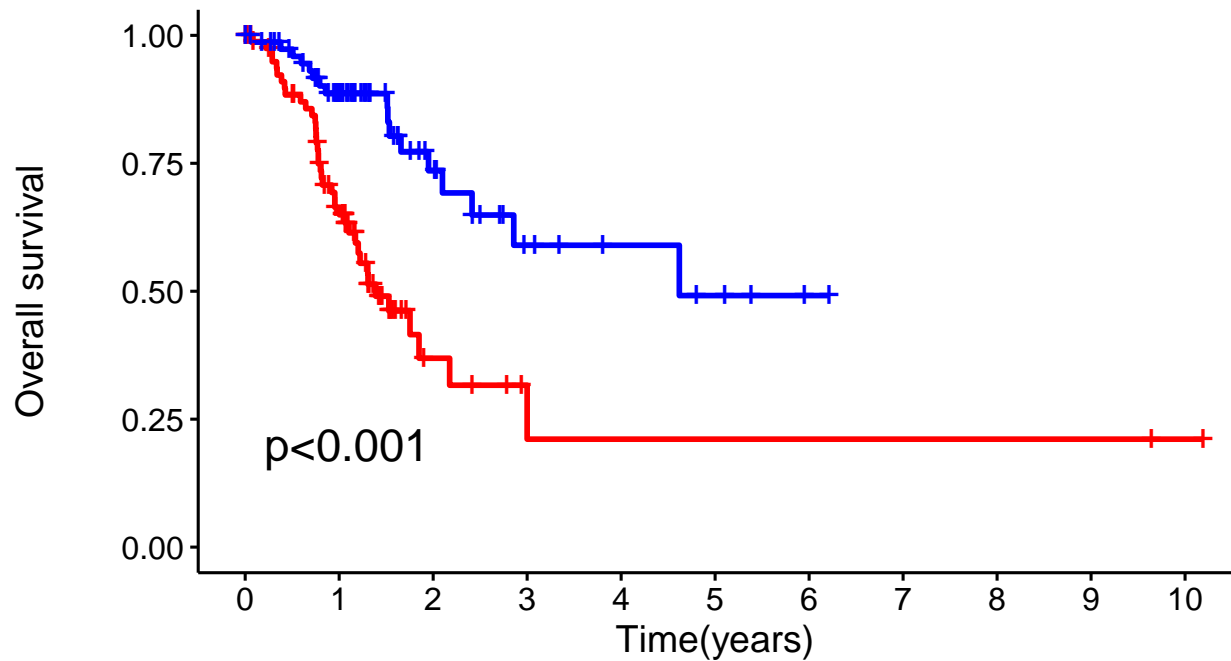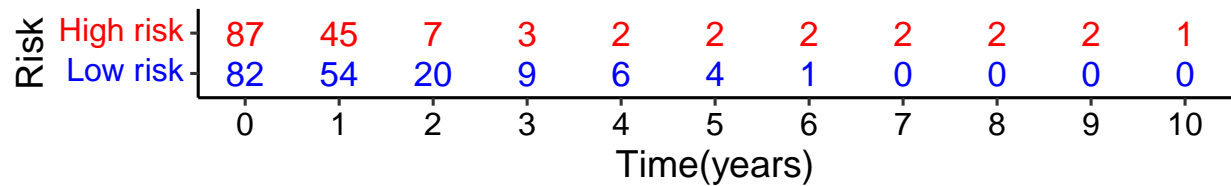

Supplement: Supplementary file 3 [file DataSheet2.zip › Raw Data2/survival/surv.all.pdf]

Risk    + High risk    + Low risk

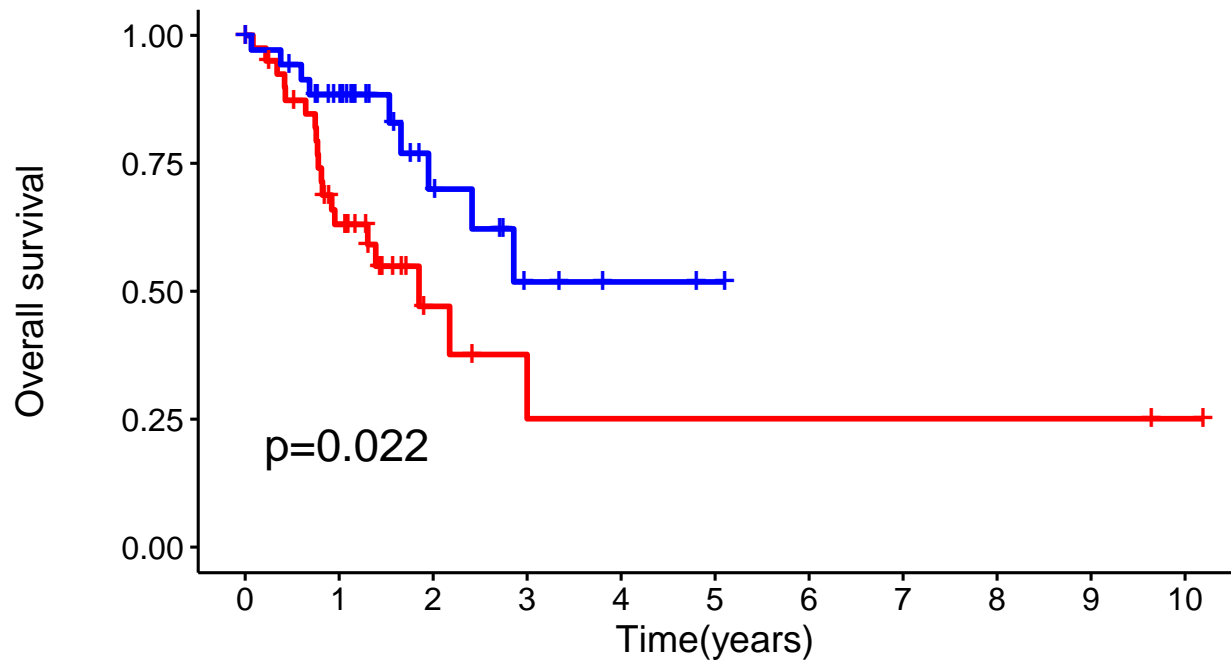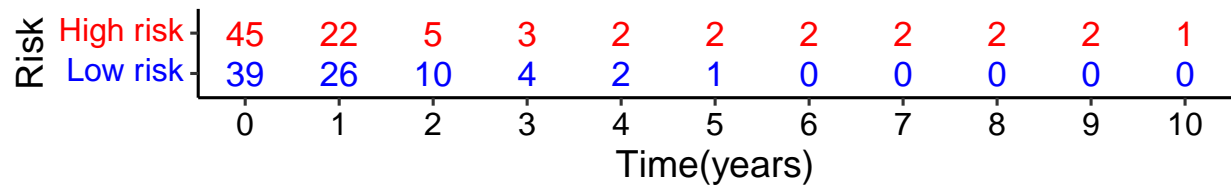

Supplement: Supplementary file 3 [file DataSheet2.zip › Raw Data2/survival/surv.test.pdf]

Risk + High risk + Low risk

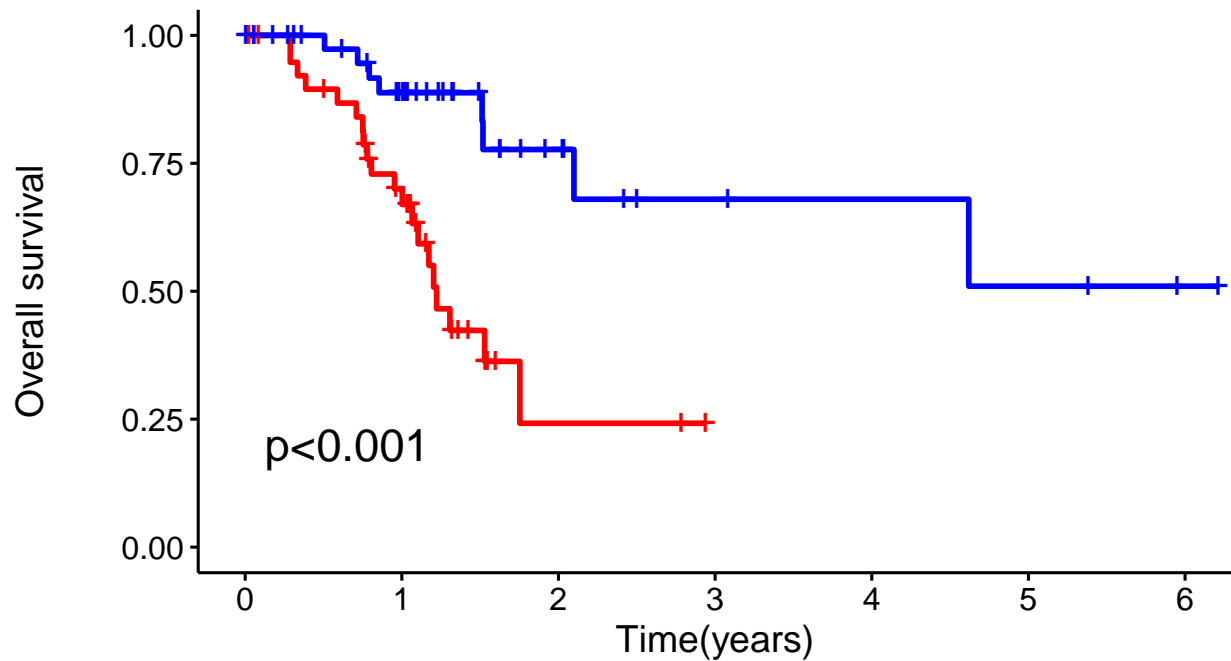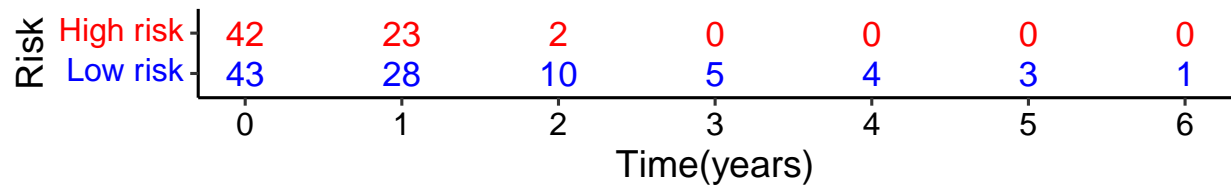

Supplement: Supplementary file 3 [file DataSheet2.zip › Raw Data2/survival/surv.train.pdf]

Risk 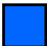 Low-risk 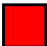 High-risk

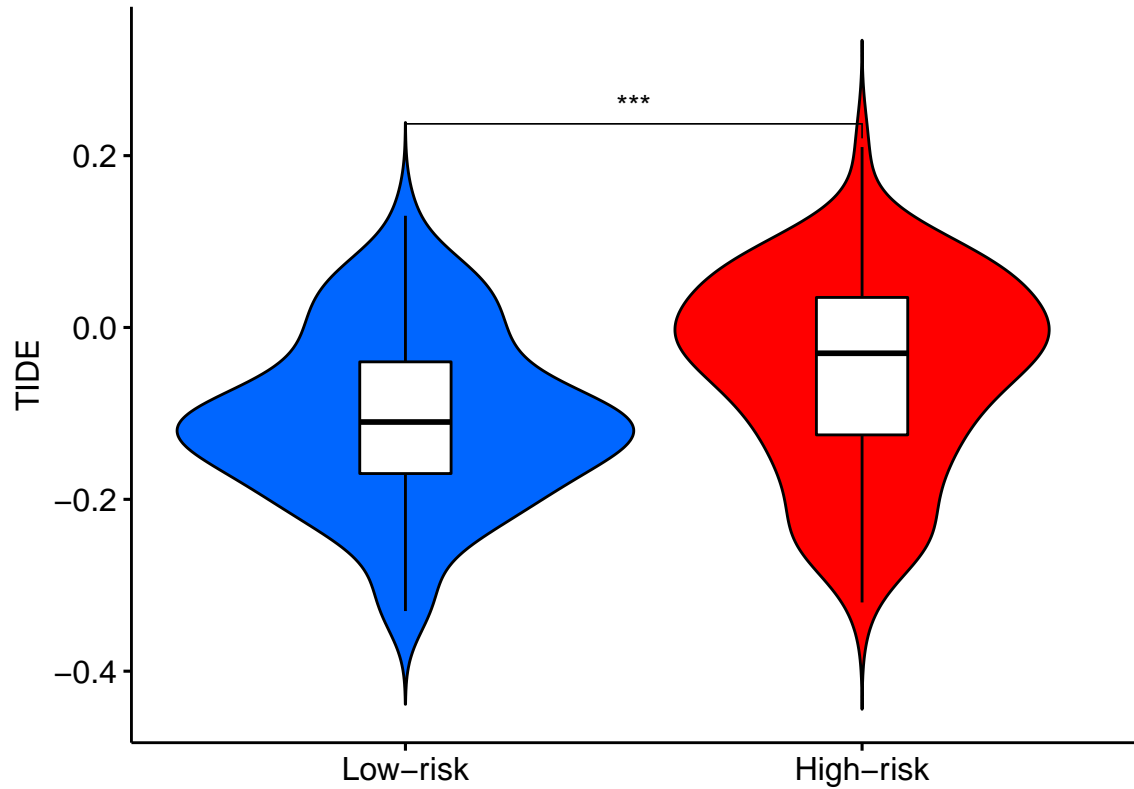

Supplement: Supplementary file 3 [file DataSheet2.zip › Raw Data2/TIDE/TIDE.pdf]

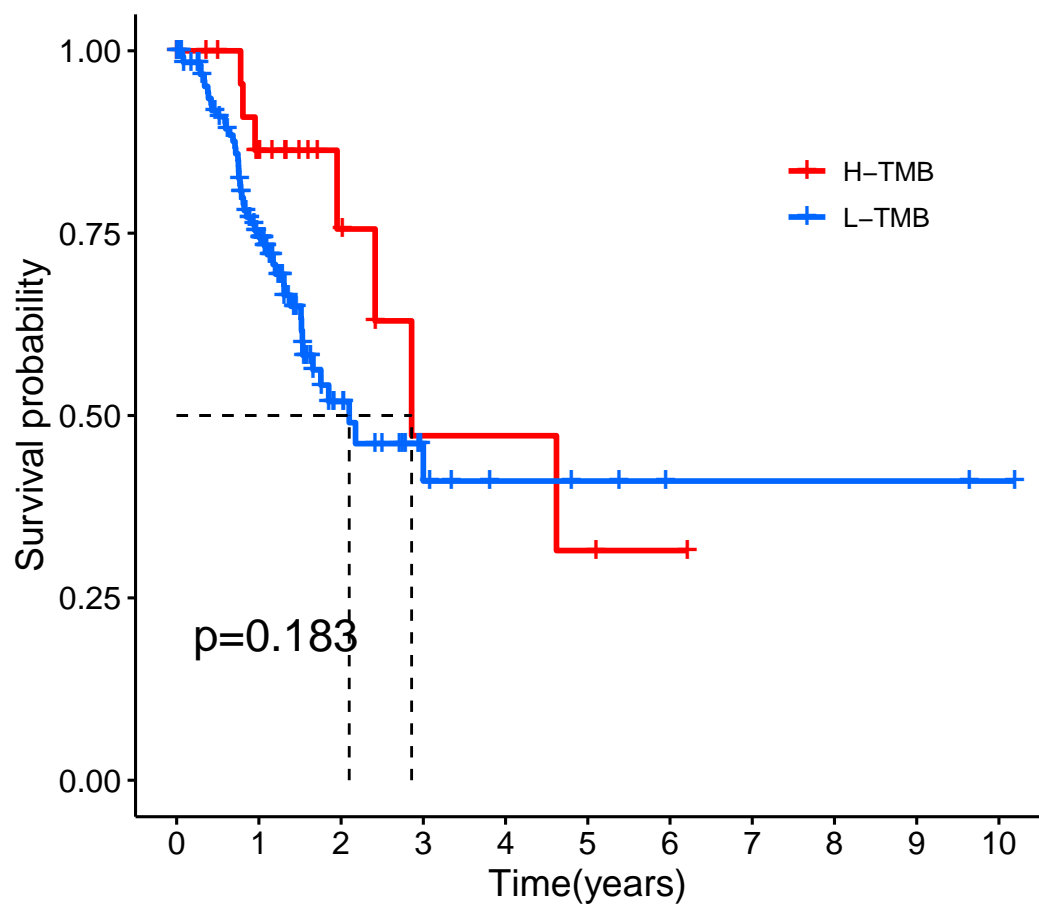

Supplement: Supplementary file 3 [file DataSheet2.zip › Raw Data2/tmbSur/TMB.survival.pdf]

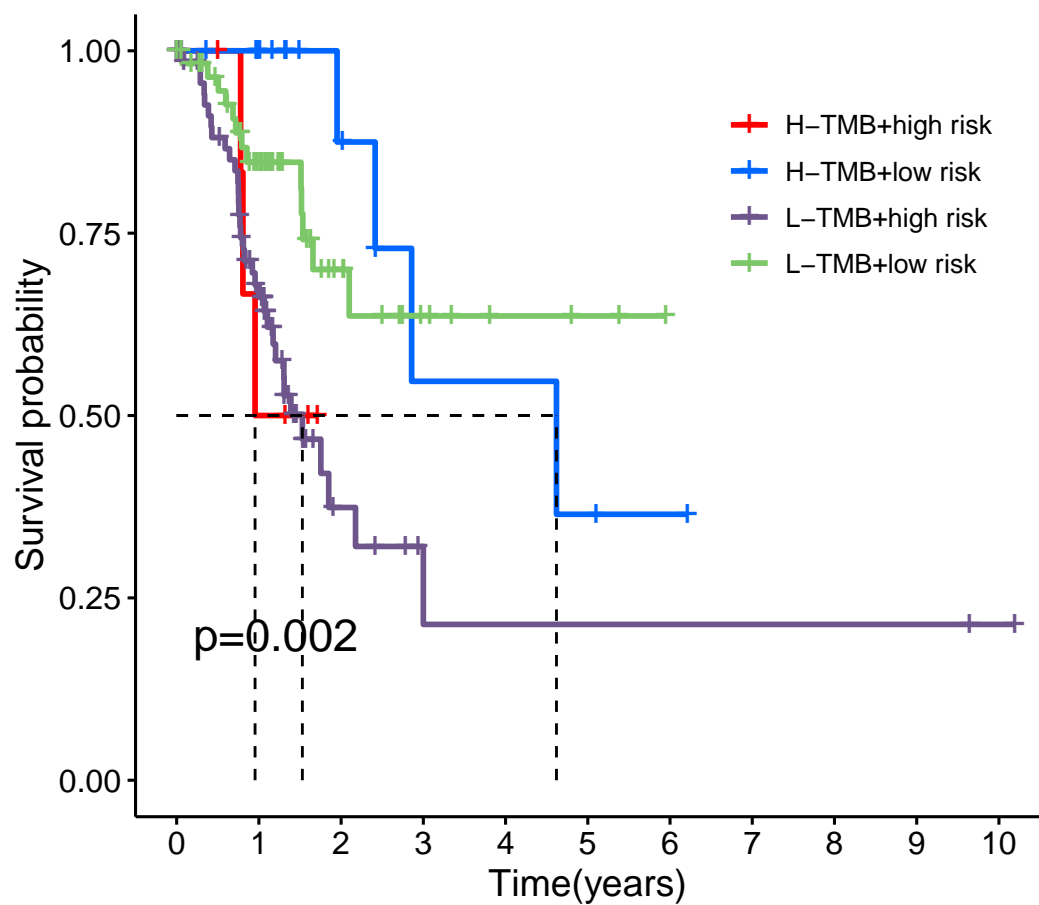

Supplement: Supplementary file 3 [file DataSheet2.zip › Raw Data2/tmbSur/TMB-risk.survival.pdf]
